# Characterization of germ cell differentiation in the male mouse through single-cell RNA sequencing

**DOI:** 10.1038/s41598-018-24725-0

**Published:** 2018-04-25

**Authors:** S. Lukassen, E. Bosch, A. B. Ekici, A. Winterpacht

**Affiliations:** Institute of Human Genetics, Friedrich-Alexander-Universität Erlangen-Nürnberg, 91054 Erlangen, Germany

## Abstract

Spermatogenesis in the mouse has been extensively studied for decades. Previous methods, such as histological staining or bulk transcriptome analysis, either lacked resolution at the single-cell level or were focused on a very narrowly defined set of factors. Here, we present the first comprehensive, unbiased single-cell transcriptomic view of mouse spermatogenesis. Our single-cell RNA-seq (scRNA-seq) data on over 2,500 cells from the mouse testis improves upon stage marker detection and validation, capturing the continuity of differentiation rather than artificially chosen stages. scRNA-seq also enables the analysis of rare cell populations masked in bulk sequencing data and reveals new insights into the regulation of sex chromosomes during spermatogenesis. Our data provide the basis for further studies in the field, for the first time providing a high-resolution reference of transcriptional processes during mouse spermatogenesis.

## Introduction

Mammalian spermatogenesis is one of the most efficient cell-producing processes in adult mammals and an excellent model for studying stem cell renewal and cell differentiation. Defects in this well-controlled process cause male infertility, which accounts for approximately half of all infertility and results from genetic abnormalities in 15–30% of cases^1,2^. This complex process takes place in the seminiferous tubules of the testis, which are almost exclusively comprised of germ cells. In addition to undifferentiated spermatogonial stem cells (SSCs) and mature spermatozoa, all other germ cells in the adult testis represent transitional stages in the continuous process of germ cell differentiation. This has made gene expression studies challenging.

Two different approaches have been used to date. The first approach analyzes bulk RNA from testes of prepubertal animals at different time points during the first wave of spermatogenesis^3–5^. In this approach, it is hard to attribute RNAs to proper cell populations, and the results may not be translatable to adult tissues. The second approach is the enrichment of different cell populations using different techniques^6–8^. While some of these methods skew the results of subsequent gene expression analyses, others require large amounts of starting material, resulting in relatively low-purity samples, or are only applicable to certain cell types^9^. All enrichment methods use defined surface markers or parameters (e.g., size, DNA content) specific for certain cell populations, a strategy that is highly biased and does not reflect the continuous nature of male germ cell differentiation. Recent advances in single-cell RNA sequencing (scRNA-seq) enable a broad transcriptome characterization of thousands of heterogeneous single cells in a population, reflecting the biological complexity of a certain tissue. Very recently, scRNA-seq has already been successfully used for unbiased single cell transcriptome analysis allowing the identification of novel cell types or tumor subclasses and providing insights into regulatory networks of differentiation^10–13^.

Here, for the first time, we employed scRNA-seq to establish expression profiles of 2,550 germ cells from the adult mouse testis. The present data impressively demonstrate the continuous, dynamic and heterogeneous differentiation process during murine spermatogenesis. We show that scRNA-seq is a powerful tool for the investigation of differentiation networks even in rare cell populations and the regulation of sex chromosomes during spermatogenesis in high-resolution.

## Results

**Figure 1:**
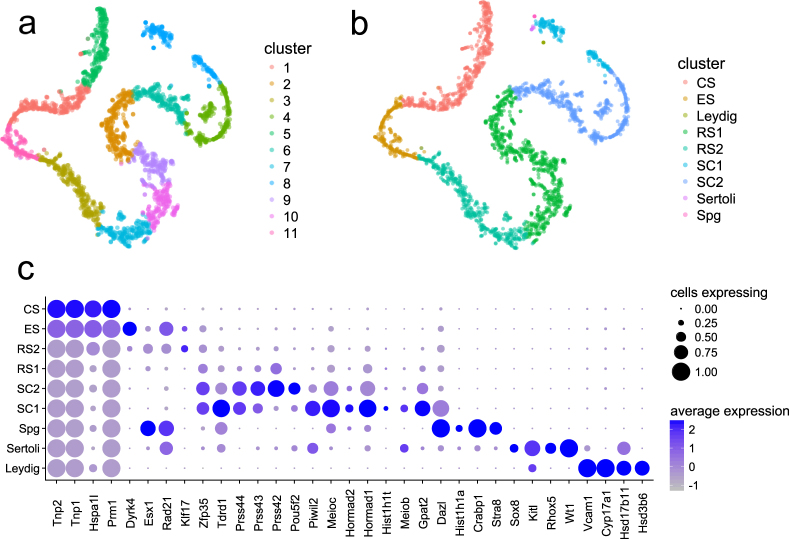
(**a** and **b**) T-SNE projection of cells from both replicates, colored by cluster identity obtained from (**a**) graph-based and (**b**) K-means clustering with K = 9. Cell types were assigned through the expression of representative marker genes (Supplementary data Table S3, subfigure c) (**c**). Dot plot of proportion of cells in the respective K-means cluster expressing each marker (dot size), and average expression (color scale). Spg = spermatogonia, SC = spermatocytes, RS = round spermatids, ES = elongating spermatids, CS = condensing spermatids.

To obtain single-cell expression profiles for a large number of testicular cells, we prepared cell suspensions from the testes of two 8-week-old C57BL/6J mice and obtained transcriptomes for approximately 1250 cells for each mouse. To keep biological noise to a minimum and assess the variation introduced by the technique rather than different litters or strains, we used littermates. To assess the reproducibility of our approach, we compared both mice in terms of sequencing statistics, presence of cell populations, and differential gene expression. The mice were virtually indistinguishable in any QC statistic and yielded identical distributions after t-stochastic neighbor embedding (t-SNE) (Supplementary Fig. S1). Automated, graph-based clustering revealed 11 clusters, all of which were present in both replicates (Supplementary data Table S1). In mouse 1 and 2, there were two and twenty genes upregulated, respectively, whereas 3749 genes significantly altered their expression with differentiation stage in a pseudotime analysis in both mice.

t-SNE revealed cells to be arranged in a continuous succession rather than in clusters. This is markedly different from other studies on cultured cells or adult somatic tissues but is not surprising given that most cell types in the testis represent transitionary stages^14^. The order of cells in t-SNE reflects the different successive stages of spermatogenesis, with pre-meiotic cells located at the top right in the visualization presented here (Fig. 1a and b). Two different clustering methods were employed to enable cell type detection. Graph-based clustering led to the identification of 11 clusters of roughly equal size and did not capture rare cell populations very well (Fig. 1a). This was ameliorated using K-means clustering with K = 9, which led to the accurate detection of cell populations as demonstrated by the expression profiles of individual clusters (Fig. 1b and c). The expression of over 200 published spermatogenesis stage markers was plotted along the different clusters identified through K-means clustering, resulting in a distribution consistent with previous literature findings (Supplementary Figs S2 and S3 and Supplementary data Table S2). A representative selection of markers for the different cell populations is shown in Fig. 1c (Supplementary data Table S2). While early and late stages of spermatogenesis were well defined by known markers, there was a gap in marker gene expression around meiosis II.

In addition, already published RNA-seq data for sorted testicular cell populations^15,16^ overlapped nicely with the abundant cell types in our data (spermatocytes, and round, elongating and condensing spermatids; Supplementary Fig. S4). The RNA-seq profiles previously identified for spermatogonia, however, overlapped with the somatic cell populations in the present dataset. In both studies, spermatogonia were isolated from the testes of 6 dpp^15,16^ or 8 dpp^15^ mice with an estimated purity of 85–90%. Gan *et al*. stated Sertoli cells as the most likely contaminant in their cell populations, which is consistent with the results presented here. The discrepancies between the datasets could also stem from the different time points of isolation, as spermatogonia are regulated differently between juvenile mice undergoing the first wave of spermatogenesis and adult testes^17^.

Given the continuity observed, this subdivision of clusters, especially during and after meiosis, serves as a rough guide rather than a definite cell type assignment, but nicely illustrates the *in vivo* organization of cells being conserved in the *in silico* analysis. Even without aligning, the distribution of cells along the t-SNE plot did not differ between the two biological replicates (Supplementary Fig. S1). The continuity of expression changes could be confirmed using unsupervised pseudotime analysis. Comparison of pseudotime ranks to the results obtained from the analysis of clusters in the t-SNE projection revealed almost perfect agreement (Supplementary Fig. S5).

**Figure 2:**
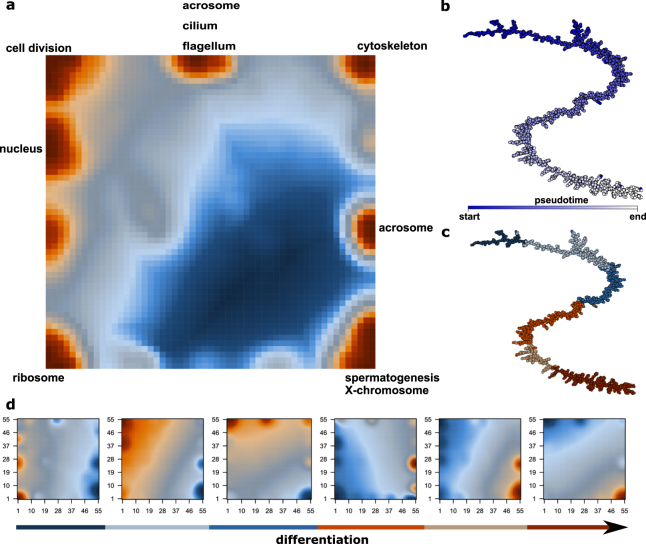
(**a**) SOM portrait indicating metagenes overexpressed at any stage during germ cell differentiation. Examples for gene sets highly enriched in the corresponding metagenes are indicated at the sides of the SOM. (**b** and **c**) Correlation spanning tree based on metagene expression with pseudotime (**b**) or K-means clustering and (**c**) color coding. (**d**) SOM portraits for the different K-means clusters indicated in (**c**), ordered by pseudotime (**b**). The positioning of the metagenes is identical to that in (**a**).

Many widely accepted markers were broadly expressed. We could identify genes that were highly specific for certain cell populations, many of which had not yet been annotated with a gene function (Supplementary data Table S4). These markers included lncRNAs, as well as Riken cDNAs. Interestingly, ribosomal protein and nuclear mitochondrial protein genes showed a strong differential expression correlating with pseudotime and were key parts of the crucial metagenes in self-organizing map (SOM) analysis (Fig. 2).

Some cell populations within the testis, such as undifferentiated spermatogonia, Sertoli cells, and Leydig cells, are very small. Furthermore, large cells or those in tight contact with neighboring cells may be further depleted through the application of cell strainers in the preparation of single-cell suspensions, which is necessary to eliminate doublets. Neither of the small cell populations is accurately captured by pseudotime analysis or graph-based clustering. K-means clustering with K = 9 was sufficient to identify spermatogonia (6 cells/0.23%, expressing *Stra8*, *Crabp1*, *Hist1h1a*, and *Dazl*), Sertoli cells (7 cells/0.23%, expressing *Wt1*, *Rhox5*, *Kitl*, and *Sox8*) and Leydig cells (5 cells/0.20%, expressing *Hsd3b6*, *Hsd17b11*, *Cyp17a1*, and *Vcam1*). Differential gene expression revealed 21 potential marker genes for spermatogonia, 24 for Sertoli cells, and 20 for Leydig cells. These genes were significantly (p < 0.01) upregulated (log2 fold change > 7) in the respective population (Supplementary data Table S5). Due to the small number of spermatogonia captured in this dataset and the low abundance of stem cells in the testis, it is not entirely certain whether the population in this dataset includes undifferentiated spermatogonia or spermatogonial stem cells.

**Figure 3:**
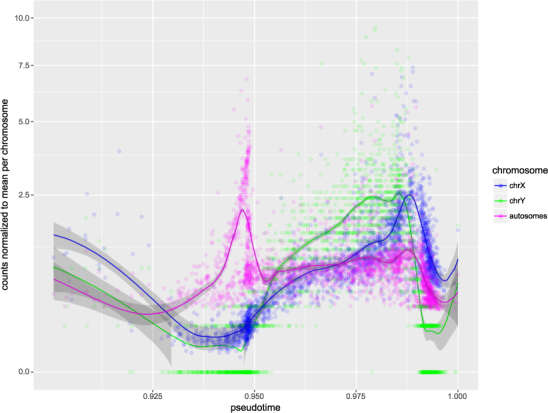
Read counts for autosomes and sex chromosomes, centered on the mean for the respective chromosome. Individual cells are represented as circles, with smoothed local regression (Loess) represented as solid lines. Shaded areas indicate the standard error of the regression fit. Meiosis occurs roughly between the 0.925 and the 0.95 mark, while *Prm1* transcription and thus histone-to-protamine exchange is initiated at approximately 0.986. The fastest decay of the respective transcripts could be observed at 0.991 (Y chromosome) and 0.992 (X chromosome and autosomes).

Read counts for the sex chromosomes, compared to autosomes, displayed a pattern in agreement with meiotic sex chromosome inactivation (MSCI). Interestingly, while the X and Y chromosome displayed similar transcript regulation during meiosis, the X chromosomal transcripts were markedly more similar to autosomal ones during later stages of spermatogenesis. Y chromosomal transcript levels decreased before the onset of protamine expression, compared to a later decrease in X chromosomal and autosomal transcripts, and did so at a faster rate (Fig. 3). The estimated half-life at the point of maximum decay was 7.5 hours for the Y chromosome, compared to 26 and 24.7 hours for the X chromosome and autosomes, respectively (for details see Material and Methods). This indicates an increased persistence of X chromosomal transcripts in haploid cells, while Y chromosomal RNAs appear to be rapidly degraded. The expression patterns of most genes on the Y chromosome were extremely homogeneous, with a peak expression after meiosis I and a decrease in transcript levels at the early spermatid stage. For the X chromosome, no consistent pattern could be discerned (Supplementary Fig. S6). In general, the expression of genes on the Y chromosome was weaker than that of X chromosomal genes, with the highest expressing X chromosomal gene showing an almost tenfold higher expression than the most active Y chromosomal gene. Weak expression of Y chromosomal genes is a well-described phenomenon^18^.

**Figure 4:**
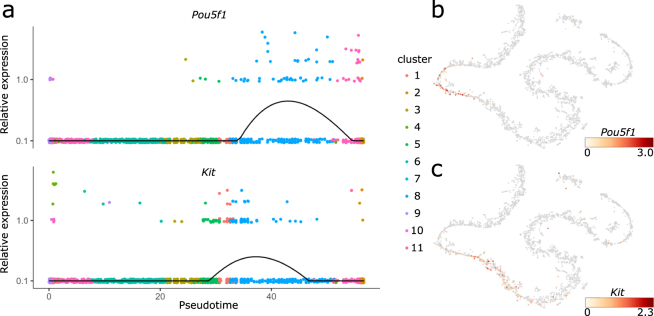
(**a**) Expression values of *Pou5f1* and *Kit* along the pseudotime axis. The black line denotes the smoothed, average expression. (**b** and **c**) t-SNE projections with colors indicating the expression of *Pou5f1* (**b**) and *Kit* (**c**).

While cell populations for which the expression of certain proteins had been described generally showed transcription of the corresponding RNA, the reverse was not necessarily true. One remarkable example was *Oct4* (*Pou5f1*), which was present at high levels in round spermatids, while its protein expression was reported to be exclusively found in spermatogonial stem cells^19^ (Fig. 4a). This discrepancy is consistent with published literature showing expression, but not translation, of *Oct4* in spermatids^20^ and highlights the importance of differentiating between RNA and protein expression. A strong discrepancy between transcription and translation during meiosis has been shown for other organisms, such as yeast^21^. *Kit*, which is expressed in spermatogonia, is another example. Our data show that *Kit* is also expressed at a later stage of spermatogenesis, probably in round spermatids (Fig. 4b). This coincides with published results demonstrating the expression of a truncated *Kit* product (tr-kit) in post-meiotic stages of spermatogenesis^22^.

Both mice showed highly consistent results, indicating that even for highly complex transcriptomes such as that of the mouse germ line, analysis of only few biological replicates can be sufficient. This is likely aided by the genetic homogeneity of inbred mice, but as gametogenesis is a highly controlled and conserved process, the results are expected to be transferrable between mouse strains.

The high degree of consistency and evenness of marker expression in the pseudotime analysis indicates that scRNA-seq overcomes at least some limitations of the methods mentioned before.

## Discussion

Our results demonstrate that male germ cells differentiate in a way that is highly continuous, with no clear-cut differences between transcriptomes of succeeding cell types. This highlights the inability of marker-based techniques such as FACS to capture the entire dynamics of spermatogenesis.

The fairly small number of 6 spermatogonia, distinguished by the expression of the cell markers (*Stra8*, *Crabp1*, *Hist1h1a*, and *Dazl*) is to be expected, since the estimated total number of undifferentiated spermatogonia in the adult mouse testis is extremely low^23^. Of note, in the present study we investigated only 1250 cells per mouse, although the maximum number of cells analyzable by the procedure (Chromium) is 10,000 per run, which would, in principle, allow a more detailed investigation of the spermatogonial population with this procedure.

Furthermore, we confirm findings concerning divergence between gene and protein expression, highlighting both the validity of our approach and the presence of an additional layer of regulation not captured by protein expression. This is in part due to the expression of non-coding isoforms of genes such as *Pou5f1*, which have an unknown significance. Characterization of these alternate transcripts, however, is currently restricted to bulk methods, as the read counts per cell in current scRNA-seq approaches are too low to robustly detect these events. Furthermore, most splice-sites are not covered by the 3′ sequencing method used.

The number of significantly regulated non-coding transcripts strongly emphasizes the importance of regulatory RNAs for germ cell differentiation, a field which has not been explored in much detail so far but can now be analyzed in depth by scRNA-seq. Moreover, the gene lists also include numerous uncharacterized Riken cDNAs, as well as genes currently not associated with a function in spermatogenesis, thus representing a source of novel biomarkers for male germ cell differentiation.

Interestingly, ribosomal protein (RP) genes and nuclear mitochondrial protein genes showed a strong differential expression correlating with pseudotime and were key markers for the different groups. This observation is intriguing since tissue-specific roles of RPs in development and disease have been reported recently^24,25^ and changes in mitochondrial shape, number and function are known to be important for normal spermatogenesis^26,27^. scRNA-seq offers the possibility to investigate the dynamic changes of these interesting factors during normal and disturbed spermatogenesis in much more detail.

The postmeiotic expression patterns observed for the X and Y chromosome are consistent with recent literature findings^18^. As half of the haploid spermatid population lacks an X or a Y chromosome, these cells cannot produce new transcripts from these chromosomes. Thus, the earlier and faster decay of Y chromosomal transcripts, in conjunction with lower transcript counts, indicates an absence of these RNAs from the sperm. X chromosomal transcripts, on the other hand, are more abundant and more stable, and may thus be transmitted to the zygote. A higher stability of X chromosomal RNA than autosomal RNA has been reported for both male and female human and mouse cell lines^28^. Our results indicate a slight increase in the stability of X chromosomal transcripts compared to autosomal transcripts but marked differences between the sex chromosomes. With respect to the Y chromosome, this is the first study demonstrating such an effect. As to the spermatids, our results are remarkable, since up to now postmeiotic expression of spermatid-specific genes on the sex chromosomes has only been attributed to epigenetic re-activation of the sex chromatin^18^ but not RNA stability. This result nicely demonstrates the power of single cell transcriptome analysis.

Further insights could be gained in the future by analyzing mouse models with a block at various stages during spermatogenesis, as single-cell sequencing can not only identify more accurately which populations are missing but also assess transcriptional changes in the differentiation stages leading up to a defect. Pseudotime analysis, coupled with the comparison of mice with different genotypes, would enable the detection of transcriptomic changes affecting protein coding genes, regulatory RNAs and transposable elements at different developmental stages leading up to a spermatogenic block. This could lead to vastly superior results compared to bulk tissue analyses, especially when considering the possibility to perform network inference in this high dimensional dataset.

## Methods

### Animals

Eight-week-old male C57BL/6J mice obtained from Charles River were used in this study. After sacrificing the animals, testis tissue was harvested and processed as detailed below. All experiments on tissue obtained from animals were carried out in compliance with the relevant guidelines and regulations. As no experiments were carried out on live animals, no approval was required. The project was reported to the Friedrich-Alexander-University Erlangen-Nürnberg and the city of Erlangen (reference TS-04/12).

### Tissue preparation

Tissues were prepared as previously described^29^. Testes of two 8-week-old C57BL/6J littermates (Charles River) were isolated. The tunica albuginea was removed using forceps, and 10 mg of tubules (corresponding to approximately 1/10^th^ of total testis weight) was minced in 200 µl of digestion medium (1 mg/ml each of collagenase/dispase, hyaluronidase, and DNAse I in DMEM/F12) using McPherson-Vannas microscissors. After mincing, 800 µl of digestion medium was added, and the tissue was incubated at 37 °C with slow continuous rotation for 20 min. Every 5 mins, the tissue was triturated using wide-bore pipette tips. The cells were then filtered through a 40-µm cell strainer and centrifuged for 5 min at 400 g and 4 °C. The pellet was then resuspended in 1 ml of PBS, and cell density was assessed by Trypan blue staining and counting using a Neubauer improved counting chamber.

### Single-cell RNA sequencing

Approximately 1250 cells from each mouse were subjected to 10x Chromium Single Cell 3′ Solution v3 library preparation according to the manufacturer’s instructions.

Libraries were sequenced on an Illumina HiSeq 2500 sequencer to a depth of 200 M reads each.

### Reference genome generation

To capture transposons as well as protein-coding genes and non-coding RNAs, a repeat-masked.fasta file (GRCm38/mm10) was obtained from ENSEMBL. The sequences of transposable elements in the mouse were downloaded from RepBase, concatenated and appended to the genome.fasta file as a single sequence. In a similar fashion, the positions within the transposon.fasta sequence were converted to comply with GTF/GFF2 file format specifications and added to the ENSEMBL reference genome annotation file. The reference was then generated using the cellranger mkref command.

### Statistical analysis of single-cell RNA sequencing

QC statistics for single-cell sequencing are listed in Supplementary data Table S6.

Primary analysis of.bcl files output by the short-read sequencer was performed using bcl2fastq 2.17 (Illumina) and cellranger 1.3.1 (10X Genomics). After primary analysis, QC filtering was performed using a variety of tools (scater, scrat, Seurat). As none of these led to the exclusion of more than 5 cells even at stringent settings, no further filtering of cells was performed.

Pseudotime analysis was performed using the scater^30^, monocle^31–33^ and scrat^34–36^ packages for R.

RNA half-life was calculated by applying a LOESS regression to total counts per cell for each sex chromosome and the total of all autosomes divided by the respective mean count per cell. The resulting mean-centered values could thus be compared between chromosomes without absolute expression levels influencing the analysis. The decay was then calculated by obtaining the slope of the regression curve at the point of steepest descent (for autosomes, pseudotimes were limited to larger than 0.98 to exclude the post-meiotic drop in RNA levels). This slope was then divided by the smoothed estimate at the point of steepest descent for the respective chromosome, yielding decay in terms of total chromosomal RNA per unit of pseudotime. As *Tnp1* expression coincides with this decay, its transcript levels were used to estimate real time. TNP1 is expressed in tubules of stages IX–VIII but not IX and X^37^. The total duration of these stages of the epithelial cycle has been estimated at 180.6 hours^38^, giving 200.67 h per 0.01 units of pseudotime. The half-life followed as (0.5/slope) * (200.67/0.01).

### Definition of cell populations through the expression of marker genes

Through thorough literature review, a list of stage markers of spermatogenesis was compiled, containing 233 published markers identified through the detection of RNA or protein. Of these genes, 224 were annotated in the present dataset (Supplementary Table S2). To identify which cluster corresponds to which cell population, marker expression was plotted along the pseudotime axis (Supplementary Fig. S3) and against the different clusters (Supplementary Fig. S4). To include only genes with relevant expression levels in our dataset, genes with a mean expression of 0.1 over all cells that are expressed in at least 3 cells were included, resulting in 214 markers. To define the cell populations, we selected a subset of markers specific for each cluster (Fig. 1C).

### Data availability

Sequencing data for this study have been uploaded to GEO (Accession number GSE104556).

## Electronic supplementary material

Supplementary figures

Supplementary data table 1

Supplementary data table 2

Supplementary data table 3

Supplementary data table 4

Supplementary data table 5

Supplementary data table 6
