# Supplementary material for: Characterization of germ cell differentiation in the male mouse through single-cell RNA sequencing
Source: Sci Rep. 2018 Apr 25;8:6521. doi: 10.1038/s41598-018-24725-0 (PMC5916943; doi:10.1038/s41598-018-24725-0)
Supplement: Supplementary file 1 — Supplementary figures [file 41598_2018_24725_MOESM1_ESM.pdf]

Characterization of germ cell differentiation in the male mouse through single-cell RNA sequencing

Authors: S. Lukassen[1*], E. Bosch[1], A. B. Ekici[1], A. Winterpacht[1].

Supplementary figures

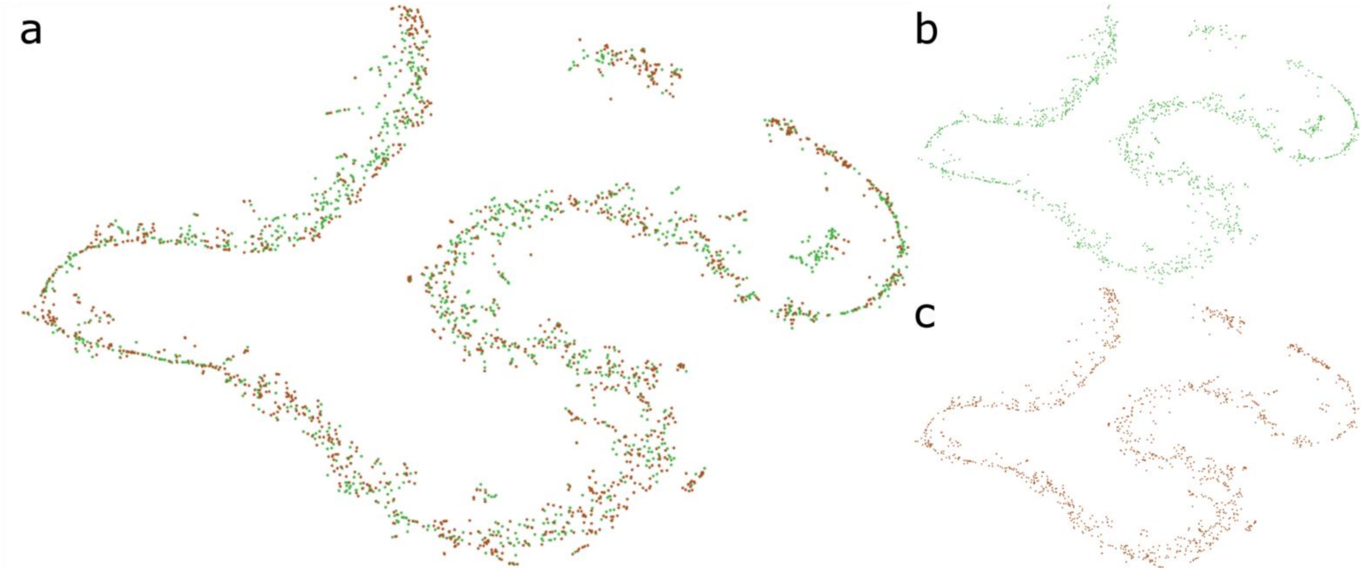

Supplementary Fig. S1. t-SNE projection of the single-cell expression profiles. a) Overlay of mouse 1 (green) and mouse 2 (brown). b) and c) t-SNE of mouse 1 (b) and mouse 2 (c) expression profiles.

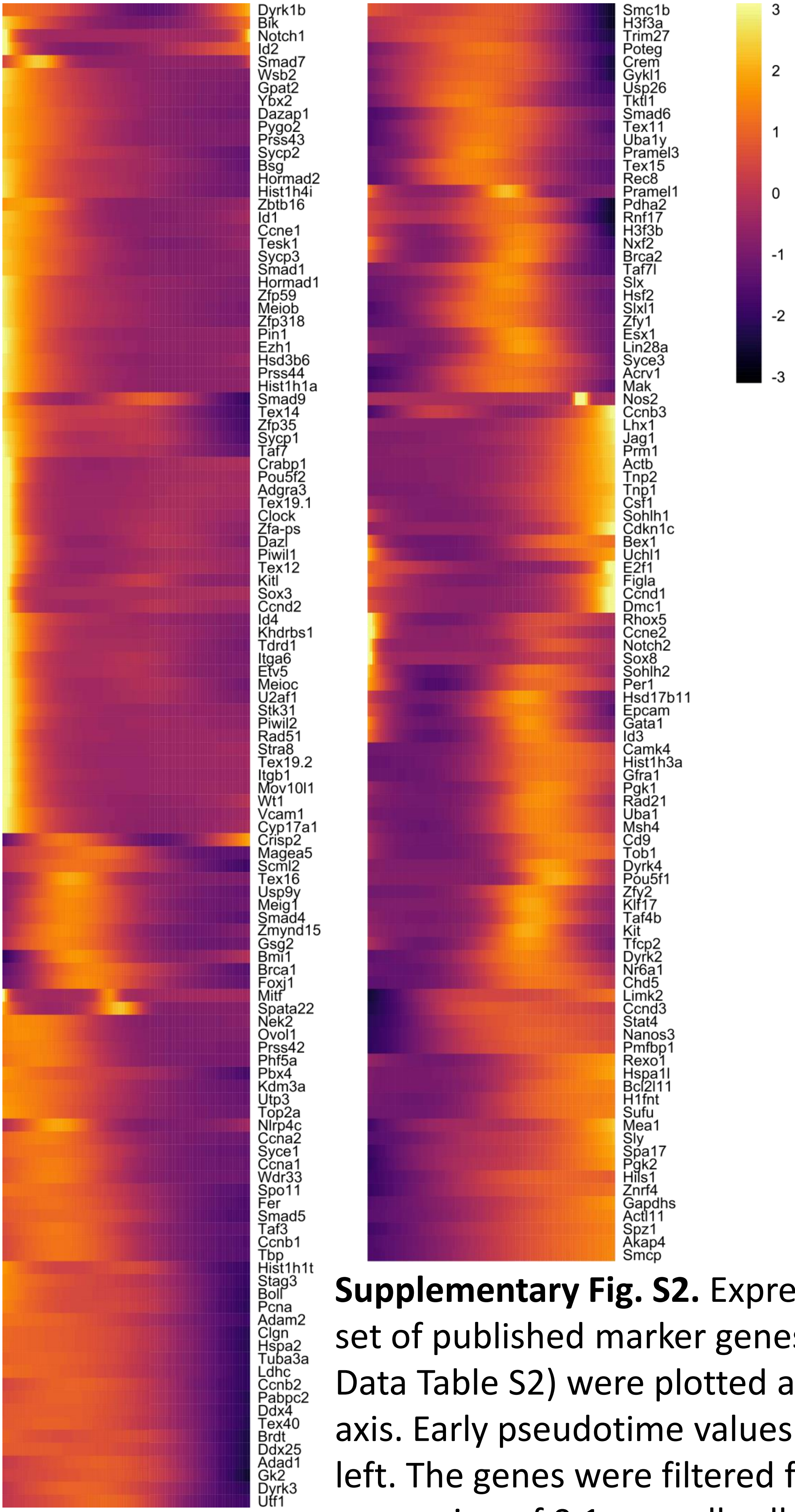

Supplementary Fig. S2. Expression values of a set of published marker genes (Supplementary Data Table S2) were plotted against pseudotime axis. Early pseudotime values are located to the left. The genes were filtered for mean expression of 0.1 over all cells and expression in at least 3 cells in the entire dataset to include markers for rare cell populations

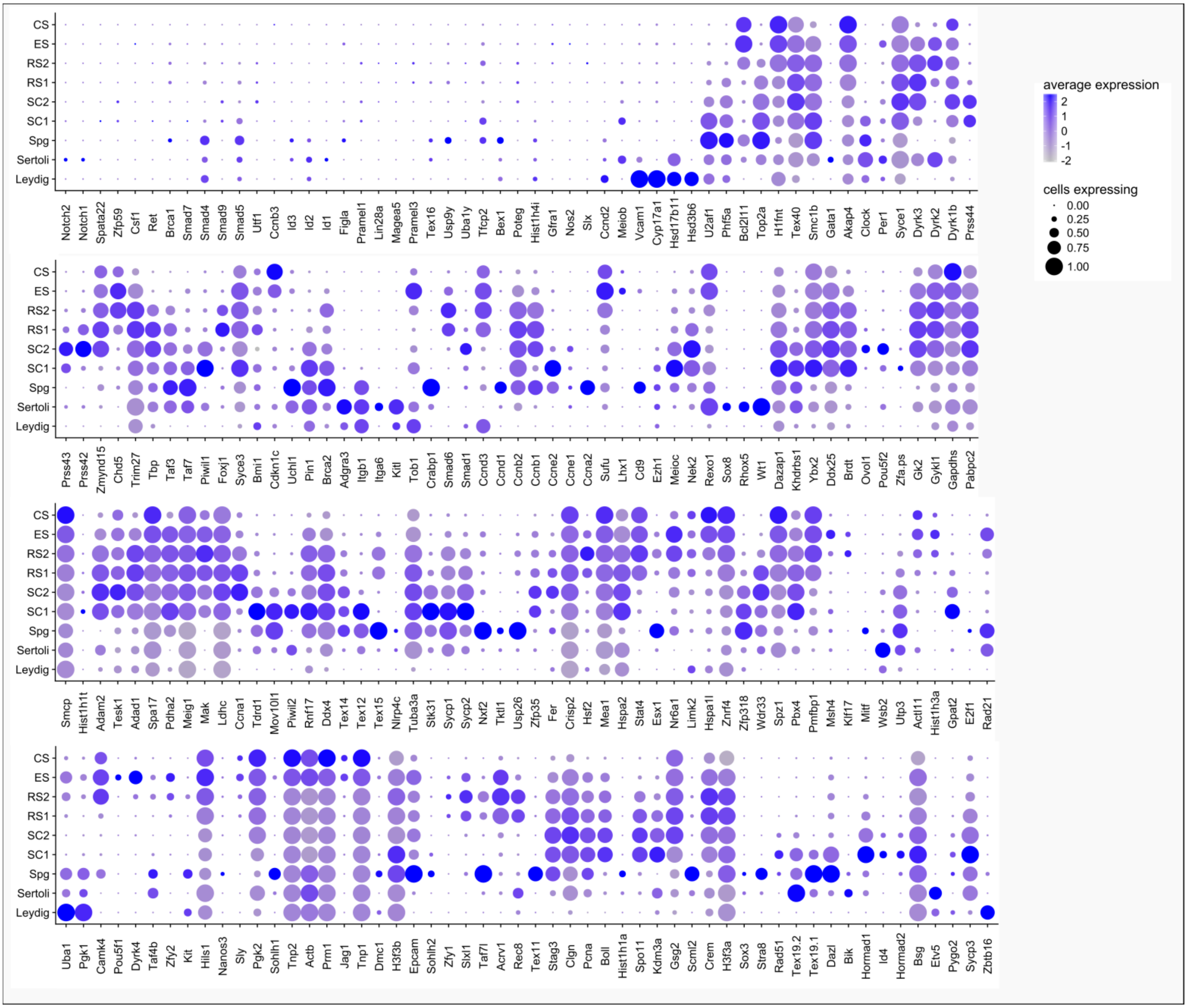

Supplementary Fig. S3. Expression values of a set of published marker genes (Supplementary Data Table S2) were plotted against the different clusters identified through K-Means clustering (K=9). The genes were filtered for mean expression of 0.1 over all cells and expression in at least 3 cells in the entire dataset to include markers for rare cell populations.

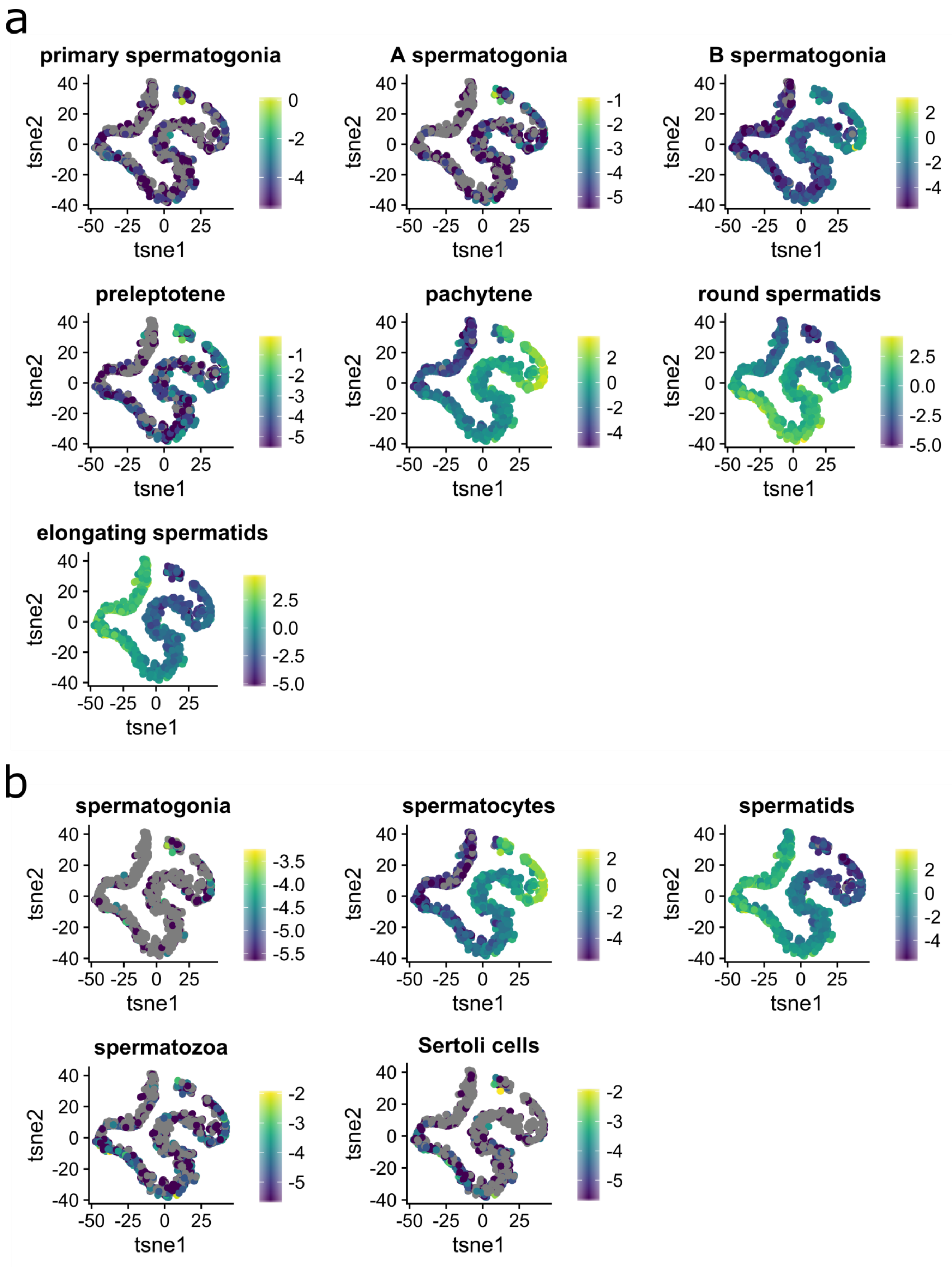

Supplementary Fig. S4. The mean expression of the top 50 marker genes for each cluster identified by a) Gan *et al.* and b) Soumillon *et al.* was mapped onto the t-SNE dimension reduction.

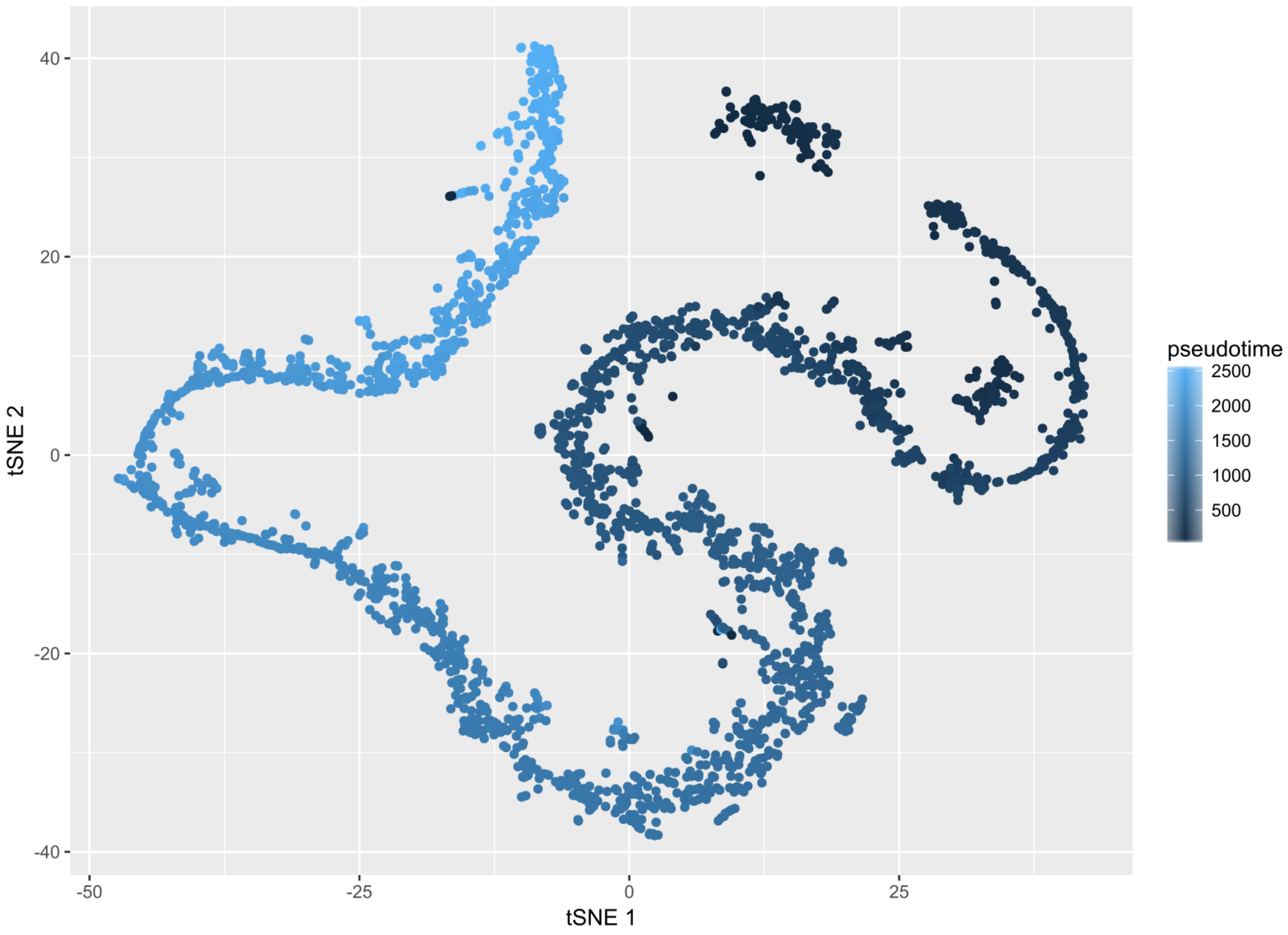

Supplementary Fig. S5. Mapping of the pseudotime rank to the t-SNE projection. Lighter colors indicate later pseudotime.

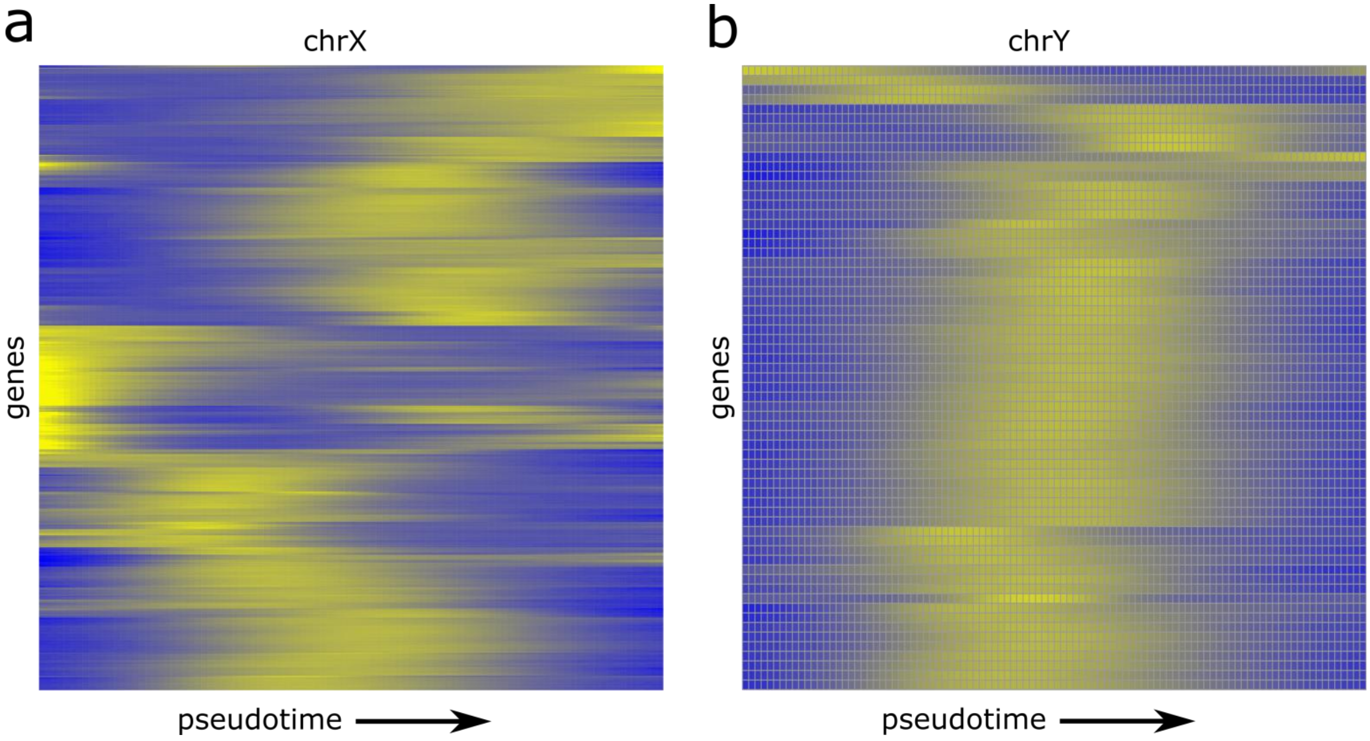

Supplementary Fig. S6. Expression of genes on the sex chromosomes along pseudotime. (a) X-Chromosome. (b) Y-Chromosome.
