# Supplementary material for: Characterization of germ cell differentiation in the male mouse through single-cell RNA sequencing
Source: Sci Rep. 2018 Apr 25;8:6521. doi: 10.1038/s41598-018-24725-0 (PMC5916943; doi:10.1038/s41598-018-24725-0)
Supplement: Supplementary file 2 — Supplementary data table 1 [file 41598_2018_24725_MOESM2_ESM.pdf]

EnsemblID	GeneName	Cluster 1 Average	Cluster 1 Log2 Fold Change	Cluster 1 P-Value	Cluster 2 Average	Cluster 2 Log2 Fold Change	Cluster 2 P-Value	Cluster 3 Average	Cluster 3 Log2 Fold Change	Cluster 3 P-Value	Cluster 4 Average	Cluster 4 Log2 Fold Change	Cluster 4 P-Value	Cluster 5 Average	Cluster 5 Log2 Fold Change	Cluster 5 P-Value	Cluster 6 Average	Cluster 6 Log2 Fold Change	Cluster 6 P-Value	Cluster 7 Average	Cluster 7 Log2 Fold Change	Cluster 7 P-Value	Cluster 8 Average	Cluster 8 Log2 Fold Change	Cluster 8 P-Value	Cluster 9 Average	Cluster 9 Log2 Fold Change	Cluster 9 P-Value	Cluster 10 Average	Cluster 10 Log2 Fold Change	Cluster 10 P-Value	Cluster 11 Average	Cluster 11 Log2 Fold Change	Cluster 11 P-Value
ENSMUSG00000020642	Rnf144a	1.55	4.55	3.07E-30	0.02	-2.94	2.21E-03	0.03	-2.74	3.77E-04	0.01	-4.16	4.10E-13	0.21	0.23	9.65E-01	0.04	-2.18	1.05E-01	0.03	-2.5	2.26E-02	0.07	-1.3	1.90E-01	0.02	-2.83	5.60E-02	0.02	-2.9	2.83E-02	0.43	1.36	2.33E-02
ENSMUSG00000052099	Prss51	7	4.26	6.72E-14	0.11	-3.09	1.85E-04	0.25	-1.96	2.78E-03	0.07	-4.07	1.55E-17	1.27	0.55	4.89E-01	0.08	-3.55	1.42E-03	0.07	-3.78	2.27E-04	0.11	-3.1	2.44E-04	0.15	-2.6	3.84E-02	0.07	-3.76	1.97E-03	2.79	1.88	1.93E-04
ENSMUSG00000021966	Prss52	10.3	3.89	6.72E-14	0.2	-2.94	2.91E-04	0.52	-1.6	1.61E-02	0.12	-3.91	4.72E-17	0.99	-0.58	1.00E+00	0.13	-3.49	1.51E-03	0.23	-2.77	3.31E-03	0.21	-2.86	5.75E-04	0.24	-2.7	3.05E-02	0.19	-2.97	8.72E-03	6.24	2.47	1.99E-07
ENSMUSG00000045294	Insig1	1.67	3.83	1.66E-21	0.06	-1.99	3.24E-02	0.06	-2.12	3.03E-03	0.03	-3.05	4.06E-10	0.48	1.07	1.21E-01	0.05	-2.16	8.91E-02	0.06	-2.05	4.95E-02	0.1	-1.23	1.90E-01	0.04	-2.64	5.79E-02	0.03	-2.87	2.11E-02	0.65	1.61	3.45E-03
ENSMUSG00000032666	1700025G04Rik	3.17	3.73	1.22E-21	0.09	-2.54	2.40E-03	0.1	-2.38	3.94E-04	0.04	-3.98	5.34E-16	1.34	1.66	5.17E-03	0.09	-2.46	2.95E-02	0.09	-2.38	1.29E-02	0.11	-2.17	9.52E-03	0.09	-2.45	5.88E-02	0.08	-2.51	2.83E-02	1.38	1.76	6.76E-04
ENSMUSG00000071015	Gm136	9.73	3.73	6.72E-14	0.15	-3.33	3.40E-05	0.43	-1.88	3.06E-03	0.09	-4.37	1.24E-20	4.18	1.68	3.50E-03	0.12	-3.58	8.92E-04	0.16	-3.29	4.78E-04	0.1	-3.92	4.48E-06	0.22	-2.78	2.08E-02	0.19	-2.97	6.76E-03	4.92	2.02	4.48E-05
ENSMUSG00000097311	Gm26871	2.55	3.7	2.28E-21	0.08	-2.4	4.31E-03	0.07	-2.5	2.39E-04	0.02	-4.39	2.53E-17	1.08	1.65	5.67E-03	0.03	-3.42	2.70E-03	0.04	-3.28	1.20E-03	0.05	-2.83	9.92E-04	0.06	-2.56	4.76E-02	0.02	-4.23	1.25E-02	1.38	2.13	1.53E-05
ENSMUSG00000022965	Ifngr2	2.02	3.63	2.42E-19	0.04	-2.99	8.07E-04	0.06	-2.42	6.49E-04	0.1	-1.84	3.65E-05	0.2	-0.65	1.00E+00	0.05	-2.52	3.60E-02	0.04	-2.96	4.00E-03	0.07	-2.07	1.82E-02	0.06	-2.46	7.72E-02	0.07	-2.14	9.18E-02	1.3	2.43	5.82E-07
ENSMUSG00000051936	Prss58	13.72	3.62	6.72E-14	0.38	-2.6	7.89E-04	0.54	-2.11	7.78E-04	0.22	-3.59	2.83E-16	6.66	1.83	2.07E-03	0.33	-2.75	7.54E-03	0.33	-2.77	2.23E-03	0.27	-3.03	1.53E-04	0.36	-2.61	2.76E-02	0.26	-3.07	4.73E-03	6.5	1.84	2.92E-04
ENSMUSG00000013668	4933402N03Rik	3.31	3.58	2.38E-20	0.09	-2.67	1.08E-03	0.13	-2.06	1.64E-03	0.02	-4.74	4.21E-20	1.51	1.7	3.74E-03	0.07	-2.95	6.77E-03	0.05	-3.36	6.82E-04	0.04	-3.76	2.39E-05	0.06	-3.1	1.46E-02	0.04	-3.52	3.61E-03	2.01	2.27	1.63E-06
ENSMUSG00000040714	Klc3	2.5	3.56	2.07E-19	0.05	-2.95	5.55E-04	0.08	-2.43	3.54E-04	0.03	-4.02	9.53E-16	0.84	1.18	6.15E-02	0.06	-2.64	1.96E-02	0.07	-2.59	8.14E-03	0.07	-2.58	2.53E-03	0.11	-1.91	1.96E-01	0.05	-2.99	1.17E-02	1.62	2.4	4.50E-07
ENSMUSG00000072919	Noxred1	4.26	3.51	6.72E-14	0.09	-2.97	2.68E-04	0.15	-2.32	3.62E-04	0.04	-4.26	1.87E-18	2.4	2.02	4.89E-04	0.07	-3.34	2.14E-03	0.05	-3.66	3.00E-04	0.12	-2.51	2.15E-03	0.11	-2.72	2.84E-02	0.08	-3.2	5.29E-03	2.36	2.06	2.26E-05
ENSMUSG00000055177	Cstl1	21.69	3.49	6.72E-14	0.62	-2.62	5.98E-04	0.83	-2.2	3.81E-04	0.29	-3.94	5.92E-19	16.26	2.52	1.46E-06	0.48	-2.93	3.79E-03	0.62	-2.59	3.56E-03	0.42	-3.17	6.94E-05	0.57	-2.71	2.08E-02	0.44	-3.07	4.47E-03	8.35	1.4	1.11E-02
ENSMUSG00000100548	Gm29585	1.6	3.45	1.53E-17	0.06	-2.11	1.82E-02	0.08	-1.88	7.06E-03	0.02	-4.02	1.46E-14	1.16	2.45	1.14E-05	0.06	-2.05	9.90E-02	0.05	-2.26	2.48E-02	0.01	-4.09	3.47E-05	0.05	-2.46	7.15E-02	0.05	-2.28	6.21E-02	0.6	1.36	1.63E-02
ENSMUSG00000082791	Gm4875	1.5	3.43	4.59E-17	0.03	-2.9	1.24E-03	0.05	-2.45	6.05E-04	0.01	-4.43	9.67E-16	1.47	3.02	2.69E-09	0.06	-2.07	1.02E-01	0.05	-2.18	3.24E-02	0.03	-2.83	1.71E-03	0.03	-2.84	3.65E-02	0.03	-3.1	1.31E-02	0.4	0.78	2.42E-01
ENSMUSG00000037737	Actrt3	7.99	3.43	6.72E-14	0.22	-2.66	1.07E-03	0.79	-0.8	3.46E-01	0.1	-4.03	1.20E-17	0.65	-1.05	6.34E-01	0.14	-3.24	2.79E-03	0.21	-2.72	3.83E-03	0.18	-2.92	4.61E-04	0.24	-2.55	4.24E-02	0.23	-2.56	2.33E-02	6.7	2.83	3.35E-10
ENSMUSG00000087250	Usp46os1	3.78	3.43	6.72E-14	0.1	-2.7	9.01E-04	0.58	-0.1	1.00E+00	0.05	-3.91	1.69E-16	0.56	-0.14	1.00E+00	0.09	-2.87	7.87E-03	0.08	-3.04	1.52E-03	0.11	-2.58	1.69E-03	0.07	-3.18	1.26E-02	0.08	-3	8.35E-03	2.68	2.48	6.72E-08
ENSMUSG00000015962	1700016C15Rik	13.5	3.42	6.72E-14	0.28	-3.13	6.98E-05	0.76	-1.66	8.77E-03	0.12	-4.52	4.20E-22	4.55	1.11	6.86E-02	0.31	-2.91	4.54E-03	0.24	-3.29	4.35E-04	0.24	-3.32	4.43E-05	0.3	-2.97	1.35E-02	0.3	-2.97	6.26E-03	10.45	2.65	2.73E-09
ENSMUSG00000050087	Cby3	4.91	3.41	6.72E-14	0.16	-2.46	2.10E-03	0.16	-2.43	1.74E-04	0.06	-4.04	9.22E-18	5.24	3.17	1.96E-11	0.13	-2.7	1.16E-02	0.09	-3.3	6.16E-04	0.13	-2.68	1.00E-03	0.11	-2.92	1.79E-02	0.09	-3.17	5.08E-03	1.08	0.45	5.39E-01
ENSMUSG00000017720	Trp53tg5	17.58	3.41	6.72E-14	0.36	-3.13	8.06E-05	1.69	-0.87	2.74E-01	0.17	-4.43	2.46E-21	2.41	-0.29	1.00E+00	0.33	-3.2	2.23E-03	0.41	-2.94	1.42E-03	0.37	-3.06	1.66E-04	0.41	-2.91	1.65E-02	0.37	-3.06	5.51E-03	15.24	2.88	4.86E-11
ENSMUSG00000060407	Cyp2a12	3.16	3.11	1.46E-14	0.09	-2.86	5.12E-04	0.08	-2.93	1.58E-05	0.04	-4.09	3.79E-17	4.3	3.46	3.25E-13	0.12	-2.33	3.87E-02	0.11	-2.48	8.70E-03	0.05	-3.45	7.42E-05	0.07	-3.01	1.79E-02	0.07	-3.07	7.75E-03	0.92	0.7	2.82E-01
ENSMUSG00000005628	Tmod4	0.7	3.06	5.99E-12	0.01	-3.16	1.98E-03	0.03	-2.14	6.15E-03	0.01	-3.69	1.06E-10	1.01	3.54	2.93E-12	0.03	-2.08	1.44E-01	0.04	-1.87	1.10E-01	0.04	-1.63	9.63E-02	0.04	-1.61	4.67E-01	0.01	-3.02	2.74E-02	0.11	-0.25	1.00E+00
ENSMUSG00000063089	Klk1b8	12.9	2.84	1.72E-12	0.43	-2.77	3.26E-04	0.65	-2.17	5.16E-04	0.22	-3.91	1.67E-18	19.07	3.4	3.25E-13	0.38	-2.88	4.72E-03	0.5	-2.51	4.80E-03	0.31	-3.21	6.36E-05	0.45	-2.66	2.38E-02	0.48	-2.56	1.66E-02	5.58	1.17	4.66E-02
ENSMUSG00000085491	4930527E20Rik	2.22	2.79	1.78E-11	0.07	-2.84	6.57E-04	0.18	-1.5	2.76E-02	0.03	-4.23	2.65E-17	3.45	3.46	3.25E-13	0.08	-2.54	2.26E-02	0.04	-3.45	6.45E-04	0.07	-2.68	1.44E-03	0.05	-3.19	1.39E-02	0.06	-3.03	8.87E-03	0.98	1.16	4.06E-02
ENSMUSG00000011350	Gm5893	3.85	2.77	1.92E-11	0.11	-3.06	1.56E-04	0.17	-2.4	2.03E-04	0.05	-4.24	7.26E-19	6.07	3.47	3.25E-13	0.1	-3.13	3.26E-03	0.1	-3.14	1.01E-03	0.17	-2.38	3.24E-03	0.08	-3.34	9.31E-03	0.1	-3.06	6.40E-03	1.99	1.41	7.93E-03
ENSMUSG00000069118	1700008P02Rik	4.22	2.75	2.50E-11	0.17	-2.54	1.42E-03	0.23	-2.08	1.18E-03	0.08	-3.84	7.99E-17	6.95	3.56	3.25E-13	0.18	-2.41	2.53E-02	0.15	-2.65	3.97E-03	0.1	-3.3	7.81E-05	0.14	-2.72	2.57E-02	0.2	-2.22	4.53E-02	1.61	0.9	1.30E-01
ENSMUSG00000104872	1700008B11Rik	2.56	2.73	4.10E-11	0.1	-2.63	1.32E-03	0.11	-2.47	1.87E-04	0.07	-3.39	1.55E-13	4.03	3.43	3.25E-13	0.06	-3.15	3.86E-03	0.11	-2.41	1.05E-02	0.12	-2.21	7.02E-03	0.12	-2.25	8.36E-02	0.09	-2.69	1.77E-02	1.16	1.17	3.62E-02
ENSMUSG00000027562	Car2	13.72	2.69	5.43E-11	0.73	-2.18	5.11E-03	0.82	-2.03	1.17E-03	0.39	-3.29	1.33E-14	22.21	3.45	3.25E-13	0.52	-2.64	1.04E-02	0.69	-2.23	1.26E-02	0.59	-2.45	1.74E-03	0.65	-2.31	5.59E-02	0.75	-2.11	5.28E-02	5.22	0.85	2.04E-01
ENSMUSG00000038015	Prm2	454.43	2.64	9.42E-11	19.76	-2.5	3.83E-04	26.4	-2.1	2.91E-04	9.71	-3.74	1.24E-18	758.93	3.48	3.25E-13	18.22	-2.59	4.02E-03	19.95	-2.47	2.33E-03	14.72	-2.91	5.38E-05	18.57	-2.56	1.51E-02	21.15	-2.37	1.26E-02	219.36	1.21	3.53E-02
ENSMUSG00000051036	Ttc24	10.36	2.6	2.92E-10	0.45	-2.51	1.14E-03	0.71	-1.88	2.62E-03	0.24	-3.62	1.27E-16	17.58	3.47	3.25E-13	0.33	-2.91	4.35E-03	0.5	-2.35	8.41E-03	0.36	-2.81	3.66E-04	0.48	-2.39	4.47E-02	0.45	-2.49	2.01E-02	5.15	1.23	3.30E-02
ENSMUSG00000026473	Glul	13.67	2.59	4.13E-10	0.59	-2.53	1.03E-03	1.23	-1.47	2.17E-02	0.27	-3.86	2.81E-18	24.14	3.55	3.25E-13	0.49	-2.78	6.55E-03	0.53	-2.67	2.93E-03	0.64	-2.39	2.21E-03	0.45	-2.89	1.51E-02	0.63	-2.42	2.36E-02	6.08	1.03	9.54E-02
ENSMUSG000001019	1700027A15Rik	79.1	2.59	2.99E-	4.06	-2.29	1.18E-	5.83	-1.78	2.13E-	2.15	-3.42	2.37E-	133.2	3.44	3.25E-	3.86	-2.33	2.36E-	3.85	-2.35	3.52E-	3.41	-2.53	1.06E-	4.02	-2.28	5.55E-	4.36	-2.16	2.30E-	35.62	1.05	8.28E-

68				10			03			03			16	5		13			02			03			03			02			02			02
ENSMUSG00000031085	Gm498	5.12	2.51	3.11E-09	0.24	-2.46	1.84E-03	0.3	-2.17	6.51E-04	0.15	-3.39	1.56E-14	9.59	3.6	3.25E-13	0.26	-2.29	3.50E-02	0.26	-2.34	1.02E-02	0.22	-2.59	1.28E-03	0.3	-2.1	1.03E-01	0.3	-2.13	5.68E-02	2.3	0.99	8.68E-02
ENSMUSG00000032566	1700080E11Rik	11.63	2.5	2.98E-09	0.68	-2.16	5.90E-03	0.74	-2.06	1.00E-03	0.29	-3.58	2.85E-16	20.76	3.48	3.25E-13	0.63	-2.23	3.69E-02	0.65	-2.2	1.41E-02	0.46	-2.71	5.95E-04	0.53	-2.47	3.79E-02	0.74	-2	7.19E-02	6.1	1.23	3.26E-02
ENSMUSG00000027482	Bpifa3	6.39	2.48	5.06E-09	0.32	-2.41	2.13E-03	0.43	-1.99	1.73E-03	0.13	-3.86	1.41E-17	11.88	3.55	3.25E-13	0.27	-2.6	1.32E-02	0.41	-2.01	2.89E-02	0.33	-2.33	3.32E-03	0.28	-2.56	3.34E-02	0.3	-2.43	2.50E-02	3.37	1.23	2.33E-02
ENSMUSG00000051437	Ubqlnl	5.94	2.46	6.84E-09	0.28	-2.51	1.33E-03	0.69	-1.18	8.53E-02	0.12	-3.92	6.23E-18	10.95	3.51	3.25E-13	0.27	-2.51	1.68E-02	0.21	-2.88	1.71E-03	0.2	-2.98	2.20E-04	0.22	-2.82	1.89E-02	0.2	-2.92	7.69E-03	3.41	1.36	1.06E-02
ENSMUSG00000050721	Plekho2	1.55	2.45	2.56E-08	0.08	-2.34	6.80E-03	0.11	-1.88	6.12E-03	0.04	-3.46	7.65E-13	2.79	3.45	5.71E-13	0.07	-2.5	3.13E-02	0.11	-1.85	6.94E-02	0.06	-2.72	1.79E-03	0.08	-2.3	9.67E-02	0.07	-2.45	3.95E-02	0.92	1.41	1.14E-02
ENSMUSG00000037681	Esyt3	0.71	2.44	3.43E-07	0.03	-2.7	6.96E-03	0.06	-1.71	2.90E-02	0	-6.28	2.79E-17	0.08	-1.03	7.46E-01	0.02	-2.97	2.70E-02	0.02	-3.29	4.90E-03	0.05	-1.68	8.97E-02	0.02	-3.03	4.89E-02	0.02	-2.83	4.03E-02	1.48	4.12	4.11E-21
ENSMUSG00000071033	Gm10308	0.64	2.37	3.03E-07	0.04	-2.06	3.21E-02	0.08	-1.12	1.60E-01	0.02	-3.45	7.69E-11	1.19	3.45	3.37E-12	0.05	-1.59	2.95E-01	0.04	-2.18	4.44E-02	0.04	-1.94	3.49E-02	0.01	-3.26	2.61E-02	0.05	-1.72	2.52E-01	0.34	1.16	6.32E-02
ENSMUSG00000086898	Itpr3os	1.33	2.37	8.73E-08	0.1	-1.82	4.41E-02	0.11	-1.68	1.48E-02	0.04	-3.51	4.36E-13	2.43	3.39	1.22E-12	0.08	-2.16	7.19E-02	0.13	-1.45	1.92E-01	0.1	-1.87	2.77E-02	0.08	-2.06	1.52E-01	0.09	-1.91	1.28E-01	0.72	1.18	4.00E-02
ENSMUSG00000041165	Spem1	10.83	2.19	5.09E-07	0.65	-2.33	2.55E-03	2.08	-0.6	5.43E-01	0.3	-3.66	6.36E-17	21.8	3.42	3.25E-13	0.56	-2.5	1.56E-02	0.81	-2	2.75E-02	0.56	-2.51	1.31E-03	0.67	-2.25	6.33E-02	0.84	-1.93	8.49E-02	6.72	1.27	2.56E-02
ENSMUSG00000030214	Plbd1	0.82	1.99	2.73E-05	0.05	-2.49	7.13E-03	0.27	0.13	9.79E-01	0.01	-4.8	2.37E-16	0.08	-1.68	2.76E-01	0.04	-2.65	3.44E-02	0.01	-4.14	4.78E-04	0.01	-4.05	7.16E-05	0.04	-2.55	7.68E-02	0.02	-3.63	6.51E-03	2.12	4.09	1.52E-22
ENSMUSG00000109049	Gm44599	1.37	1.89	5.56E-05	0.06	-2.98	6.86E-04	0.18	-1.38	5.78E-02	0.04	-3.86	1.62E-14	0.13	-1.82	2.01E-01	0.05	-3.19	6.64E-03	0.06	-2.96	3.65E-03	0.03	-3.88	4.73E-05	0.09	-2.38	8.80E-02	0.06	-2.87	1.87E-02	4.28	4.54	4.32E-30
ENSMUSG00000028328	Tmod1	0.86	1.65	5.58E-04	0.05	-2.74	2.02E-03	0.32	0.01	1.00E+00	0.03	-3.59	6.64E-13	0.16	-1.01	7.06E-01	0.05	-2.57	3.22E-02	0.03	-3.31	1.77E-03	0.05	-2.68	2.70E-03	0.06	-2.51	6.72E-02	0.03	-3.29	8.66E-03	2.76	4.18	5.95E-25
ENSMUSG00000013083	2200002J24Rik	1.93	1.43	2.62E-03	0.13	-2.68	1.15E-03	0.81	0	1.00E+00	0.07	-3.82	6.84E-16	0.44	-0.89	7.94E-01	0.07	-3.5	1.73E-03	0.18	-2.25	1.82E-02	0.07	-3.47	6.73E-05	0.22	-1.93	1.81E-01	0.15	-2.49	2.89E-02	7.09	4.24	4.54E-14
ENSMUSG00000107988	Gm44006	1.06	1.19	1.61E-02	0.08	-2.72	1.17E-03	0.76	0.66	2.09E-01	0.04	-4.05	2.04E-16	0.29	-0.86	8.30E-01	0.05	-3.26	3.67E-03	0.2	-1.42	1.95E-01	0.04	-3.74	3.48E-05	0.14	-1.88	2.02E-01	0.09	-2.57	2.53E-02	4.17	4	1.18E-23
ENSMUSG00000026237	Nmur1	0.33	1.05	6.28E-02	0.02	-2.86	3.47E-03	0.18	0.09	1.00E+00	0.02	-3.53	8.57E-11	0.1	-0.82	9.35E-01	0.03	-2.27	9.37E-02	0.03	-2.58	2.10E-02	0.02	-2.91	2.92E-03	0.06	-1.61	4.43E-01	0.01	-3.82	7.69E-03	1.61	4.45	3.94E-26
ENSMUSG00000021003	Galc	0.34	1.05	5.55E-02	0.06	-1.63	1.23E-01	0.23	0.39	5.93E-01	0.03	-3.02	3.13E-09	0.06	-1.56	3.28E-01	0.01	-3.33	9.73E-03	0.06	-1.53	2.09E-01	0.05	-1.72	6.47E-02	0.05	-1.91	2.68E-01	0.04	-2.12	1.18E-01	1.47	4.03	2.39E-21
ENSMUSG00000038534	Osbpl7	0.24	0.88	1.87E-01	0.02	-2.99	1.05E-02	0.22	0.76	2.29E-01	0.01	-3.79	2.18E-09	0.01	-2.99	8.45E-02	0.01	-3	6.08E-02	0.01	-3.73	6.97E-03	0	-4.22	8.18E-04	0.04	-1.71	5.37E-01	0.02	-2.8	9.04E-02	1.33	4.47	4.11E-21
ENSMUSG00000068686	Cd59b	0.45	0.77	1.89E-01	0.04	-2.96	1.37E-03	0.5	0.98	4.97E-02	0.02	-4.35	4.11E-15	0.08	-1.83	2.18E-01	0.04	-2.64	3.35E-02	0.03	-3.28	2.63E-03	0.03	-3.02	1.37E-03	0.04	-2.85	4.29E-02	0.02	-3.51	7.73E-03	2.53	4.34	5.43E-26
ENSMUSG00000085240	Gm12119	0.22	0.6	3.98E-01	0.01	-3.38	1.52E-03	0.21	0.53	4.43E-01	0.01	-4.28	4.12E-12	0.04	-1.87	2.76E-01	0.02	-2.79	5.16E-02	0.01	-3.85	2.64E-03	0.02	-2.75	7.42E-03	0.03	-2.16	2.41E-01	0.02	-2.92	4.17E-02	1.55	4.72	1.26E-27
ENSMUSG00000041255	Tmco5b	0.82	0.56	3.52E-01	0.06	-3.35	1.32E-04	0.95	0.84	9.12E-02	0.03	-4.33	2.14E-17	0.3	-0.97	7.44E-01	0.06	-3.31	3.88E-03	0.08	-2.86	3.89E-03	0.05	-3.65	6.38E-05	0.1	-2.54	5.67E-02	0.06	-3.23	7.42E-03	5.36	4.45	1.56E-29
ENSMUSG00000049491	Slc36a3	0.58	0.45	4.95E-01	0.06	-2.87	1.28E-03	0.77	0.97	4.80E-02	0.02	-4.78	5.10E-18	0.11	-2	1.57E-01	0.04	-3.29	5.97E-03	0.04	-3.61	8.86E-04	0.05	-3.13	6.30E-04	0.11	-2.01	1.96E-01	0.07	-2.64	3.10E-02	4.06	4.45	3.41E-28
ENSMUSG00000022685	Parn	0.3	0.34	6.88E-01	0.06	-2.15	2.19E-02	0.45	1.06	3.00E-02	0.02	-4.02	1.05E-13	0.1	-1.22	5.55E-01	0.03	-2.94	1.64E-02	0.07	-1.8	1.02E-01	0.05	-2.15	1.80E-02	0.11	-1.19	6.96E-01	0.07	-1.77	2.19E-01	1.96	4	1.74E-21
ENSMUSG00000021890	Eaf1	0.26	0.31	7.23E-01	0.05	-2.04	2.71E-02	0.41	1.15	1.40E-02	0.02	-3.72	1.32E-12	0.11	-0.94	7.51E-01	0.04	-2.24	7.74E-02	0.04	-2.41	2.05E-02	0.1	-1.16	2.24E-01	0.05	-2.16	1.53E-01	0.02	-3.11	1.48E-02	1.71	3.98	1.15E-21
ENSMUSG00000108924	4933436H12Rik	0.12	-0.03	1.00E+00	0.02	-2.6	1.17E-02	0.3	1.55	9.67E-04	0.01	-4.21	8.24E-12	0.04	-1.58	3.69E-01	0.01	-2.83	4.33E-02	0.01	-3.57	4.00E-03	0.01	-3.05	3.30E-03	0.03	-1.87	3.37E-01	0.01	-3.37	2.07E-02	1.1	4.2	1.24E-21
ENSMUSG00000085940	4930405D11Rik	0.69	-0.3	1.00E+00	0.07	-3.68	2.38E-05	2.35	1.87	3.85E-06	0.04	-4.77	1.78E-20	0.25	-1.76	2.06E-01	0.05	-3.97	7.65E-04	0.12	-2.82	3.62E-03	0.06	-3.87	1.85E-05	0.12	-2.85	2.59E-02	0.06	-3.78	2.26E-03	7	4.08	4.54E-14
ENSMUSG00000015053	Gata2	0.37	-0.3	1.00E+00	0.41	-0.17	1.00E+00	0.91	1.2	4.15E-03	0.01	-5.33	2.95E-23	0.06	-2.92	1.17E-02	0.05	-3.21	2.22E-03	1.05	1.37	9.68E-03	0.01	-5.32	8.21E-08	0.71	0.7	5.64E-01	1.34	1.79	2.26E-03	0.59	0.4	6.02E-01
ENSMUSG00000030345	Dyrk4	0.26	-0.33	1.00E+00	0.02	-3.79	9.96E-05	0.62	1.1	2.41E-02	0.03	-3.91	1.55E-13	0.05	-2.6	5.88E-02	0.04	-2.88	2.07E-02	0.03	-3.52	1.55E-03	0.02	-3.61	2.32E-04	0.1	-1.78	3.32E-01	0.05	-2.74	3.16E-02	3.21	4.63	1.07E-29
ENSMUSG00000011486	Slc25a41	0.25	-0.38	1.00E+00	0.04	-3.06	3.83E-04	1.5	3.14	3.15E-18	0.02	-4.05	1.30E-15	0.06	-2.43	5.25E-02	0.02	-3.6	2.02E-03	0.2	-0.7	8.18E-01	0.02	-3.82	4.06E-05	0.03	-3.24	1.51E-02	0.06	-2.39	4.17E-02	1.41	2.5	7.66E-08
ENSMUSG00000026989	Dapl1	0.16	-0.65	6.87E-01	0.03	-2.9	1.18E-03	1.12	3.08	1.00E-16	0.01	-5.18	3.75E-18	0.1	-1.34	4.30E-01	0.02	-3.2	7.03E-03	0.27	0.18	1.00E+00	0.02	-3.42	2.67E-04	0.09	-1.5	4.51E-01	0.17	-0.56	1.00E+00	0.78	1.89	2.76E-04
ENSMUSG00000016493	Cd46	0.12	-0.68	8.68E-01	0.04	-2.38	4.68E-02	0.16	-0.28	1.00E+00	0.01	-4.03	2.16E-10	0.06	-1.63	4.99E-01	0.02	-3.07	5.63E-02	0.02	-3.12	1.95E-02	0.01	-3.61	2.36E-03	0.05	-1.98	4.26E-01	0.02	-3.2	4.78E-02	2.19	5.5	4.64E-33
ENSMUSG00000099508	1700030L20Rik	0.16	-0.8	4.92E-01	0.04	-2.69	1.47E-03	1.15	2.87	3.68E-15	0.01	-4.43	6.54E-17	0.15	-0.82	8.43E-01	0.01	-3.93	1.15E-03	0.52	1.05	8.75E-02	0.01	-4.56	5.44E-06	0.1	-1.43	4.50E-01	0.28	0.05	1.00E+00	0.61	1.3	1.96E-02
ENSMUSG00000018925	Heatr9	0.21	-0.82	4.77E-01	0.05	-3	4.09E-04	1.6	3.03	5.38E-17	0.02	-4.59	3.65E-18	0.08	-2.08	1.01E-01	0.01	-4.33	5.21E-04	0.39	0.14	1.00E+00	0.03	-3.54	7.96E-05	0.09	-2.04	1.42E-01	0.15	-1.24	4.60E-01	1.4	2.29	1.52E-06
ENSMUSG00000022037	Clu	0.25	-0.87	1.00E+00	0.24	-0.95	1.00E+00	0.13	-1.84	7.14E-01	0.1	-2.47	4.93E-02	0.25	-0.83	1.00E+00	0.12	-1.93	1.00E+00	0.12	-1.89	1.00E+00	3.86	4.59	4.31E-08	0.19	-1.28	1.00E+00	0.15	-1.61	1.00E+00	0.18	-1.31	1.00E+00
ENSMUSG00000106540	4930590L14Rik	0.15	-0.9	4.02E-01	0.02	-3.72	4.02E-05	1.17	2.88	2.65E-15	0.01	-4.92	1.21E-18	0.04	-2.51	4.02E-02	0.03	-3.13	5.60E-03	0.53	1.08	7.73E-02	0.01	-4.58	4.74E-06	0.1	-1.51	3.97E-01	0.18	-0.65	1.00E+00	0.86	1.87	2.18E-04

ENSMUSG00000091296	Gm4181	0.32	-0.93	3.26E-01	1.8	1.93	9.80E-06	0.61	0.03	1.00E+00	0.02	-4.87	1.34E-22	0.3	-1	6.04E-01	0.58	-0.05	1.00E+00	0.87	0.59	4.64E-01	0.02	-4.71	2.47E-07	1.2	1.11	1.46E-01	1.18	1.08	1.08E-01	0.4	-0.61	8.24E-01
ENSMUSG00000026822	Lcn2	0.27	-1.02	8.87E-01	0.33	-0.72	1.00E+00	0.13	-2.14	1.00E+00	0.08	-2.93	6.12E-01	0.33	-0.68	8.23E-01	0.15	-1.86	1.00E+00	0.2	-1.48	1.00E+00	4.75	4.79	5.62E-04	0.12	-2.22	1.00E+00	0.13	-2.08	1.00E+00	0.15	-1.8	1.00E+00
ENSMUSG00000018593	Sparc	0.08	-1.07	1.00E+00	0.13	-0.38	1.00E+00	0.06	-1.53	9.21E-01	0.03	-2.53	6.44E-02	0.1	-0.7	1.00E+00	0.04	-1.92	1.00E+00	0.03	-2.56	1.00E+00	1.55	4.83	3.13E-08	0.02	-2.99	1.00E+00	0.03	-2.56	1.00E+00	0.03	-2.19	1.00E+00
ENSMUSG00000095930	Nim1k	0.09	-1.29	2.25E-01	0.03	-2.94	2.90E-03	0.29	0.56	3.79E-01	0.02	-3.57	5.75E-11	0.02	-3.1	3.47E-02	0.02	-3.21	1.62E-02	0.07	-1.49	2.54E-01	0.08	-1.32	1.94E-01	0.05	-1.98	2.74E-01	0.04	-2.18	1.27E-01	2.12	4.84	8.54E-31
ENSMUSG00000025044	Msr1	0.13	-1.44	8.28E-02	0.07	-2.37	3.39E-03	0.93	1.81	3.51E-06	0.05	-3.11	4.43E-12	0.07	-2.24	6.05E-02	0.11	-1.72	1.54E-01	1.15	2.09	3.24E-05	0.01	-4.49	2.59E-06	0.32	-0.08	1.00E+00	0.61	0.93	2.29E-01	0.51	0.63	3.48E-01
ENSMUSG00000092503	Gm20484	0.14	-1.47	8.49E-02	0.05	-2.99	3.35E-04	1.66	2.95	2.22E-16	0.02	-4.58	1.41E-18	0.06	-2.66	2.83E-02	0.04	-3.24	3.54E-03	0.71	1.02	9.43E-02	0.03	-3.64	4.78E-05	0.07	-2.38	6.43E-02	0.21	-0.91	7.46E-01	1.26	1.98	6.31E-05
ENSMUSG00000029138	4930548H24Rik	0.07	-1.6	8.05E-02	0.01	-3.89	8.03E-05	1.1	3.4	5.51E-20	0.02	-3.88	4.17E-13	0.02	-3.19	1.88E-02	0.01	-3.62	3.87E-03	0.05	-2.01	5.79E-02	0.02	-3.25	6.31E-04	0.03	-2.87	4.10E-02	0.01	-3.75	5.37E-03	1.08	2.75	4.67E-09
ENSMUSG00000097651	4930461G14Rik	0.07	-1.64	5.78E-02	0.04	-2.65	2.42E-03	1.1	3.3	6.42E-20	0	-5.64	2.34E-19	0.08	-1.4	3.67E-01	0.01	-4.08	1.24E-03	0.4	0.93	1.66E-01	0.01	-3.88	6.21E-05	0.09	-1.29	5.50E-01	0.11	-0.98	6.97E-01	0.53	1.4	1.18E-02
ENSMUSG00000112578	RP23-395L3.3	0.11	-1.8	2.51E-02	0.15	-1.44	1.17E-01	0.98	1.68	2.32E-05	0.02	-4.69	1.29E-19	0.04	-3.19	8.34E-03	0.06	-2.61	1.48E-02	1.4	2.25	5.13E-06	0.02	-4.06	8.12E-06	0.43	0.18	1.00E+00	0.93	1.45	1.91E-02	0.35	-0.14	1.00E+00
ENSMUSG00000058586	Serhl	0.18	-1.89	1.45E-02	0.94	0.65	4.01E-01	0.86	0.51	3.36E-01	0.02	-5.21	3.98E-24	0.1	-2.71	1.69E-02	0.18	-1.88	8.83E-02	1.62	1.57	2.16E-03	0.04	-4.04	2.75E-06	1.59	1.5	2.08E-02	1.86	1.79	2.18E-03	0.35	-0.9	5.16E-01
ENSMUSG00000005148	Klf5	0.11	-1.91	2.30E-02	0.06	-2.89	6.65E-04	2.15	3.64	3.82E-25	0.01	-5.65	5.21E-22	0.08	-2.24	7.66E-02	0.02	-3.9	1.08E-03	0.42	0.09	1.00E+00	0.02	-3.9	2.76E-05	0.08	-2.28	8.97E-02	0.13	-1.63	2.33E-01	1.19	1.79	5.38E-04
ENSMUSG00000024970	AI846148	0.12	-1.95	1.25E-02	0.55	0.3	8.87E-01	0.6	0.45	4.40E-01	0.02	-4.47	1.50E-19	0.06	-2.78	1.60E-02	0.07	-2.66	1.08E-02	0.92	1.16	3.99E-02	0.27	-0.79	4.28E-01	1.14	1.5	2.19E-02	1.45	1.94	8.32E-04	0.42	-0.11	1.00E+00
ENSMUSG00000090118	Gm16163	0.06	-2.04	2.03E-02	0.02	-3.29	3.34E-04	1.18	3.43	4.35E-21	0.01	-4.87	5.29E-17	0.04	-2.27	8.22E-02	0.03	-2.9	1.47E-02	0.68	1.83	7.52E-04	0	-4.93	6.37E-06	0.11	-1.08	7.59E-01	0.15	-0.6	1.00E+00	0.12	-0.91	5.79E-01
ENSMUSG00000021098	4930447C04Rik	0.04	-2.04	6.29E-02	0.07	-1.27	4.55E-01	0.03	-2.64	2.66E-03	0.09	-0.94	9.55E-02	0.03	-2.11	2.56E-01	0.06	-1.29	6.10E-01	0.03	-2.26	8.46E-02	1.37	4.65	4.81E-26	0.05	-1.7	5.23E-01	0.07	-1.21	7.29E-01	0.02	-2.75	4.39E-02
ERVB4_1B-I_MM_ERV2_Mus	ERVB4_1B-I_MM_ERV2_Mus	0.11	-2.14	1.00E+00	0.14	-1.83	1.00E+00	0.21	-1.29	1.00E+00	0.16	-1.86	5.77E-01	0.11	-2.08	1.00E+00	0.11	-2.17	1.00E+00	0.23	-1.1	1.00E+00	4.18	4.57	8.60E-05	0.2	-1.3	1.00E+00	0.23	-1.12	1.00E+00	0.24	-1.03	9.63E-01
ENSMUSG00000067101	1700010H22Rik	0.22	-2.16	4.82E-03	0.39	-1.32	1.53E-01	2.66	1.91	6.90E-07	0.02	-5.68	3.09E-27	0.09	-3.37	3.95E-03	0.03	-4.9	6.63E-05	3.2	2.13	1.29E-05	0.04	-4.47	3.60E-07	1.41	0.65	6.19E-01	2.16	1.37	2.47E-02	0.52	-0.88	5.36E-01
ENSMUSG00000069441	Dsg1a	0.09	-2.17	6.92E-03	0.07	-2.56	1.56E-03	1.36	2.37	2.03E-10	0	-6.43	5.10E-25	0.04	-3.22	8.13E-03	0.02	-3.87	8.23E-04	1.45	2.29	4.20E-06	0.02	-3.87	1.62E-05	0.28	-0.49	1.00E+00	0.69	0.91	2.56E-01	0.37	-0.07	1.00E+00
ENSMUSG00000010067	Rassf1	0.38	-2.19	4.56E-03	0.35	-2.33	2.49E-03	0.11	-3.97	4.61E-09	5.02	2.93	4.11E-15	0.1	-4.08	1.04E-03	1.22	-0.44	1.00E+00	0.19	-3.17	5.62E-04	3.3	1.15	1.49E-02	0.24	-2.8	1.75E-02	0.13	-3.73	1.08E-03	0.8	-1.07	3.85E-01
ENSMUSG00000011751	Sptbn4	0.13	-2.2	6.40E-03	0.36	-0.69	7.51E-01	0.04	-3.73	1.56E-07	1.72	2.92	6.90E-23	0.08	-2.83	1.85E-02	1.26	1.31	3.24E-02	0.06	-3.19	1.05E-03	0.15	-1.99	1.41E-02	0.13	-2.1	1.13E-01	0.08	-2.76	1.40E-02	0.08	-2.74	5.71E-03
ENSMUSG00000043419	Chd3os	0.04	-2.23	4.11E-02	0.02	-3.31	3.37E-03	0.02	-3.35	2.41E-04	0.05	-2.17	5.89E-05	0.03	-2.29	1.99E-01	0.07	-1.38	5.64E-01	0	-4.64	1.33E-03	1.94	6.03	9.10E-45	0.01	-3.37	5.59E-02	0.01	-3.86	1.64E-02	0.03	-2.25	1.05E-01
ENSMUSG00000068349	Gml	0.17	-2.27	3.78E-02	0.31	-1.44	3.41E-01	0.23	-1.88	3.00E-02	0.37	-1.28	1.61E-02	0.23	-1.77	4.27E-01	0.22	-1.85	3.41E-01	0.45	-0.85	8.70E-01	6.49	4.4	2.60E-23	0.42	-0.93	1.00E+00	0.32	-1.34	6.61E-01	0.19	-2.07	1.52E-01
ENSMUSG00000020649	Rrm2	0.08	-2.27	4.07E-03	0.69	0.99	9.04E-02	0.2	-1.01	1.56E-01	0.39	0.04	1.00E+00	0.04	-3	1.10E-02	1.08	1.69	1.98E-03	0.4	0.06	1.00E+00	0.04	-3.18	1.40E-04	0.52	0.48	8.60E-01	0.55	0.58	6.21E-01	0.19	-1.05	3.96E-01
ENSMUSG00000028945	Rheb	0.1	-2.31	4.35E-03	0.33	-0.61	8.29E-01	0.08	-2.8	2.69E-05	0.76	0.88	9.75E-03	0.07	-2.76	2.18E-02	1.6	1.97	3.85E-04	0.12	-2.07	2.82E-02	1.38	1.74	1.01E-04	0.3	-0.73	1.00E+00	0.23	-1.1	5.66E-01	0.08	-2.62	7.87E-03
ENSMUSG00000041231	Ublcp1	0.02	-2.32	3.11E-02	0.06	-1.24	4.55E-01	0.02	-2.72	1.97E-03	0.12	-0.12	1.00E+00	0.01	-2.87	8.46E-02	0.08	-0.74	9.74E-01	0.03	-2.18	9.43E-02	1.08	4.38	1.90E-23	0.02	-2.61	1.54E-01	0.02	-2.68	8.79E-02	0	-3.83	6.56E-03
ENSMUSG00000029437	Il31	0.45	-2.35	1.64E-03	1.91	-0.18	1.00E+00	5.14	1.56	8.65E-05	0.22	-3.61	1.95E-17	0.48	-2.22	4.56E-02	0.44	-2.37	1.70E-02	6.83	1.99	7.63E-05	0.2	-3.54	7.75E-06	3.43	0.74	4.91E-01	5.12	1.42	2.20E-02	0.42	-2.41	7.62E-03
ENSMUSG00000030148	Clec4a2	0.14	-2.39	2.53E-03	0.1	-2.95	2.09E-04	2.91	2.84	7.18E-14	0.02	-5.55	4.51E-25	0.09	-2.94	1.23E-02	0.04	-4.11	3.82E-04	2.14	1.92	1.82E-04	0.03	-4.33	1.41E-06	0.48	-0.54	1.00E+00	0.98	0.54	6.72E-01	0.64	-0.11	1.00E+00
ENSMUSG00000023913	Pla2g7	0.07	-2.46	2.21E-03	0.46	0.26	9.48E-01	0.77	1.15	7.60E-03	0	-6.44	2.94E-25	0.06	-2.57	2.95E-02	0.07	-2.55	1.72E-02	1.29	2.04	5.62E-05	0.07	-2.58	1.48E-03	0.76	1.06	1.98E-01	0.94	1.42	2.15E-02	0.19	-1.05	4.02E-01
ENSMUSG00000062329	Cytl1	0.06	-2.46	2.66E-03	0.33	-0.01	1.00E+00	0.4	0.33	6.34E-01	0.03	-3.65	1.76E-14	0.03	-3.2	8.94E-03	0.03	-3.41	2.22E-03	0.92	1.71	1.04E-03	0.03	-3.43	8.92E-05	0.92	1.68	1.14E-02	1.25	2.27	4.53E-05	0.06	-2.35	1.73E-02
ENSMUSG00000041068	4930596D02Rik	0.29	-2.48	1.40E-03	0.23	-2.81	2.61E-04	7.16	3.2	7.18E-14	0.03	-5.8	1.24E-28	0.31	-2.33	4.34E-02	0.05	-4.95	4.95E-05	4.19	1.71	1.17E-03	0.1	-3.93	3.02E-06	0.7	-1.17	5.99E-01	1.6	0.08	1.00E+00	1.5	-0.02	1.00E+00
ENSMUSG00000032065	Tex12	0.07	-2.49	1.00E-02	0.12	-1.76	1.07E-01	0.1	-2.06	7.37E-03	0.21	-1.13	2.13E-02	0.08	-2.36	1.20E-01	0.1	-2.02	1.77E-01	0.09	-2.27	4.65E-02	3.72	4.91	9.88E-35	0.08	-2.37	1.46E-01	0.13	-1.63	3.57E-01	0.07	-2.43	4.34E-02
ENSMUSG00000020733	Slc9a3r1	0.09	-2.5	1.43E-03	0.71	0.52	5.69E-01	0.72	0.55	2.97E-01	0.02	-4.91	5.91E-22	0.03	-3.84	2.05E-03	0.13	-2.02	6.34E-02	1.39	1.67	1.09E-03	0.05	-3.36	4.93E-05	1.44	1.7	9.31E-03	1.52	1.8	2.18E-03	0.15	-1.77	6.88E-02
ENSMUSG00000048174	Tmem81	0.06	-2.53	2.24E-03	0.05	-2.86	6.42E-04	1.51	3.12	1.89E-18	0.03	-3.5	1.77E-13	0.06	-2.44	4.61E-02	0	-5.2	1.85E-04	0.82	1.53	4.16E-03	0.01	-4.42	5.02E-06	0.13	-1.31	5.11E-01	0.3	-0.12	1.00E+00	0.51	0.7	2.89E-01
ENSMUSG00000034509	Mad2l1bp	0.06	-2.58	1.28E-03	1.12	1.89	2.19E-05	0.12	-1.77	5.26E-03	0.35	-0.14	9.60E-01	0.04	-2.99	1.13E-02	0.84	1.27	3.63E-02	0.15	-1.41	1.62E-01	0.2	-0.97	2.80E-01	0.71	1	2.43E-01	0.33	-0.23	1.00E+00	0.23	-0.73	6.82E-01
ENSMUSG00000068600	Gml2	0.07	-2.58	3.94E-02	0.15	-1.58	3.71E-01	0.12	-1.95	5.08E-02	0.21	-1.21	4.63E-02	0.1	-2.15	3.84E-01	0.08	-2.41	2.39E-01	0.28	-0.62	1.00E+00	3.67	4.54	3.85E-21	0.17	-1.37	8.96E-01	0.16	-1.43	7.28E-01	0.09	-2.28	1.78E-01
ENSMUSG000000296	Ptcd1	0.07	-2.66	1.03E-	0.31	-0.59	8.45E-	0.03	-4.19	1.23E-	1.36	2.8	1.11E-	0.03	-3.65	3.47E-	0.72	0.74	3.72E-	0.04	-3.39	5.23E-	0.55	0.32	5.84E-	0.13	-1.84	1.82E-	0.06	-2.87	1.00E-	0.03	-3.61	3.98E-

24				03			01			08			21			03			01			04			01			01			02			04
ENSMUSG00000038503	Mesdc2	0.35	-2.69	4.51E-04	0.85	-1.42	1.06E-01	0.5	-2.23	2.62E-04	6.53	2.86	4.11E-15	0.44	-2.34	3.77E-02	2.06	-0.07	1.00E+00	0.52	-2.14	1.44E-02	2.84	0.44	4.02E-01	0.6	-1.91	1.32E-01	0.57	-1.99	6.43E-02	0.36	-2.64	4.23E-03
ENSMUSG00000062773	Tex101	0.43	-2.71	1.76E-02	0.84	-1.74	2.19E-01	0.4	-2.84	1.40E-03	1.44	-1.03	5.68E-02	0.55	-2.29	2.67E-01	0.78	-1.82	4.01E-01	0.58	-2.26	1.05E-01	24.26	4.94	2.44E-14	0.59	-2.23	3.37E-01	0.59	-2.23	2.33E-01	0.5	-2.47	9.60E-02
ENSMUSG00000058569	Tmed9	0.03	-2.71	2.42E-02	0.08	-1.59	3.20E-01	0.03	-2.89	2.41E-03	0.15	-0.63	3.72E-01	0.06	-1.73	5.06E-01	0.07	-1.7	4.72E-01	0.05	-2.23	1.28E-01	2	4.78	7.61E-25	0.04	-2.51	2.62E-01	0.04	-2.29	2.44E-01	0.02	-2.99	4.73E-02
ENSMUSG00000029682	Spam1	0.26	-2.76	3.21E-04	1.05	-0.69	6.94E-01	5.1	2.12	1.40E-08	0.03	-5.9	4.94E-30	0.23	-2.87	1.03E-02	0.11	-3.87	3.35E-04	5	1.91	1.93E-04	0.05	-5.15	7.80E-09	2.63	0.76	4.83E-01	3.67	1.32	3.01E-02	0.33	-2.36	1.01E-02
MMVL30_Internal	MMVL30_Internal	0.23	-2.77	4.26E-04	4.52	1.95	1.68E-05	0.58	-1.48	2.05E-02	1.04	-0.64	1.51E-01	0.18	-3.05	8.50E-03	1.68	0.18	1.00E+00	1.32	-0.19	1.00E+00	1.92	0.39	4.71E-01	2.63	0.9	3.32E-01	2.07	0.51	6.99E-01	0.32	-2.3	1.53E-02
ENSMUSG00000110576	Gm45787	0.06	-2.78	6.67E-04	0.16	-1.46	1.13E-01	1.36	2.22	3.43E-09	0.01	-6.19	7.36E-25	0.04	-3.11	9.71E-03	0.01	-4.96	1.81E-04	1.39	2.08	3.81E-05	0.03	-3.76	2.06E-05	0.64	0.68	6.05E-01	0.87	1.2	7.05E-02	0.13	-1.71	9.43E-02
ENSMUSG00000060794	Tssk5	0.07	-2.83	6.61E-04	0.06	-3.08	1.99E-04	1.4	1.95	5.50E-07	0.02	-5.14	1.19E-21	0.03	-4	2.20E-03	0.02	-4.17	4.74E-04	1.98	2.51	4.09E-07	0.01	-4.97	4.79E-07	0.59	0.31	1.00E+00	1.28	1.62	7.75E-03	0.04	-3.53	6.27E-04
ENSMUSG00000079389	Gm3149	0.06	-2.86	4.62E-04	1.69	2.35	2.02E-08	0.21	-1.2	7.80E-02	0.01	-6.09	2.81E-25	0.01	-4.69	6.80E-04	0.33	-0.48	1.00E+00	0.69	0.65	3.92E-01	0.01	-4.92	3.84E-07	1.47	1.92	1.71E-03	1.08	1.38	2.58E-02	0.06	-2.83	3.68E-03
ENSMUSG00000079409	Gm5795	0.14	-2.87	2.17E-04	2.77	1.86	2.29E-05	0.71	-0.48	6.85E-01	0.17	-2.83	6.39E-12	0.1	-3.32	3.98E-03	0.54	-0.88	6.72E-01	1.57	0.79	2.19E-01	0.13	-2.92	1.78E-04	2.65	1.67	9.31E-03	2.37	1.48	1.25E-02	0.31	-1.69	7.60E-02
ENSMUSG00000074358	Ccdc61	0.02	-2.92	3.14E-03	0.12	-0.54	9.76E-01	0.03	-2.63	7.82E-04	0.08	-1.3	6.81E-03	0.02	-2.82	4.85E-02	0.05	-1.77	2.48E-01	0.07	-1.36	3.21E-01	1.4	4.4	1.28E-27	0.12	-0.43	1.00E+00	0.1	-0.8	9.66E-01	0.03	-2.38	4.42E-02
ENSMUSG00000078681	Tm2d3	0.02	-2.93	3.78E-03	0.05	-2.04	5.25E-02	0.03	-2.86	4.23E-04	0.19	-0.08	1.00E+00	0.05	-1.87	2.57E-01	0.05	-1.88	2.36E-01	0.04	-2.32	4.52E-02	1.68	4.47	3.11E-27	0.04	-2.36	1.66E-01	0.04	-2.43	8.96E-02	0.04	-2.09	9.29E-02
ENSMUSG00000070520	Nsmce3	0.09	-2.93	3.15E-04	0.12	-2.5	1.54E-03	0.04	-3.9	2.92E-08	1.95	2.94	5.89E-24	0.08	-3.01	1.09E-02	0.89	0.54	5.89E-01	0.06	-3.36	4.75E-04	1.16	0.99	4.44E-02	0.06	-3.29	9.31E-03	0.1	-2.73	1.27E-02	0.05	-3.5	4.39E-04
ENSMUSG00000106086	Gm43352	0.16	-2.98	1.50E-04	0.57	-1.18	2.41E-01	0.13	-3.35	2.36E-07	3.76	2.92	4.11E-15	0.16	-2.91	1.06E-02	1.89	0.69	4.10E-01	0.17	-2.94	1.11E-03	1.33	0.13	8.50E-01	0.31	-2.04	9.86E-02	0.3	-2.11	4.78E-02	0.12	-3.34	4.45E-04
ENSMUSG00000027350	Chgb	0.07	-2.99	2.02E-04	1.62	1.84	3.70E-05	0.06	-3.31	6.86E-07	0.61	0.13	8.31E-01	0.04	-3.75	2.29E-03	0.97	0.85	2.65E-01	0.16	-1.92	3.47E-02	0.91	0.75	1.42E-01	1.01	0.92	3.14E-01	0.53	-0.11	1.00E+00	0.03	-4.13	5.61E-05
ENSMUSG00000022946	Dopey2	0.05	-3	2.86E-04	0.18	-1.28	1.92E-01	1.07	1.67	2.41E-05	0.03	-3.99	8.06E-17	0.03	-3.54	4.05E-03	0.07	-2.55	1.67E-02	1.48	2.19	9.24E-06	0.12	-1.89	1.75E-02	0.66	0.72	5.47E-01	1.09	1.58	8.76E-03	0.06	-2.8	4.21E-03
ENSMUSG00000070369	Itgad	0.1	-3	1.57E-04	0.61	-0.39	1.00E+00	1.52	1.15	6.34E-03	0.06	-3.98	2.33E-18	0.07	-3.44	3.57E-03	0.11	-2.8	5.84E-03	2.68	2.13	1.38E-05	0.07	-3.58	1.45E-05	1.42	0.95	2.68E-01	2.45	1.91	9.84E-04	0.14	-2.53	6.70E-03
ENSMUSG00000068882	Ssb	0.06	-3.02	1.64E-03	0.24	-0.98	5.66E-01	0.05	-3.19	3.86E-05	0.31	-0.66	2.24E-01	0.09	-2.33	1.14E-01	0.22	-1.11	6.51E-01	0.11	-2.11	5.81E-02	3.77	4.4	6.07E-28	0.23	-1.01	9.04E-01	0.15	-1.6	3.55E-01	0.07	-2.71	1.95E-02
ENSMUSG00000021709	Erbin	0.02	-3.08	7.24E-04	0.06	-1.87	4.12E-02	1.17	3.58	2.60E-23	0.04	-2.87	1.55E-09	0.02	-3.21	1.48E-02	0.04	-2.46	3.98E-02	0.23	0.1	1.00E+00	0.11	-1	3.12E-01	0.13	-0.79	9.92E-01	0.08	-1.48	3.36E-01	0.37	0.82	2.11E-01
ENSMUSG00000109587	Gm31105	0.06	-3.1	1.54E-04	0.33	-0.79	6.14E-01	0.08	-2.92	8.51E-06	1.75	3.09	3.20E-26	0.05	-3.34	5.51E-03	0.48	-0.2	1.00E+00	0.11	-2.33	1.00E-02	0.51	-0.1	1.00E+00	0.21	-1.41	4.24E-01	0.18	-1.68	1.68E-01	0.03	-3.89	1.45E-04
ENSMUSG00000045466	Zfp956	0.04	-3.12	2.16E-04	0.19	-1.1	3.33E-01	0.04	-3.49	6.88E-07	1.16	2.76	6.65E-21	0.04	-3.22	8.08E-03	0.71	0.96	1.87E-01	0.05	-3.06	1.44E-03	0.52	0.47	4.08E-01	0.06	-2.7	2.88E-02	0.08	-2.24	4.56E-02	0.03	-3.4	8.42E-04
ENSMUSG00000026622	Nek2	0.38	-3.12	5.22E-05	4.86	0.75	3.16E-01	0.26	-3.7	7.86E-09	6.51	1.64	4.18E-08	0.19	-4.05	7.37E-04	8.69	1.7	2.01E-03	0.36	-3.18	3.90E-04	3.74	0.31	5.78E-01	1.64	-0.96	7.72E-01	0.64	-2.34	2.29E-02	0.27	-3.56	1.23E-04
ENSMUSG00000005233	Spc25	0.04	-3.13	1.78E-04	0.51	0.55	5.36E-01	0.1	-1.91	2.82E-03	0.68	1.32	2.56E-05	0.03	-3.33	6.07E-03	1.01	1.68	2.23E-03	0.2	-0.87	5.85E-01	0.27	-0.46	8.17E-01	0.29	-0.32	1.00E+00	0.25	-0.57	1.00E+00	0.02	-3.7	3.42E-04
ENSMUSG00000086277	4930558K02Rik	0.04	-3.15	2.40E-04	0.02	-4.36	2.41E-06	1.57	2.9	7.38E-16	0.01	-6.02	2.26E-23	0.04	-3.12	1.09E-02	0.02	-3.78	1.10E-03	1.16	1.98	1.37E-04	0.17	-1.18	1.77E-01	0.22	-0.78	9.77E-01	0.42	0.23	1.00E+00	0.32	-0.18	1.00E+00
ENSMUSG00000031270	4930513O06Rik	0.04	-3.17	3.46E-04	0.03	-3.5	8.40E-05	1.7	3.42	8.70E-22	0.01	-5.41	1.69E-20	0.01	-4.23	2.37E-03	0.01	-4.24	7.23E-04	0.41	0.33	8.69E-01	0.02	-4.14	1.65E-05	0.05	-2.83	3.07E-02	0.11	-1.58	2.59E-01	1.29	2.25	3.58E-06
ENSMUSG00000026734	4921504E06Rik	0.24	-3.25	2.94E-05	4.68	1.36	5.90E-03	2.26	0.12	9.66E-01	0.04	-5.9	5.35E-31	0.24	-3.16	4.68E-03	0.73	-1.59	1.67E-01	4.85	1.38	1.00E-02	0.07	-5.01	7.32E-09	5.72	1.64	1.26E-02	5.95	1.72	4.03E-03	0.82	-1.41	1.58E-01
ENSMUSG00000024696	Lpxn	0.06	-3.25	8.93E-05	0.24	-1.23	2.22E-01	0.06	-3.17	2.17E-06	1.66	2.93	7.01E-24	0.06	-3.02	1.06E-02	0.82	0.67	4.42E-01	0.08	-2.82	2.27E-03	0.62	0.23	7.16E-01	0.15	-1.86	1.72E-01	0.08	-2.7	1.35E-02	0.06	-3.15	1.31E-03
ENSMUSG00000034706	Dnaic2	0.12	-3.26	4.25E-05	2.33	1.24	1.30E-02	0.13	-3.2	5.95E-07	2.25	1.5	9.00E-07	0.1	-3.54	2.62E-03	3.23	1.74	1.18E-03	0.26	-2.2	1.18E-02	0.14	-3.09	8.71E-05	0.99	-0.19	1.00E+00	0.48	-1.27	3.86E-01	0.11	-3.39	3.46E-04
ENSMUSG00000037638	Zbtb42	0.05	-3.29	1.29E-04	0.26	-1.02	4.21E-01	0.04	-3.89	8.60E-08	1.65	3.13	1.02E-25	0.06	-2.95	1.54E-02	1.11	1.24	5.06E-02	0.04	-3.84	2.34E-04	0.27	-0.99	2.96E-01	0.08	-2.64	3.49E-02	0.06	-3.16	6.40E-03	0.01	-4.79	2.20E-05
ENSMUSG00000095024	Gm5458	0.14	-3.3	3.01E-05	3.45	1.7	2.04E-04	1.1	-0.26	1.00E+00	0.15	-3.44	6.36E-16	0.1	-3.75	1.56E-03	0.52	-1.38	2.88E-01	2.36	0.98	9.23E-02	0.14	-3.26	3.46E-05	4.1	1.92	1.16E-03	3.19	1.48	1.26E-02	0.31	-2.11	2.18E-02
ENSMUSG00000049719	Prss46	0.42	-3.31	1.80E-05	7.01	0.99	9.12E-02	3.68	-0.08	1.00E+00	0.8	-2.55	8.57E-11	0.4	-3.33	2.90E-03	2.68	-0.56	9.85E-01	9.85	1.56	2.49E-03	0.24	-4.09	3.61E-07	9.49	1.46	2.83E-02	10.93	1.72	3.95E-03	0.7	-2.54	4.32E-03
ENSMUSG00000091255	Speer4e	0.11	-3.31	4.16E-05	3.93	2.35	2.02E-08	0.61	-0.91	2.14E-01	0.06	-4.49	1.18E-21	0.08	-3.79	1.78E-03	3.12	1.74	1.28E-03	1.23	0.21	1.00E+00	0.07	-3.97	2.59E-06	2.2	1.14	1.29E-01	1.58	0.6	5.76E-01	0.21	-2.4	1.04E-02
ENSMUSG00000022394	L3mbtl2	0.05	-3.33	9.28E-05	0.03	-4.04	4.07E-06	0.03	-4.17	1.53E-08	1.57	3.12	5.66E-26	0.04	-3.35	6.32E-03	0.4	-0.31	1.00E+00	0.03	-3.92	1.61E-04	1.15	1.4	2.57E-03	0.05	-3.37	9.31E-03	0.06	-3.09	6.54E-03	0.04	-3.55	5.27E-04
ENSMUSG00000043542	Zc2hc1a	0.05	-3.34	5.14E-05	1.66	2.01	3.78E-06	0.4	-0.45	7.40E-01	0.04	-4.1	2.94E-18	0.08	-2.77	1.60E-02	0.4	-0.44	1.00E+00	0.75	0.54	5.44E-01	0.25	-1.12	1.88E-01	1.53	1.74	6.98E-03	1.17	1.28	4.20E-02	0.12	-2.19	2.13E-02
IAPLTR2a2_Mm_ERV2_Mus	IAPLTR2a2_Mm_ERV2_Mus	0.04	-3.36	9.20E-05	0.74	1.1	4.75E-02	0.29	-0.47	7.34E-01	0.09	-2.34	3.68E-08	0.03	-3.42	5.64E-03	0.2	-1	6.03E-01	0.85	1.31	1.82E-02	0.05	-2.95	4.13E-04	1.11	1.74	8.70E-03	1.07	1.69	4.69E-03	0.19	-1.06	4.01E-01

ENSMUSG00000054717	Hmgb2	0.19	-3.41	2.19E-04	0.35	-2.52	4.73E-03	0.17	-3.58	1.51E-06	2.39	0.47	2.90E-01	0.3	-2.69	4.79E-02	0.56	-1.79	2.15E-01	0.23	-3.11	2.77E-03	14.81	4.24	2.44E-14	0.27	-2.88	3.63E-02	0.27	-2.85	2.14E-02	0.2	-3.3	2.87E-03
ENSMUSG00000046958	4930432E11Rik	0.05	-3.41	7.77E-05	0.04	-3.86	9.63E-06	1.96	2.57	3.01E-12	0.03	-4.18	1.41E-17	0.01	-5.44	2.63E-04	0.12	-2.09	6.68E-02	1.94	2.31	4.20E-06	0.02	-4.5	1.91E-06	0.26	-1.02	7.69E-01	0.92	0.91	2.54E-01	0.33	-0.69	7.73E-01
ENSMUSG00000047115	Fam221a	0.04	-3.43	7.26E-05	0.1	-2.16	8.31E-03	0.04	-3.54	5.30E-07	1.23	2.86	3.56E-22	0.06	-2.75	2.19E-02	0.56	0.51	6.37E-01	0.02	-4.24	8.62E-05	0.84	1.19	1.32E-02	0.04	-3.23	1.19E-02	0.03	-3.63	2.77E-03	0.04	-3.12	1.86E-03
ENSMUSG00000072726	Gm5797	0.08	-3.44	2.61E-05	2.61	2.07	1.39E-06	0.5	-0.78	3.17E-01	0.05	-4.43	5.52E-21	0.06	-3.76	1.91E-03	0.41	-1.04	5.32E-01	1.34	0.8	2.19E-01	0.05	-4.03	2.30E-06	2.62	1.94	1.13E-03	2.07	1.53	1.00E-02	0.11	-2.87	2.28E-03
ENSMUSG00000090643	Gm3453	0.07	-3.46	2.78E-05	3.02	2.48	1.08E-09	0.4	-1.04	1.35E-01	0.02	-5.34	3.83E-25	0.06	-3.57	2.93E-03	0.65	-0.29	1.00E+00	0.97	0.34	8.24E-01	0.02	-4.87	1.13E-07	2.47	1.91	1.59E-03	1.64	1.2	6.28E-02	0.12	-2.69	4.19E-03
ENSMUSG00000029685	Asb15	0.05	-3.53	5.09E-05	0.05	-3.47	4.47E-05	3.12	3.78	3.16E-28	0.02	-5.14	5.51E-22	0.04	-3.55	4.86E-03	0.03	-3.98	6.58E-04	0.96	0.88	1.83E-01	0.02	-4.61	1.26E-06	0.08	-2.86	2.19E-02	0.12	-2.28	4.45E-02	1.35	1.41	8.36E-03
ENSMUSG00000093568	Gm20611	0.03	-3.53	6.04E-05	0.18	-0.99	4.40E-01	0.88	1.68	2.77E-05	0.01	-5.93	5.34E-23	0.03	-3.26	8.25E-03	0.02	-3.96	8.01E-04	1.14	2.06	5.51E-05	0.02	-4.17	7.91E-06	0.65	1.02	2.34E-01	0.98	1.74	4.11E-03	0.1	-1.81	7.44E-02
ENSMUSG00000047044	D030056L22Rik	0.19	-3.53	1.33E-05	0.49	-2.21	3.91E-03	0.21	-3.42	1.18E-07	6.38	2.89	4.11E-15	0.2	-3.44	3.24E-03	3.03	0.59	5.16E-01	0.27	-3.04	7.52E-04	3.77	0.96	6.84E-02	0.31	-2.81	1.65E-02	0.28	-2.96	5.37E-03	0.14	-3.9	5.49E-05
ENSMUSG00000007411	Mark3	0.03	-3.54	4.30E-05	0.59	0.69	3.61E-01	0.42	0.12	9.73E-01	0.04	-3.44	5.48E-14	0.04	-3.02	1.13E-02	0.09	-2.11	5.45E-02	1.01	1.59	2.43E-03	0.17	-1.24	1.46E-01	1.1	1.71	9.31E-03	1.17	1.83	2.10E-03	0.07	-2.48	1.06E-02
ENSMUSG00000091692	4930433I11Rik	0.07	-3.57	1.50E-05	1.95	1.53	9.88E-04	0.66	-0.31	9.64E-01	0.02	-5.37	1.22E-25	0.05	-3.89	1.49E-03	0.26	-1.68	1.46E-01	1.72	1.26	1.90E-02	0.05	-4	2.35E-06	2.33	1.76	5.25E-03	2.23	1.7	4.03E-03	0.48	-0.78	6.33E-01
ENSMUSG00000096372	Gm8138	0.02	-3.57	3.10E-04	1.12	3.16	2.82E-14	0.03	-3.16	4.69E-05	0.01	-4.8	6.98E-16	0.01	-3.63	1.09E-02	1.35	3.26	3.08E-11	0.04	-2.48	1.99E-02	0	-4.86	1.43E-05	0.09	-1.27	6.60E-01	0.06	-1.94	1.63E-01	0.01	-4	9.92E-04
ENSMUSG00000025193	Cutc	0.13	-3.58	7.77E-06	2.51	0.87	1.42E-01	0.21	-2.98	1.47E-06	2.66	1.21	1.00E-04	0.11	-3.7	1.56E-03	3.98	1.61	2.57E-03	0.73	-1.08	3.46E-01	0.91	-0.76	4.40E-01	1.68	0.19	1.00E+00	1.19	-0.34	1.00E+00	0.08	-4.15	1.57E-05
ENSMUSG00000064037	Gpn1	0.04	-3.59	2.37E-05	1.15	1.44	2.51E-03	0.24	-1.15	8.83E-02	0.24	-1.26	1.78E-03	0.04	-3.37	4.86E-03	0.28	-0.84	7.20E-01	0.76	0.68	3.62E-01	0.37	-0.44	8.32E-01	1.44	1.76	5.99E-03	1.13	1.35	2.88E-02	0.06	-3.04	1.67E-03
ENSMUSG00000018543	1700001P01Rik	0.39	-3.6	8.62E-06	0.63	-2.94	1.08E-04	19.88	3.03	7.18E-14	0.14	-5.29	5.01E-28	0.55	-3.07	6.91E-03	0.27	-4.1	2.04E-04	9.63	1.27	2.58E-02	0.22	-4.4	1.76E-07	2.38	-0.95	8.01E-01	4.89	0.14	1.00E+00	9.92	1.28	2.24E-02
ENSMUSG00000090404	Gm8362	0.07	-3.6	1.29E-05	2.71	2.03	2.33E-06	0.53	-0.76	3.35E-01	0.02	-5.48	2.82E-26	0.07	-3.58	2.54E-03	0.68	-0.36	1.00E+00	1.4	0.78	2.39E-01	0.04	-4.5	3.23E-07	2.71	1.9	1.55E-03	2.16	1.5	1.15E-02	0.15	-2.53	6.51E-03
ENSMUSG00000020212	Mdm1	0.12	-3.61	7.40E-06	2.6	1.07	4.41E-02	1.46	0.1	9.86E-01	0.09	-4.22	4.26E-21	0.1	-3.84	1.28E-03	0.4	-1.83	8.91E-02	2.86	1.21	2.47E-02	0.49	-1.55	4.29E-02	4.05	1.78	4.03E-03	3.87	1.71	3.29E-03	0.74	-0.94	4.65E-01
ENSMUSG00000024056	Ndc80	0.03	-3.61	3.43E-05	0.35	-0.04	1.00E+00	0.07	-2.43	1.86E-04	0.77	1.62	1.32E-07	0.06	-2.46	3.53E-02	1.06	1.74	1.48E-03	0.12	-1.6	1.01E-01	0.39	0.11	9.13E-01	0.19	-0.94	8.18E-01	0.26	-0.53	1.00E+00	0.04	-2.98	2.55E-03
ENSMUSG00000073821	8030451A03Rik	0.07	-3.63	1.15E-05	1.41	0.77	2.50E-01	0.89	0.01	1.00E+00	0.15	-2.87	6.20E-12	0.07	-3.62	2.43E-03	0.48	-0.91	6.54E-01	2.33	1.62	1.44E-03	0.09	-3.38	2.92E-05	2.41	1.65	1.06E-02	2.6	1.79	2.18E-03	0.17	-2.39	9.96E-03
ENSMUSG00000053604	Rpia	0.01	-3.65	2.15E-03	0.03	-2.22	6.33E-02	0.02	-3.01	1.05E-03	0.08	-0.93	1.08E-01	0.01	-2.96	8.35E-02	0.04	-1.78	3.61E-01	0.02	-2.96	2.52E-02	1.37	5.23	5.77E-33	0.04	-1.84	4.65E-01	0.03	-2.28	1.92E-01	0.01	-2.92	3.50E-02
ENSMUSG00000090102	Gm4985	0.03	-3.65	3.21E-05	0.24	-0.71	7.05E-01	0.91	1.57	9.30E-05	0.01	-6.05	3.98E-24	0.05	-2.79	1.84E-02	0.01	-4.4	3.42E-04	1.2	2	8.71E-05	0.01	-4.62	1.39E-06	0.69	0.97	2.77E-01	1	1.61	7.42E-03	0.3	-0.34	1.00E+00
ENSMUSG00000022422	Dscc1	0.06	-3.66	1.63E-05	0.13	-2.66	6.94E-04	3.31	2.96	7.18E-14	0.04	-4.42	3.58E-20	0.08	-3.28	5.78E-03	0.02	-5.09	7.04E-05	2.12	1.74	7.36E-04	0.08	-3.3	6.46E-05	0.47	-0.74	9.98E-01	1.12	0.61	5.75E-01	0.75	-0.02	1.00E+00
ENSMUSG00000041431	Ccnb1	0.32	-3.67	3.31E-06	7.96	1.24	1.50E-02	2.23	-0.85	2.38E-01	3.87	0.03	1.00E+00	0.25	-3.99	6.83E-04	10.43	1.64	2.69E-03	3.81	0	1.00E+00	0.47	-3.1	4.19E-05	5.83	0.67	6.83E-01	5.23	0.49	8.21E-01	0.59	-2.77	1.86E-03
ENSMUSG00000025785	Exosc7	0.01	-3.67	2.04E-03	0.03	-2.24	6.00E-02	0.01	-3.85	7.92E-05	0.06	-1.53	4.85E-03	0.03	-1.98	2.97E-01	0.01	-3.42	2.98E-02	0.02	-2.99	2.39E-02	1.54	5.94	2.39E-42	0.02	-2.73	1.48E-01	0.01	-3.54	2.81E-02	0	-3.95	7.24E-03
ENSMUSG00000022538	Lsg1	0.05	-3.68	1.24E-05	1.07	0.77	2.52E-01	0.07	-3.44	2.36E-07	1.46	1.71	2.03E-08	0.07	-3.22	5.67E-03	1.82	1.63	2.69E-03	0.07	-3.3	4.48E-04	0.75	0.17	8.05E-01	0.44	-0.65	1.00E+00	0.12	-2.54	1.69E-02	0.03	-4.18	3.81E-05
ENSMUSG00000099354	1700124L16Rik	0.06	-3.68	9.93E-06	0.64	-0.38	1.00E+00	1.87	1.46	2.40E-04	0.03	-5.09	2.06E-24	0.1	-3.08	7.12E-03	0.25	-1.76	1.19E-01	2.75	2.1	1.92E-05	0.04	-4.41	4.71E-07	1.4	0.85	3.78E-01	2.13	1.58	7.42E-03	0.33	-1.36	1.90E-01
ENSMUSG00000000751	Rpa1	0.19	-3.71	3.90E-06	3.79	0.79	2.74E-01	0.3	-3.1	5.78E-07	4.69	1.48	9.43E-07	0.22	-3.48	2.30E-03	6.65	1.7	1.96E-03	0.85	-1.54	9.20E-02	1.29	-0.92	2.97E-01	2.09	-0.19	1.00E+00	1.37	-0.82	7.83E-01	0.22	-3.49	1.57E-04
ENSMUSG00000022369	Mtbp	0.05	-3.73	9.54E-06	0.9	0.54	5.39E-01	0.88	0.52	3.23E-01	0.13	-2.59	3.64E-10	0.04	-3.75	2.03E-03	0.14	-2.26	2.97E-02	1.37	1.24	2.29E-02	0.4	-0.72	4.89E-01	1.71	1.59	1.34E-02	1.91	1.8	2.18E-03	0.14	-2.26	1.59E-02
ENSMUSG00000107042	Gm42740	0.01	-3.77	5.77E-04	0.05	-1.65	1.53E-01	0	-4.96	7.83E-07	0.2	0.54	2.13E-01	0.01	-3.09	4.10E-02	0.03	-2.28	1.17E-01	0	-4.42	1.04E-03	1.21	4.24	3.53E-24	0.05	-1.67	4.64E-01	0.03	-2.21	1.36E-01	0	-5.05	5.61E-04
ENSMUSG00000028145	Them4	0.04	-3.79	9.93E-06	0.45	-0.49	9.48E-01	0.06	-3.57	1.36E-07	1.86	2.79	3.56E-22	0.06	-3.23	6.11E-03	0.93	0.63	4.84E-01	0.13	-2.29	1.06E-02	0.69	0.17	8.10E-01	0.2	-1.66	2.48E-01	0.12	-2.42	2.41E-02	0.02	-4.75	9.30E-06
ENSMUSG00000057173	Rfx8	0.2	-3.81	2.24E-06	5.87	1.37	5.03E-03	0.4	-2.84	2.99E-06	3.32	0.46	2.26E-01	0.25	-3.44	2.34E-03	7.29	1.67	2.25E-03	1.78	-0.6	8.69E-01	0.67	-2.05	5.74E-03	4.21	0.75	5.62E-01	3.29	0.35	9.08E-01	0.12	-4.44	3.57E-06
ENSMUSG00000093668	Pou5f2	0.09	-3.83	1.88E-05	0.13	-3.29	9.65E-05	0.05	-4.63	9.02E-10	4.6	4.12	2.15E-40	0.1	-3.67	4.32E-03	0.55	-1.21	4.92E-01	0.09	-3.83	2.27E-04	1.49	0.3	6.04E-01	0.07	-4.16	1.60E-03	0.08	-3.98	1.25E-03	0.07	-4.14	1.12E-04
ENSMUSG00000110310	4930518J21Rik	0.02	-3.83	2.33E-05	0.49	0.44	7.12E-01	0.32	-0.23	1.00E+00	0.01	-5.55	3.62E-22	0.01	-4.96	6.53E-04	0.03	-3.38	2.33E-03	0.96	1.59	2.75E-03	0.01	-4.61	2.12E-06	1.35	2.17	2.05E-04	1.43	2.3	4.53E-05	0.04	-3.16	1.93E-03
ENSMUSG00000042607	Asb4	0.06	-3.85	7.20E-06	1.44	0.97	9.80E-02	0.26	-1.75	5.47E-03	1.63	1.51	1.41E-06	0.07	-3.49	3.60E-03	2.1	1.55	5.20E-03	0.2	-2.09	2.05E-02	0.13	-2.71	6.58E-04	0.68	-0.27	1.00E+00	0.52	-0.68	9.71E-01	0.22	-1.9	4.94E-02
ENSMUSG00000028701	Lurap1	0.09	-3.85	3.39E-06	1.01	-0.4	1.00E+00	2.5	1.13	6.57E-03	0.07	-4.62	3.77E-23	0.18	-2.89	9.91E-03	0.1	-3.72	4.47E-04	4.23	2.03	5.51E-05	0.1	-3.82	3.27E-06	2.85	1.27	6.33E-02	3.95	1.85	1.25E-03	0.42	-1.68	8.06E-02
ENSMUSG000000793	Gm3476	0.05	-3.86	6.24E-	2.36	2.16	3.43E-	0.59	-0.28	9.96E-	0.02	-5.28	1.23E-	0.06	-3.42	3.86E-	0.5	-0.51	1.00E+	1.11	0.73	2.95E-	0.02	-4.72	2.20E-	1.94	1.66	1.04E-	1.65	1.38	2.41E-	0.18	-1.99	3.59E-

71				06			07			01			24			03			00			01			07			02			02			02
ENSMUSG00000086796	4932702P03Rik	0.07	-3.88	8.03E-06	0.47	-1.23	2.38E-01	0.11	-3.4	4.78E-07	3.5	3.32	4.07E-29	0.06	-4.13	1.38E-03	0.87	-0.29	1.00E+00	0.18	-2.66	3.85E-03	1.26	0.28	6.24E-01	0.26	-2.08	1.10E-01	0.16	-2.74	1.27E-02	0.08	-3.65	2.80E-04
ENSMUSG00000100557	1700029E06Rik	0.06	-3.89	9.45E-06	0.04	-4.72	9.85E-08	5.29	3.83	7.18E-14	0.02	-5.61	1.45E-25	0.08	-3.59	3.77E-03	0.02	-5.4	4.84E-05	2.02	1.26	2.51E-02	0.03	-4.77	3.10E-07	0.12	-3.07	1.35E-02	0.47	-1.06	6.12E-01	1.52	0.75	2.24E-01
ENSMUSG00000029504	Ddx51	0.03	-3.93	1.87E-05	0.11	-2.14	1.07E-02	0.05	-3.27	3.02E-06	1.63	3.76	2.84E-34	0.06	-2.92	1.78E-02	0.63	0.52	6.27E-01	0.06	-2.89	2.81E-03	0.22	-1.11	2.24E-01	0.06	-2.91	2.19E-02	0.03	-3.99	1.58E-03	0.02	-4.3	9.97E-05
ENSMUSG00000095384	1700001F09Rik	0.16	-3.95	1.39E-06	7	1.95	9.10E-06	1.91	-0.31	9.52E-01	0.06	-5.71	3.53E-30	0.18	-3.73	1.40E-03	1.29	-0.89	6.58E-01	4.11	0.93	1.55E-01	0.1	-4.6	5.01E-08	6.81	1.78	5.80E-03	5.92	1.54	1.14E-02	0.56	-2.12	2.01E-02
ENSMUSG00000022018	Rgcc	0.06	-3.96	2.70E-06	1.83	1.13	2.89E-02	0.12	-3.08	1.20E-06	1.49	0.95	3.72E-03	0.08	-3.47	2.91E-03	2.46	1.59	3.32E-03	0.53	-0.87	5.55E-01	0.17	-2.48	1.17E-03	1.42	0.67	5.79E-01	1.02	0.15	1.00E+00	0.01	-5.65	2.21E-07
ENSMUSG00000100282	1700041C23Rik	0.32	-4.02	9.21E-07	3.31	-0.6	6.98E-01	12.15	1.64	2.77E-05	0.14	-5.45	1.06E-29	0.41	-3.61	1.77E-03	0.33	-3.93	2.30E-04	16.8	2.15	1.12E-05	0.25	-4.35	1.34E-07	8.77	0.94	3.35E-01	13.14	1.65	5.83E-03	0.51	-3.32	2.93E-04
ENSMUSG00000078639	Gm12695	0.02	-4.02	1.28E-05	1.3	2.31	4.84E-08	0.1	-1.91	3.23E-03	0.01	-5.32	1.45E-21	0.01	-4.34	1.45E-03	0.18	-1.06	5.45E-01	0.41	0.21	1.00E+00	0.02	-4.25	5.58E-06	1.44	2.35	2.09E-05	0.94	1.57	9.85E-03	0.04	-3.12	1.94E-03
ENSMUSG00000015176	Nolc1	0.03	-4.03	4.55E-06	1.07	1.13	3.39E-02	0.02	-4.99	7.68E-11	1.08	1.44	2.48E-06	0.03	-3.93	1.71E-03	1.5	1.64	2.68E-03	0.05	-3.45	3.70E-04	0.64	0.24	6.97E-01	0.44	-0.34	1.00E+00	0.16	-1.79	1.17E-01	0.02	-4.57	1.63E-05
ENSMUSG00000104900	4930596I21Rik	0.02	-4.03	1.15E-05	0.12	-1.66	5.92E-02	0.92	1.65	3.61E-05	0.01	-5.33	1.07E-21	0.01	-4.35	1.38E-03	0.02	-3.77	1.02E-03	1.37	2.32	3.28E-06	0	-5.58	1.45E-07	0.49	0.47	8.81E-01	0.97	1.61	7.73E-03	0.35	-0.07	1.00E+00
ENSMUSG00000026301	Iqca	0.06	-4.06	1.79E-06	3.02	1.84	4.86E-05	0.79	-0.46	7.18E-01	0.04	-5.11	2.85E-25	0.04	-4.46	4.53E-04	0.89	-0.27	1.00E+00	1.69	0.75	2.60E-01	0.02	-5.31	9.17E-09	3.54	2.02	5.01E-04	2.64	1.5	1.15E-02	0.22	-2.33	1.14E-02
ENSMUSG00000035910	Dcdc2a	0.04	-4.08	3.01E-06	1.55	1.54	8.80E-04	0.15	-2.16	5.04E-04	0.37	-0.91	2.72E-02	0.03	-4.13	1.11E-03	0.55	-0.21	1.00E+00	0.76	0.31	8.84E-01	0.66	0.06	9.70E-01	1.88	1.81	4.03E-03	1.25	1.1	1.01E-01	0.01	-5.1	2.82E-06
ENSMUSG00000097086	Gm7672	0.02	-4.08	1.08E-05	1.27	2.16	9.13E-07	0.3	-0.38	8.62E-01	0.02	-4.68	3.76E-19	0.01	-4.99	6.36E-04	0.82	1.23	5.17E-02	0.65	0.88	1.81E-01	0.01	-5.05	5.83E-07	0.61	0.76	5.09E-01	0.82	1.24	5.93E-02	0.05	-2.77	5.52E-03
ENSMUSG00000028688	Toe1	0.03	-4.09	4.38E-06	0.87	0.69	3.53E-01	0.06	-3.32	7.55E-07	1.33	1.91	1.44E-10	0.02	-4.58	6.74E-04	1.62	1.7	1.77E-03	0.1	-2.6	4.10E-03	0.34	-0.77	4.58E-01	0.34	-0.79	9.54E-01	0.13	-2.15	4.89E-02	0.05	-3.49	4.45E-04
ENSMUSG00000031609	Sap30	0.03	-4.1	7.74E-06	0.05	-3.59	2.82E-05	0.03	-4.54	2.60E-09	1.71	2.75	2.79E-20	0.05	-3.41	6.13E-03	0.34	-0.79	8.10E-01	0.05	-3.62	3.70E-04	1.89	2.02	4.05E-06	0.05	-3.61	6.53E-03	0.05	-3.43	4.03E-03	0.05	-3.37	1.00E-03
ENSMUSG00000101314	Gm29663	0.01	-4.1	8.36E-05	0.01	-3.69	1.90E-04	1	3.55	1.38E-21	0	-5.4	4.41E-17	0.02	-3	2.96E-02	0.01	-3.43	6.90E-03	0.29	0.69	4.26E-01	0.01	-3.65	2.73E-04	0.02	-3.16	2.74E-02	0.09	-1.05	6.91E-01	0.58	1.82	8.23E-04
ENSMUSG00000031583	Wrn	0.03	-4.12	3.42E-06	0.89	0.89	1.44E-01	0.13	-2.13	6.59E-04	0.92	1.18	1.60E-04	0.04	-3.63	2.82E-03	1.41	1.63	2.75E-03	0.2	-1.44	1.43E-01	0.36	-0.55	6.91E-01	0.6	0.23	1.00E+00	0.35	-0.6	1.00E+00	0.04	-3.48	3.95E-04
ENSMUSG00000072722	Gm6588	0.06	-4.15	1.63E-06	1.89	1.07	4.77E-02	0.39	-1.47	2.04E-02	0.02	-5.61	5.16E-27	0.09	-3.47	3.32E-03	0.09	-3.55	8.23E-04	2.63	1.62	1.47E-03	0.04	-4.57	2.46E-07	3.54	2.13	1.69E-04	3.8	2.28	4.53E-05	0.04	-4.58	7.90E-06
ENSMUSG00000100916	Lhb	0.02	-4.18	3.64E-06	1.41	1.93	1.29E-05	0.19	-1.39	3.26E-02	0.19	-1.49	2.06E-04	0.03	-3.71	2.66E-03	0.56	0.27	9.27E-01	0.66	0.55	5.41E-01	0.04	-3.48	3.76E-05	1.3	1.66	1.10E-02	1.02	1.25	4.92E-02	0.06	-2.86	3.05E-03
ENSMUSG00000079705	Ssxb1	0.03	-4.28	2.47E-06	1.89	2.1	1.28E-06	0.04	-3.87	3.64E-08	0.01	-5.83	1.85E-25	0.02	-4.6	6.67E-04	0.31	-0.95	6.26E-01	0.76	0.43	7.13E-01	0.04	-3.92	7.12E-06	2.29	2.34	2.09E-05	1.87	1.96	8.26E-04	0.03	-4.16	6.27E-05
ENSMUSG00000058173	Smco4	0.09	-4.31	4.17E-07	2.23	0.51	5.69E-01	1.76	0.13	9.45E-01	0.05	-5.31	2.56E-27	0.16	-3.38	3.22E-03	0.19	-3.15	1.66E-03	4.99	1.91	1.98E-04	0.12	-3.82	2.71E-06	4.69	1.74	8.70E-03	5.61	2.09	3.08E-04	0.17	-3.33	3.63E-04
IAPLTR1a_I_MM_ERV2_Mus	IAPLTR1a_I_MM_ERV2_Mus	0.32	-4.35	1.82E-07	11.92	1.15	3.45E-02	2.9	-1.15	5.38E-02	2.15	-1.73	4.91E-06	0.09	-6.06	1.95E-06	1.88	-1.75	1.15E-01	13.71	1.36	1.20E-02	3.05	-1.04	2.20E-01	16.85	1.69	1.01E-02	17.47	1.77	3.13E-03	0.83	-2.94	1.23E-03
ENSMUSG00000020974	Pole2	0.02	-4.37	1.61E-06	0.84	0.72	3.16E-01	0.65	0.32	6.35E-01	0.05	-3.88	4.98E-17	0.05	-3.32	5.17E-03	0.1	-2.52	1.54E-02	1.52	1.75	5.92E-04	0.05	-3.55	2.43E-05	1.41	1.57	1.60E-02	1.81	2.04	3.49E-04	0.03	-3.87	1.31E-04
ENSMUSG00000023165	Ssxb2	0.02	-4.38	1.85E-06	1.61	1.9	2.11E-05	0.04	-3.98	2.27E-08	0.01	-5.59	1.88E-24	0.04	-3.7	2.81E-03	0.24	-1.2	4.34E-01	0.88	0.77	2.63E-01	0.02	-4.57	8.09E-07	2.17	2.36	2.09E-05	1.84	2.05	3.49E-04	0	-5.89	8.32E-07
ENSMUSG00000027469	Tpx2	0.03	-4.46	6.39E-07	0.7	-0.08	1.00E+00	0.05	-4.05	4.58E-09	1.8	2.03	7.26E-12	0.03	-4.62	4.23E-04	1.93	1.56	4.36E-03	0.16	-2.31	8.81E-03	0.95	0.4	4.69E-01	0.4	-0.92	8.24E-01	0.23	-1.74	1.29E-01	0.03	-4.51	1.20E-05
ENSMUSG00000038025	Phf2	0.02	-4.46	2.33E-06	0.07	-3.13	1.52E-04	0.03	-4.41	4.49E-09	1.82	3.05	1.21E-24	0.03	-4.26	1.44E-03	0.91	0.73	3.91E-01	0.05	-3.62	3.53E-04	1.12	1.07	2.89E-02	0.04	-3.75	4.58E-03	0.03	-4.14	9.84E-04	0.02	-4.64	2.66E-05
ENSMUSG00000079093	4930449I24Rik	0.01	-5.02	8.05E-07	1.1	1.97	1.24E-05	0.36	-0.01	1.00E+00	0.01	-5	1.82E-20	0.02	-3.92	2.62E-03	0.38	0.09	1.00E+00	0.75	1.2	3.53E-02	0.02	-4.25	5.32E-06	0.82	1.31	6.42E-02	0.86	1.4	2.48E-02	0	-5.3	9.05E-06
ENSMUSG00000098176	Ccdc166	0.01	-5.03	2.74E-07	1.31	1.72	1.49E-04	0.29	-0.81	3.04E-01	0.08	-2.91	1.44E-11	0.1	-2.33	4.52E-02	0.3	-0.73	8.26E-01	0.9	1	9.32E-02	0.07	-2.73	6.71E-04	1.53	1.91	1.63E-03	1.22	1.51	1.25E-02	0.07	-2.7	4.73E-03
ENSMUSG00000055102	Zfp819	0.01	-5.05	7.42E-07	0.85	1.43	3.57E-03	0.14	-1.44	3.06E-02	0.02	-4.43	2.92E-18	0.03	-3.62	4.23E-03	0.11	-1.71	1.67E-01	0.8	1.26	2.40E-02	0.04	-3.13	2.22E-04	1.26	2.06	5.47E-04	1.32	2.16	1.68E-04	0.03	-3.51	6.70E-04
ENSMUSG00000032254	Kif23	0.02	-5.14	4.38E-08	1.55	1.13	3.02E-02	0.21	-2.05	8.40E-04	1.09	0.63	6.64E-02	0.03	-4.45	5.39E-04	2.43	1.86	5.21E-04	0.53	-0.6	8.90E-01	0.28	-1.54	4.81E-02	1.09	0.51	8.15E-01	0.82	0.06	1.00E+00	0.05	-3.95	6.24E-05
ENSMUSG00000028212	Ccne2	0.01	-5.27	4.26E-06	0.04	-3.59	2.12E-04	0.13	-1.88	1.22E-02	0.37	-0.27	7.84E-01	0.07	-2.57	7.39E-02	0.1	-2.12	1.32E-01	0.04	-3.58	1.60E-03	3.85	4.75	1.89E-33	0.06	-2.83	5.49E-02	0.04	-3.39	1.14E-02	0.16	-1.42	2.95E-01
ENSMUSG00000070315	4930581F22Rik	0.01	-5.91	2.98E-07	0.07	-3.42	2.05E-04	0.02	-4.92	5.73E-09	2.32	3.51	1.65E-27	0.01	-5.22	9.86E-04	0.12	-2.47	5.17E-02	0.03	-4.55	1.33E-04	1.67	1.51	2.50E-03	0.05	-3.7	1.13E-02	0.04	-4.19	2.18E-03	0.01	-5.18	3.56E-05
