# Supplementary material for: Characterization of germ cell differentiation in the male mouse through single-cell RNA sequencing
Source: Sci Rep. 2018 Apr 25;8:6521. doi: 10.1038/s41598-018-24725-0 (PMC5916943; doi:10.1038/s41598-018-24725-0)
Supplement: Supplementary file 3 — Supplementary data table 2 [file 41598_2018_24725_MOESM3_ESM.pdf]

Gene name	Early Sgonia	Late Sgonia	Early Scytes	Late Scytes	Round Stids	Later Stids	Sertoli	Leydig	Method	Source
Zbtb16	1	0	0	0	0	0	0	0	In situ	[1], [2]
Sycp3	1	1	1	1	1	1	NA	NA	qPCR	[3]
Pygo2	1	1	0	0	0	1	1	1	IHC	[4]
Etv5	1	0	0	0	0	0	1	0	IHC	[5]
Bsg	0	0	1	1	1	1	0	0	In situ	[6]
Hormad2	0	0	1	1	0	0	0	0	IHC	[7]
Id4	1	0	0	1	0	0	0	0	IHC	[8]
Id4	1	0	0	0	0	0	0	0	IHC	[9]
Hormad1	0	0	1	1	0	0	0	0	IHC	[7]
Bik	1	1	1	1	0	0	1	0	In situ	[10]
Dazl	1	1	0	0	0	0	0	0	In situ	[11]
Dazl	1	1	1	1	0	0	0	0	IHC	[12]
Tex19.1	0	0	1	1	0	0	0	0	In situ	[13]
Tex19.2	0	0	1	1	0	0	0	0	In situ	[13]
Rad51	1	1	1	0	0	0	0	0	IHC	[14]
Stra8	1	1	0	0	0	0	0	0	In situ	[15]
Stra8	1	1	0	0	0	0	0	0	IHC	[15]

Sox3	1	0	0	0	0	0	0	0	IHC	[16]
H3f3a	1	1	1	1	1	1	0	0	In situ	[17]
Crem	0	0	1	1	1	1	0	0	In situ	[18]
Scml2	1	1	1	1	1	0	0	0	IHC	[19]
Gsg2	0	0	0	0	1	1	0	0	IHC	[20]
Gsg2	0	0	0	1	1	1	0	0	qPCR	[21]
Kdm3a	0	0	0	1	0	0	0	0	IHC	[22]
Kdm3a	0	0	0	1	1	1	1	0	IHC	[23]
Spo11	0	0	1	1	1	0	0	0	Northern	[24]
Hist1h1a	0	0	1	1	0	0	0	0	In situ	[25]
Hist1h1a	0	0	1	1	0	0	0	0	IHC	[25]
Hist1h1a	1	1	0	0	0	0	0	0	In situ	[26]
Boll	0	0	1	1	1	0	0	0	IHC	[27,28]
Pcna	1	1	1	0	0	0	0	0	IHC	[29]
Pcna	1	1	1	1	1	1	0	0	Western	[29]
Clgn	0	0	1	1	1	1	NA	NA	qPCR	[3]
Clgn	0	0	0	1	1	1	NA	NA	Northern	[30]
Stag3	0	0	1	1	0	0	0	0	IHC	[31] [monkey sections, mouse spreads]

Stag3	0	0	1	1	0	0	0	0	Northern	[31]
Tex11	1	1	1	1	1	1	NA	NA	qPCR	[3]
Tex11	0	1	1	0	0	0	0	0	IHC	[32]
Rec8	0	0	1	1	1	1	0	0	IHC	[33] [rat]
Rec8	0	0	1	1	1	1	0	0	In situ	[34] [mouse]
Acrv1	0	0	1	1	1	1	NA	NA	qPCR	[3]
Acrv1	0	0	0	0	1	1	0	0	IHC	[35]
Taf7l	1	1	1	1	1	1	NA	NA	qPCR	[3]
Taf7l	1	1	0	1	1	1	0	0	IHC	[36]
Slxl1	0	0	0	0	1	1	0	0	IHC	[37]
Zfy1	0	0	1	0	1	1	0	0	IHC	[38]
Sohlh2	1	0	0	0	0	0	0	0	IHC	[39]
Epcam	1	NA	NA	NA	NA	NA	NA	NA	Transplantation	[40] [rat]
H3f3b	0	0	1	1	0	0	0	0	In situ	[17,41]
Dmc1	0	0	1	1	0	0	0	0	In situ	[42]
Dmc1	0	0	1	0	0	0	0	0	IHC	[43]
Tnp1	0	0	0	0	1	1	0	0	In situ	[44]
Tnp1	0	0	0	0	0	1	0	0	IHC	[44]

Jag1	0	0	0	1	0	1	0	0	IHC	[45]
Prm1	0	0	0	0	1	1	NA	NA	qPCR	[3]
Prm1	0	0	0	0	0	1	0	0	In situ	[46]
Actb	1	1	1	1	1	1	NA	NA	qPCR	[3]
Tnp2	0	0	0	0	1	1	0	0	In situ	[47]
Sohlh1	1	0	0	0	0	0	0	0	IHC	[48]
Pgk2	0	0	1	1	1	1	NA	NA	qPCR	[3]
Pgk2	0	0	0	1	1	0	0	0	In situ	[49]
Sly	0	0	0	0	1	1	NA	NA	qPCR	[50]
Sly	0	0	0	1	1	1	1	0	In situ	[51]
Sly	0	0	0	0	1	1	0	0	IHC	[51]
Nanos3	1	1	0	0	0	0	0	0	IHC	[52]
Hils1	0	0	0	0	1	0	0	0	In situ	[53]
Hils1	0	0	0	0	0	1	0	0	IHC	[53]
Kit	1	1	1	0	0	0	0	1	In situ	[54]
Zfy2	0	0	1	0	1	1	0	0	In situ	[38]
Taf4b	1	1	0	0	1	1	NA	NA	IHC	[55]
Dyrk4	0	0	0	0	0	1	0	0	In situ	[56]

Pou5f1	1	0	0	0	0	0	0	0	IHC	57
Pou5f1	1	0	0	0	1	1	0	0	Reporter	58
Camk4	1	1	0	0	1	1	0	0	IHC	59
Pgk1	1	1	1	1	0	0	1	1	In situ	49
Uba1	1	1	1	0	1	1	NA	NA	qPCR	3
Rad21	1	1	0	0	1	1	1	1	In situ	34
E2f1	1	1	1	0	0	0	0	0	In situ	60
Gpat2	0	0	1	0	0	0	0	0	In situ	61
Hist1h3a	1	1	0	0	0	0	0	0	In situ	26
Actl11	0	0	0	1	1	1	0	0	In situ	62
Utp3	0	0	0	0	1	1	0	0	In situ	63
Wsb2	1	1	1	0	0	0	0	0	In situ	64
Mitf	1	1	1	1	1	0	0	0	In situ	65
Klf17	0	0	0	0	1	0	0	0	In situ	66
Msh4	0	0	0	1	1	1	0	0	qPCR	67
Pmfbp1	0	0	0	0	1	1	0	0	In situ	68
Pbx4	0	0	1	1	0	0	0	0	In situ	69

Pbx4	0	0	1	1	0	0	0	0	IHC	[69]
Spz1	1	1	1	1	1	1	1	1	In situ	[70]
Wdr33	1	1	1	1	1	0	0	0	In situ	[71] [rat]
Zfp318	0	0	1	1	1	1	0	0	In situ	[72]
Zfp318	0	0	1	1	1	1	0	0	qPCR	[72]
Znrf4	0	0	0	0	1	1	0	0	In situ	[73]
Hspa1l	0	0	0	0	0	1	0	0	In situ	[74]
Hspa1l	0	0	0	0	1	1	0	0	IHC	[74]
Limk2	0	0	0	1	1	0	0	0	In situ	[75] [truncated transcript]
Nr6a1	0	0	1	1	1	1	0	0	In situ	[76]
Nr6a1	0	0	1	1	1	1	0	0	IHC	[76]
Esx1	1	1	0	0	1	0	0	0	In situ	[77]
Stat4	0	0	0	0	1	1	0	0	In situ	[78]
Stat4	0	0	0	0	1	1	0	0	IHC	[78]
Hspa2	0	0	1	1	0	0	0	0	In situ	[79]
Hspa2	0	0	1	1	1	0	0	0	IHC	[79]
Mea1	0	0	1	1	1	1	0	0	In situ	[80]

Mea1	0	0	0	0	1	1	0	0	IHC	[80]
Hsf2	0	0	1	1	1	0	0	0	In situ	[81]
Crisp2	0	0	0	0	1	0	0	0	In situ	[82]
Fer	0	0	1	1	0	0	0	0	In situ	[83]
Zfp35	0	0	1	1	0	0	0	0	Northern	[84]
Usp26	1	1	1	0	0	0	NA	NA	qPCR	[3]
Tktl1	1	1	1	0	1	1	NA	NA	qPCR	[3]
Nxf2	1	1	1	1	1	1	NA	NA	qPCR	[3]
Nxf2	1	1	1	1	0	0	0	0	IHC	[41]
Sall4	1	1	1	0	0	0	NA	NA	qPCR	[3]
Sycp2	0	0	1	1	0	0	NA	NA	qPCR	[3]
Sycp1	1	1	1	1	1	1	NA	NA	qPCR	[3]
Sycp1	0	0	1	1	0	0	0	0	Reporter	[85]
Stk31	1	1	1	1	1	1	NA	NA	qPCR	[3]
Stk31	1	1	1	1	1	1	0	0	IHC	[86]
Tuba3a	0	0	1	1	1	1	NA	NA	qPCR	[3]
Nlrp4c	1	1	1	0	0	0	NA	NA	qPCR	[3]

Tex15	1	1	1	0	1	1	NA	NA	qPCR	[3]
Tex12	1	1	1	1	0	0	NA	NA	qPCR	[3]
Tex12	0	0	0	1	1	0	0	0	IHC	[87]
Tex14	1	1	1	1	1	1	NA	NA	qPCR	[3]
Tex14	0	1	1	1	1	0	0	0	In situ	[88]
Tex14	1	1	1	1	1	1	0	0	IHC	[89]
Ddx4	1	1	1	1	1	1	NA	NA	qPCR	[3]
Ddx4	0	0	1	1	1	0	0	0	In situ	[90]
Ddx4	0	0	1	1	1	0	0	0	IHC	[90]
Rnf17	1	1	1	1	1	1	NA	NA	qPCR	[3]
Rnf17	1	1	1	1	1	1	0	0	IHC	[91]
Piwil2	1	1	1	1	0	0	NA	NA	qPCR	[3]
Piwil2	0	0	1	1	0	0	0	0	IHC	[92]
Mov10l1	1	1	1	1	1	1	NA	NA	qPCR	[3]
Mov10l1	1	1	1	1	0	0	0	0	In situ	[93]
Tdrd1	1	1	1	1	1	1	NA	NA	qPCR	[3]
Tdrd1	0	0	1	1	0	0	0	0	In situ	[94]

Tdrd1	0	0	1	1	1	1	0	0	IHC	[94]
Ccna1	0	0	1	1	1	1	NA	NA	qPCR	[3]
Ccna1	0	0	0	1	1	0	0	0	In situ	[95]
Ldhc	0	0	1	1	1	1	NA	NA	qPCR	[3]
Ldhc	0	0	0	1	1	1	0	0	IHC	[96]
Mak	0	0	1	1	1	1	NA	NA	qPCR	[3]
Meig1	0	0	1	1	1	1	NA	NA	qPCR	[3]
Meig1	0	0	0	1	1	0	0	0	IHC	[97]
Pdha2	0	0	1	1	1	1	NA	NA	qPCR	[3]
Spa17	0	0	1	1	1	1	NA	NA	qPCR	[3]
Adad1	0	0	1	1	1	1	NA	NA	qPCR	[3]
Tesk1	0	0	1	1	1	1	NA	NA	qPCR	[3]
Adam2	0	0	1	1	1	1	NA	NA	qPCR	[3]
Hist1h1t	0	0	1	1	1	1	NA	NA	qPCR	[3]
Hist1h1t	0	0	1	1	0	0	0	0	In situ	[98]
Hist1h1t	0	0	1	1	1	1	0	0	IHC	[98]
Smcp	0	0	1	1	1	1	NA	NA	qPCR	[3]

Pabpc2	0	0	1	1	1	1	NA	NA	qPCR	[3]
Gapdhs	0	0	1	1	1	1	NA	NA	qPCR	[3]
Gykl1	0	0	1	1	1	1	NA	NA	qPCR	[3]
Gk2	0	0	1	1	1	1	NA	NA	qPCR	[3]
Zfa-ps	0	0	1	1	1	1	NA	NA	qPCR	[3]
Pou5f2	0	0	0	1	0	0	0	0	In situ	[99]
Ovol1	0	0	1	1	1	0	0	0	In situ	[100]
Brdt	0	0	1	1	1	1	0	0	In situ	[101]
Brdt	0	0	1	1	1	1	0	0	Reporter	[102]
Ddx25	0	0	1	1	1	0	0	1	IHC	[103] [rat; different protein isoform in Sertoli cells]
Ybx2	0	0	1	1	1	0	NA	NA	Northern	[104]
Khdrbs1	0	0	1	1	1	0	1	0	IHC	[105]
Dazap1	1	1	1	0	0	0	1	0	In situ	[106]
Dazap1	0	0	0	1	1	1	0	0	IHC	[106]
Wt1	0	0	0	0	0	0	1	0	In situ	[107]
Rhox5	0	0	0	0	0	0	1	0	In situ	[108]

Sox8	0	0	0	0	0	0	1	0	RNASeq	[109]
Rexo1	0	0	1	1	0	0	0	0	In situ	[110]
Rexo1	0	0	1	1	0	0	0	0	IHC	[111] [human]
Nek2	1	1	1	1	0	0	0	0	In situ	[112]
Nek2	0	0	1	1	1	0	0	0	IHC	[113]
Meioc	0	0	1	1	0	0	0	0	IHC	[114]
Ezh1	0	0	1	1	0	0	0	0	In situ	[115]
Cd9	1	1	1	1	1	0	0	0	In situ	[116]
Cd9	1	1	1	1	1	1	0	0	IHC	[117]
Bclb6b	1	0	0	0	1	0	0	0	IHC	[5]
Lhx1	1	0	0	0	1	0	0	0	IHC	[5]
Sufu	0	0	0	0	0	1	0	0	IHC	[118] [rat]
Ccna2	1	1	1	0	0	0	0	0	In situ	[119]
Ccna2	1	1	1	0	0	0	0	0	IHC	[119]
Ccne1	0	0	0	1	0	0	0	1	qPCR	[120]
Ccne1	0	0	0	1	0	0	0	1	IHC	[120]
Ccne2	0	1	1	1	0	0	0	0	qPCR	[120]

Ccne2	0	1	1	1	0	0	0	0	IHC	[120]
Ccnb1	0	0	1	1	1	1	0	0	In situ	[121]
Ccnb2	0	0	0	1	0	1	0	0	In situ	[122]
Ccnd1	1	1	0	0	0	0	0	1	IHC	[123]
Ccnd3	1	1	1	1	1	1	1	1	IHC	[123]
Ngn3	1	0	0	0	0	0	0	0	In situ	[124]
Smad1	1	1	1	1	0	0	1	0	In situ	[125]
Smad6	0	1	1	1	0	0	1	0	In situ	[125]
Smad6	0	1	1	1	0	0	1	0	IHC	[125]
Crabp1	1	1	0	0	0	0	0	0	IHC	[126] [rat]
Crabp1	1	1	0	0	0	0	0	0	Northern	[126] [rat]
Esrp1	1	1	0	0	0	0	0	0	qPCR	[127]
Esrp1	1	1	1	1	1	1	0	0	IHC	[127]
Tob1	0	0	1	1	1	0	0	0	In situ	[128]
Tob1	0	0	1	1	1	0	0	0	IHC	[128]
Kitl	0	0	0	0	0	0	1	0	In situ	[129] [Sertoli cells until P9 are positive, no signal in adult]

Kitl	0	0	0	0	0	0	1	0	IHC	[129] [Sertoli cells until P9 are positive, no signal in adult]
Itga6	1	NA	NA	NA	NA	NA	NA	NA	Transplantation	[130]
Itga6	1	1	1	1	1	1	1	0	IHC	[131]
Itgb1	1	NA	NA	NA	NA	NA	NA	NA	Transplantation	[130]
Itgb1	1	1	1	1	1	1	1	0	IHC	[131]
Adgra3	1	1	0	0	0	0	0	0	Reporter	[132]
Brca2	1	1	1	1	0	0	0	0	In situ	[133]
Pin1	1	1	1	1	1	1	0	1	IHC	[134]
Uchl1	1	0	0	0	0	0	0	0	IHC	[135]
Cdkn1c	0	1	1	0	0	0	0	1	IHC	[136]
Bmi1	1	0	0	0	0	0	0	0	In situ	[137]
Bmi1	1	0	0	0	0	0	0	0	IHC	[137]
Syce3	0	0	1	1	0	0	0	0	IHC	[138]
Foxj1	0	0	1	1	0	0	0	0	IHC	[139]
Piwil1	0	0	0	1	1	1	0	0	IHC	[92]
Taf7	1	1	1	1	0	0	0	0	IHC	[36]

Taf3	1	1	1	1	0	0	0	0	IHC	[36]
Tbp	1	1	1	1	1	1	0	0	IHC	[36]
1700013H16Rik	0	0	1	1	0	0	0	0	IHC	[140]
Trim27	0	0	1	1	1	1	1	0	IHC	[141]
Chd5	0	0	0	0	1	1	0	0	IHC	[142]
Zmynd15	0	0	1	1	1	1	0	0	In situ	[143]
Zmynd15	0	0	0	0	1	1	0	0	IHC	[143]
Prss42	0	0	1	1	0	0	0	0	In situ	[144]
Prss42	0	0	0	0	1	1	0	0	IHC	[144]
Prss43	0	0	1	1	0	0	0	0	In situ	[144]
Prss43	0	0	1	1	1	1	0	0	IHC	[144]
Prss44	0	0	1	1	0	0	0	0	In situ	[144]
Dyrk1b	0	0	0	1	1	1	0	0	In situ	[56]
Dyrk2	0	0	0	0	1	1	0	0	In situ	[56]
Dyrk3	0	0	0	1	1	1	0	0	In situ	[56]
Syce1	0	0	1	1	0	0	0	0	IHC	[145]
Per1	0	1	0	0	0	1	0	0	IHC	[146]

Clock	0	0	0	0	1	1	0	0	IHC	[146]
Akap4	0	0	0	0	1	0	0	0	In situ	[147] [human]<br>[148] [mouse]
Gata1	0	0	0	0	0	0	1	0	IHC	[149]
Smc1b	0	0	1	1	0	0	0	0	IHC	[150]
Tex40	0	0	0	0	1	1	0	0	In situ	[151]
H1fnt	0	0	0	0	1	1	0	0	qPCR	[152]
H1fnt	0	0	0	0	1	1	0	0	IHC	[152]
1700024P04Rik	0	0	0	0	0	1	0	0	Western	[153]
Top2a	1	1	1	1	0	0	0	0	IHC	[154]
Bcl2l11	1	1	1	1	1	1	1	0	In situ	[155]
Phf5a	0	0	1	1	0	0	0	0	IHC	[156]
U2af1	0	0	1	1	1	1	0	0	IHC	[156]
Hsd3b6	0	0	0	0	0	0	0	1	In situ	[157]
Hsd17b11	0	0	0	0	0	0	0	1	In situ	[158]
Cyp17a1	0	0	0	0	0	0	0	1	IHC	[159] [rat]
Vcam1	0	0	0	0	0	0	0	1	IHC	[160]

Meiob	0	0	1	0	0	0	0	0	In situ	[161], [151]
Ccnd2	1	1	1	1	1	1	1	0	IHC	[123]
Dll1	0	0	0	0	0	1	0	0	IHC	[45]
Slx	0	0	1	1	1	1	NA	NA	qPCR	[3]
Slx	0	0	0	0	1	0	0	0	In situ	[162]
Slx	0	0	0	0	1	0	0	0	IHC	[162]
Nos2	1	0	0	0	0	0	0	0	IHC	[52]
Gfra1	1	0	0	0	0	0	0	0	In situ	[163]
Hist1h4i	0	0	1	1	0	0	0	0	In situ	[26]
Poteg	0	0	1	1	0	0	0	0	In situ	[164]
Bex1	0	0	1	1	1	1	0	0	In situ	[165] [5 week old mice]
Tfcp2	0	0	0	0	0	0	1	0	In situ	[166]
Uba1y	1	1	1	0	1	1	NA	NA	qPCR	[3]
Usp9y	1	1	1	0	1	1	NA	NA	qPCR	[3]
Tex16	1	1	1	1	1	1	NA	NA	qPCR	[3]
Pramel3	1	1	1	1	1	1	NA	NA	qPCR	[3]
Magea5	1	1	1	1	1	1	NA	NA	qPCR	[3]
Lin28a	1	1	0	0	0	0	NA	NA	qPCR	[3]

Lin28a	1	0	0	0	0	0	0	0	IHC	[167]
Pramel1	1	1	1	1	0	0	NA	NA	qPCR	[3]
Pramel1	0	0	0	0	1	1	0	0	IHC	[168]
Figla	1	1	0	0	0	0	NA	NA	qPCR	[3]
Id1	0	0	0	1	1	1	0	0	IHC	[8]
Id2	0	0	0	1	1	0	1	0	IHC	[8]
Id3	0	0	0	0	0	0	1	0	IHC	[8]
Ptch1	0	0	1	1	0	0	0	0	IHC	[118] [rat]
Ccnb3	0	0	1	1	1	1	0	0	In situ	[169]
Utf1	1	1	0	0	0	0	0	0	IHC	[111]
Smad5	1	1	1	1	1	0	1	0	In situ	[125]
Smad9	0	0	1	1	1	1	0	0	In situ	[125]
Smad4	1	1	1	1	1	0	1	0	In situ	[125]
Smad7	0	1	1	1	0	0	1	0	In situ	[125]
Brca1	1	1	1	1	0	0	0	0	In situ	[133]
Ret	1	0	0	0	0	0	0	0	IHC	[170]
Csf1	0	0	0	0	0	0	0	1	Reporter	[171]

Zfp59	0	0	0	0	1	1	0	0	Northern	[172]
Zfp59	0	0	0	0	1	1	0	0	IHC	[172]
Spata22	0	0	1	1	0	0	0	0	IHC	[173] [rat]
Notch1	1	1	0	0	1	1	0	0	IHC	[45]
Notch2	0	0	1	1	1	1	0	0	IHC	[45]
Notch3	1	1	1	1	1	1	0	0	IHC	[45]
Dll4	0	0	1	1	1	1	1	0	IHC	[45]

Marker genes not annotated in the present dataset:

Hist1h4m	1	1	0	0	0	0	0	0	In situ	[26]
Rbmy	1	1	1	0	1		NA	NA	qPCR	[3]
Fthl17	1	1	0	0	0	0	NA	NA	qPCR	[3]
Tex13	1	1	1	0	1	1	NA	NA	qPCR	[3]
Ott	1	1	1	1	1	1	NA	NA	qPCR	[3]
Tex18	1	1	1	1	0	0	NA	NA	qPCR	[3]
Gli1	1	1	1	1	0	1	0	0	IHC	[118] [rat]
Cdh1	1	0	0	0	0	0	0	0	IHC	[174]
Rad21l	0	0	1	1	0	0	0	0	IHC	[175]

1 Costoya, J. A. *et al.* Essential role of Plzf in maintenance of spermatogonial stem cells. *Nature genetics* **36**, 653 (2004).
2 Buaas, F. W. *et al.* Plzf is required in adult male germ cells for stem cell self-renewal. *Nature genetics* **36**, 647 (2004).
3 Wang, P. J., Page, D. C. & McCarrey, J. R. Differential expression of sex-linked and autosomal germ-cell-specific genes during spermatogenesis in the mouse. *Human molecular genetics* **14**, 2911-2918 (2005).
4 Nair, M. *et al.* Nuclear regulator Pygo2 controls spermiogenesis and histone H3 acetylation. *Developmental biology* **320**, 446-455 (2008).
5 Oatley, J. M., Avarbock, M. R. & Brinster, R. L. Glial cell line-derived neurotrophic factor regulation of genes essential for self-renewal of mouse spermatogonial stem cells is dependent on Src family kinase signaling. *Journal of Biological Chemistry* **282**, 25842-25851 (2007).
6 Igakura, T. *et al.* A null mutation in basigin, an immunoglobulin superfamily member, indicates its important roles in peri-implantation development and spermatogenesis. *Developmental biology* **194**, 152-165 (1998).
7 Wojtasz, L. *et al.* Mouse HORMAD1 and HORMAD2, two conserved meiotic chromosomal proteins, are depleted from synapsed chromosome axes with the help of TRIP13 AAA-ATPase. *PLoS genetics* **5**, e1000702 (2009).
8 Sablitzky, F. *et al.* Stage-and subcellular-specific expression of Id proteins in male germ and Sertoli cells implicates distinctive regulatory roles for Id proteins during meiosis, spermatogenesis, and Sertoli cell function. *Development* **29**, 30 (1998).
9 Oatley, M. J., Kaucher, A. V., Racicot, K. E. & Oatley, J. M. Inhibitor of DNA binding 4 is expressed selectively by single spermatogonia in the male germline and regulates the self-renewal of spermatogonial stem cells in mice. *Biology of reproduction* **85**, 347-356 (2011).
10 Coultas, L. *et al.* Concomitant loss of proapoptotic BH3-only Bcl-2 antagonists Bik and Bim arrests spermatogenesis. *The EMBO journal* **24**, 3963-3973 (2005).
11 Niederberger, C., Agulnik, A. I., Cho, Y., Lamb, D. & Bishop, C. E. In situ hybridization shows that Dazla expression in mouse testis is restricted to premeiotic stages IV-VI of spermatogenesis. *Mammalian genome* **8**, 277-278 (1997).
12 Ruggiu, M. *et al.* The mouse Dazla gene encodes a cytoplasmic protein essential for gametogenesis. *Nature* **389**, 73 (1997).
13 Celebi, C. *et al.* Tex 19 paralogs exhibit a gonad and placenta-specific expression in the mouse. *Journal of Reproduction and Development* **58**, 360-365 (2012).
14 Yamamoto, A. *et al.* Cell cycle-dependent expression of the mouseRad51 gene in proliferating cells. *Molecular and General Genetics MGG* **251**, 1-12 (1996).
15 Oulad-Abdelghani, M. *et al.* Characterization of a premeiotic germ cell-specific cytoplasmic protein encoded by Stra8, a novel retinoic acid-responsive gene. *The Journal of cell biology* **135**, 469-477 (1996).
16 Raverot, G., Weiss, J., Park, S. Y., Hurley, L. & Jameson, J. L. Sox3 expression in undifferentiated spermatogonia is required for the progression of spermatogenesis. *Developmental biology* **283**, 215-225 (2005).
17 Bramlage, B., Kosciessa, U. & Doenecke, D. Differential expression of the murine histone genes H3. 3A and H3. 3B. *Differentiation* **62**, 13-20 (1997).
18 Delmas, V., Van der Hoorn, F., Mellström, B., Jegou, B. & Sassone-Corsi, P. Induction of CREM activator proteins in spermatids: down-stream targets and implications for haploid germ cell differentiation. *Molecular endocrinology* **7**, 1502-1514 (1993).
19 Luo, M. *et al.* Polycomb protein SCML2 associates with USP7 and counteracts histone H2A ubiquitination in the XY chromatin during male meiosis. *PLoS genetics* **11**, e1004954 (2015).

20 Tanaka, H. *et al.* Identification and characterization of a haploid germ cell-specific nuclear ProteinKinase (haspin) in spermatid nuclei and its effects on somatic cells. *Journal of Biological Chemistry* **274**, 17049-17057 (1999).

21 Takabayashi, S., Yamauchi, Y., Tsume, M., Noguchi, M. & Katoh, H. A spontaneous smc1b mutation causes cohesin protein dysfunction and sterility in mice. *Experimental Biology and Medicine* **234**, 994-1001 (2009).

22 Liu, Z. *et al.* Jmjd1a demethylase-regulated histone modification is essential for cAMP-response element modulator-regulated gene expression and spermatogenesis. *Journal of Biological Chemistry* **285**, 2758-2770 (2010).

23 Okada, Y., Scott, G., Ray, M. K., Mishina, Y. & Zhang, Y. Histone demethylase JHDM2A is critical for Tnp1 and Prm1 transcription and spermatogenesis. *Nature* **450**, 119 (2007).

24 Shannon, M., Richardson, L., Christian, A., Handel, M. A. & Thelen, M. P. Differential gene expression of mammalian SPO11/TOP6A homologs during meiosis. *FEBS letters* **462**, 329-334 (1999).

25 Rabini, S. *et al.* Spermatogenesis in mice is not affected by histone H1. 1 deficiency. *Experimental cell research* **255**, 114-124 (2000).

26 Sun, R. & Qi, H. Dynamic expression of combinatorial replication-dependent histone variant genes during mouse spermatogenesis. *Gene Expression Patterns* **14**, 30-41 (2014).

27 Fu, X.-F. *et al.* DAZ family proteins, key players for germ cell development. *International journal of biological sciences* **11**, 1226 (2015).

28 Xu, E. Y., Moore, F. L. & Pera, R. A. R. A gene family required for human germ cell development evolved from an ancient meiotic gene conserved in metazoans. *Proceedings of the National Academy of Sciences* **98**, 7414-7419 (2001).

29 Chapman, D. L. & Wolgemuth, D. J. Expression of proliferating cell nuclear antigen in the mouse germ line and surrounding somatic cells suggests both proliferation-dependent and-independent modes of function. *International Journal of Developmental Biology* **38**, 491-497 (1994).

30 Watanabe, D. *et al.* Molecular cloning of a novel Ca (2+)-binding protein (calmegin) specifically expressed during male meiotic germ cell development. *Journal of Biological Chemistry* **269**, 7744-7749 (1994).

31 Pezzi, N. *et al.* STAG3, a novel gene encoding a protein involved in meiotic chromosome pairing and location of STAG3-related genes flanking the Williams-Beuren syndrome deletion. *The FASEB Journal* **14**, 581-592 (2000).

32 Adelman, C. A. & Petrini, J. H. ZIP4H (TEX11) deficiency in the mouse impairs meiotic double strand break repair and the regulation of crossing over. *PLoS genetics* **4**, e1000042 (2008).

33 Eijpe, M., Offenberg, H., Jessberger, R., Revenkova, E. & Heyting, C. Meiotic cohesin REC8 marks the axial elements of rat synaptonemal complexes before cohesins SMC1β and SMC3. *The Journal of cell biology* **160**, 657-670 (2003).

34 Lee, J., Yokota, T. & Yamashita, M. Analyses of mRNA expression patterns of cohesin subunits Rad21 and Rec8 in mice: Germ cell-specific expression of rec8 mRNA in both male and female mice. *Zoological science* **19**, 539-544 (2002).

35 Osuru, H. P. *et al.* The acrosomal protein SP-10 (Acrv1) is an ideal marker for staging of the cycle of seminiferous epithelium in the mouse. *Molecular reproduction and development* **81**, 896-907 (2014).

36 Pointud, J.-C. *et al.* The intracellular localisation of TAF7L, a paralogue of transcription factor TFIID subunit TAF7, is developmentally regulated during male germ-cell differentiation. *Journal of cell science* **116**, 1847-1858 (2003).

37 Zhuang, X.-j. *et al.* SLXL1, a novel acrosomal protein, interacts with DKKL1 and is involved in fertilization in mice. *PloS one* **6**, e20866 (2011).

38 Decarpentrie, F. *et al.* Human and mouse ZFY genes produce a conserved testis-specific transcript encoding a zinc finger protein with a short acidic domain and modified transactivation potential. *Human molecular genetics* **21**, 2631-2645 (2012).
39 Toyoda, S. *et al.* Sohlh2 affects differentiation of KIT positive oocytes and spermatogonia. *Developmental biology* **325**, 238-248 (2009).
40 Ryu, B.-Y., Orwig, K. E., Kubota, H., Avarbock, M. R. & Brinster, R. L. Phenotypic and functional characteristics of spermatogonial stem cells in rats. *Developmental biology* **274**, 158-170 (2004).
41 Wang, P. J. & Pan, J. The role of spermatogonially expressed germ cell-specific genes in mammalian meiosis. *Chromosome research* **15**, 623-632 (2007).
42 Habu, T., Taki, T., West, A., Nishimune, Y. & Morita, T. The mouse and human homologs of DMC1, the yeast meiosis-specific homologous recombination gene, have a common unique form of exon-skipped transcript in meiosis. *Nucleic acids research* **24**, 470-477 (1996).
43 Yoshida, K. *et al.* The mouse RecA-like gene Dmc1 is required for homologous chromosome synapsis during meiosis. *Molecular cell* **1**, 707-718 (1998).
44 Heidaran, M. A., Showman, R. M. & Kistler, W. S. A cytochemical study of the transcriptional and translational regulation of nuclear transition protein 1 (TP1), a major chromosomal protein of mammalian spermatids. *The Journal of cell biology* **106**, 1427-1433 (1988).
45 Murta, D. *et al.* Dynamics of Notch pathway expression during mouse testis post-natal development and along the spermatogenic cycle. *PloS one* **8**, e72767 (2013).
46 Caldwell, K. A. & Handel, M. A. Protamine transcript sharing among postmeiotic spermatids. *Proceedings of the National Academy of Sciences* **88**, 2407-2411 (1991).
47 Shih, D. & Kleene, K. A study by in situ hybridization of the stage of appearance and disappearance of the transition protein 2 and the mitochondrial capsule seleno-protein mrnas during spermatogenesis in the mouse. *Molecular reproduction and development* **33**, 222-227 (1992).
48 Ballow, D., Meistrich, M., Matzuk, M. & Rajkovic, A. Sohlh1 is essential for spermatogonial differentiation. *Developmental biology* **294**, 161-167 (2006).
49 Goto, M. *et al.* Transcription switch of two phosphoglycerate kinase genes during spermatogenesis as determined with mouse testis sections in situ. *Experimental cell research* **186**, 273-278 (1990).
50 Cocquet, J. *et al.* The multicopy gene Sly represses the sex chromosomes in the male mouse germline after meiosis. *PLoS biology* **7**, e1000244 (2009).
51 Reynard, L. N., Cocquet, J. & Burgoyne, P. S. The multi-copy mouse gene Sycp3-like Y-linked (Sly) encodes an abundant spermatid protein that interacts with a histone acetyltransferase and an acrosomal protein. *Biology of reproduction* **81**, 250-257 (2009).
52 Suzuki, H., Sada, A., Yoshida, S. & Saga, Y. The heterogeneity of spermatogonia is revealed by their topology and expression of marker proteins including the germ cell-specific proteins Nanos2 and Nanos3. *Developmental biology* **336**, 222-231 (2009).
53 Iguchi, N., Tanaka, H., Yomogida, K. & Nishimune, Y. Isolation and characterization of a novel cDNA encoding a DNA-binding protein (Hils1) specifically expressed in testicular haploid germ cells. *International journal of andrology* **26**, 354-365 (2003).
54 Manova, K., Nocka, K., Besmer, P. & Bachvarova, R. F. Gonadal expression of c-kit encoded at the W locus of the mouse. *Development* **110**, 1057-1069 (1990).
55 Falender, A. E. *et al.* Maintenance of spermatogenesis requires TAF4b, a gonad-specific subunit of TFIID. *Genes & development* **19**, 794-803 (2005).
56 Sacher, F., Möller, C., Bone, W., Gottwald, U. & Fritsch, M. The expression of the testis-specific Dyrk4 kinase is highly restricted to step 8 spermatids but is not required for male fertility in mice. *Molecular and cellular endocrinology* **267**, 80-88 (2007).

57 Pesce, M., Wang, X., Wolgemuth, D. J. & Schöler, H. R. Differential expression of the Oct-4 transcription factor during mouse germ cell differentiation. *Mechanisms of development* **71**, 89-98 (1998).

58 Ohmura, M. *et al.* Spatial analysis of germ stem cell development in Oct-4/EGFP transgenic mice. *Archives of histology and cytology* **67**, 285-296 (2004).

59 Wu, J. Y. & Means, A. R. Ca2+/calmodulin-dependent protein kinase IV is expressed in spermatids and targeted to chromatin and the nuclear matrix. *Journal of Biological Chemistry* **275**, 7994-7999 (2000).

60 Rotgers, E., Nurmio, M., Pietilä, E., Cisneros-Montalvo, S. & Toppari, J. E2F1 controls germ cell apoptosis during the first wave of spermatogenesis. *Andrology* **3**, 1000-1014 (2015).

61 Garcia-Fabiani, M. B. *et al.* Methylation of the Gpat2 promoter regulates transient expression during mouse spermatogenesis. *Biochemical Journal* **471**, 211-220 (2015).

62 Oh, S. D. *et al.* The novel, actin-like protein Tact3 is expressed in rodent testicular haploid germ cells. *Molecular reproduction and development* **80**, 988-999 (2013).

63 Lim, J.-H. *et al.* Crlz-1 is prominently expressed in spermatogonia and Sertoli cells during early testis development and in spermatids during late spermatogenesis. *Journal of Histochemistry & Cytochemistry* **61**, 522-528 (2013).

64 Sarraj, M. *et al.* Expression of Wsb2 in the developing and adult mouse testis. *Reproduction* **133**, 753-761 (2007).

65 Saito, H. *et al.* Germ cell-specific expression of microphthalmia-associated transcription factor mRNA in mouse testis. *Journal of biochemistry* **134**, 143-150 (2003).

66 Yan, W., Burns, K. H., Ma, L. & Matzuk, M. M. Identification of Zfp393, a germ cell-specific gene encoding a novel zinc finger protein. *Mechanisms of development* **118**, 233-239 (2002).

67 Santucci-Darmanin, S., Vidal, F., Scimeca, J. C., Turc-Carel, C. & Paquis-Flucklinger, V. Family of SRY/Sox proteins is involved in the regulation of the mouse Msh4 (MutS Homolog 4) gene expression. *Molecular reproduction and development* **60**, 172-180 (2001).

68 Ohuchi, J. *et al.* Characterization of a novel gene, sperm-tail-associated protein (Stap), in mouse post-meiotic testicular germ cells. *Molecular reproduction and development* **59**, 350-358 (2001).

69 Wagner, K., Mincheva, A., Korn, B., Lichter, P. & Pöpperl, H. Pbx4, a new Pbx family member on mouse chromosome 8, is expressed during spermatogenesis. *Mechanisms of development* **103**, 127-131 (2001).

70 Hsu, S.-H., Shyu, H.-W., Hsieh-Li, H.-M. & Li, H. Spz1, a novel bHLH-Zip protein, is specifically expressed in testis. *Mechanisms of development* **100**, 177-187 (2001).

71 Ito, S. *et al.* A novel WD40 repeat protein, WDC146, highly expressed during spermatogenesis in a stage-specific manner. *Biochemical and biophysical research communications* **280**, 656-663 (2001).

72 Inoue, A. *et al.* The transcript for a novel protein with a zinc finger motif is expressed at specific stages of mouse spermatogenesis. *Biochemical and biophysical research communications* **273**, 398-403 (2000).

73 Fujii, T. *et al.* Sperizin is a murine RING zinc-finger protein specifically expressed in haploid germ cells. *Genomics* **57**, 94-101 (1999).

74 Tsunekawa, N., Matsumoto, M., Tone, S., Nishida, T. & Fujimoto, H. The Hsp70 homolog gene, Hsc70t, is expressed under translational control during mouse spermiogenesis. *Molecular reproduction and development* **52**, 383-391 (1999).

75 Takahashi, H., Koshimizu, U. & Nakamura, T. A novel transcript encoding truncated LIM kinase 2 is specifically expressed in male germ cells undergoing meiosis. *Biochemical and biophysical research communications* **249**, 138-145 (1998).

76 Bauer, U.-M., Schneider-Hirsch, S., Reinhardt, S., Benavente, R. & Maelicke, A. The murine nuclear orphan receptor GCNF is expressed in the XY body of primary spermatocytes. *FEBS letters* **439**, 208-214 (1998).

77 Branford, W. W. *et al.* Spx1, a novel X-linked homeobox gene expressed during spermatogenesis. *Mechanisms of development* **65**, 87-98 (1997).

78 Herrada, G. & Wolgemuth, D. J. The mouse transcription factor Stat4 is expressed in haploid male germ cells and is present in the perinuclear theca of spermatozoa. *Journal of cell science* **110**, 1543-1553 (1997).

79 Dix, D. J. *et al.* Developmentally Regulated Expression ofHsp70-2and aHsp70-2/lacZTransgene during Spermatogenesis. *Developmental biology* **174**, 310-321 (1996).

80 Kondo, M. *et al.* Genomic sequence analysis of the bovine male-enhanced antigen-1 (Mea-1) and differential localization of its transcripts and products during spermatogenesis. *DNA Sequence* **6**, 75-85 (1996).

81 Sarge, K. D., Park-Sarge, O.-K., Kirby, J. D., Mayo, K. E. & Morimoto, R. I. Expression of heat shock factor 2 in mouse testis: potential role as a regulator of heat-shock protein gene expression during spermatogenesis. *Biology of reproduction* **50**, 1334-1343 (1994).

82 Mizuki, N., Sarapata, D. E., Garcia-Sanz, J. A. & Kasahara, M. The mouse male germ cell-specific gene Tpx-1: molecular structure, mode of expression in spermatogenesis, and sequence similarity to two non-mammalian genes. *Mammalian Genome* **3**, 274-280 (1992).

83 Keshet, E., Itin, A., Fischman, K. & Nir, U. The testis-specific transcript (ferT) of the tyrosine kinase FER is expressed during spermatogenesis in a stage-specific manner. *Molecular and Cellular Biology* **10**, 5021-5025 (1990).

84 Cunliffe, V., Koopman, P., McLaren, A. & Trowsdale, J. A mouse zinc finger gene which is transiently expressed during spermatogenesis. *The EMBO journal* **9**, 197-205 (1990).

85 Sage, J. *et al.* Temporal and spatial control of the Sycp1 gene transcription in the mouse meiosis: regulatory elements active in the male are not sufficient for expression in the female gonad. *Mechanisms of development* **80**, 29-39 (1999).

86 Bao, J. *et al.* STK31 (TDRD8) is dynamically regulated throughout mouse spermatogenesis and interacts with MIWI protein. *Histochemistry and cell biology* **137**, 377-389 (2012).

87 Hamer, G. *et al.* Characterization of a novel meiosis-specific protein within the central element of the synaptonemal complex. *Journal of cell science* **119**, 4025-4032 (2006).

88 Wu, M.-H. *et al.* Sequence and expression of testis-expressed gene 14 (Tex14): a gene encoding a protein kinase preferentially expressed during spermatogenesis. *Gene expression patterns* **3**, 231-236 (2003).

89 Greenbaum, M. P. *et al.* TEX14 is essential for intercellular bridges and fertility in male mice. *Proceedings of the National Academy of Sciences of the United States of America* **103**, 4982-4987 (2006).

90 Fujiwara, Y. *et al.* Isolation of a DEAD-family protein gene that encodes a murine homolog of Drosophila vasa and its specific expression in germ cell lineage. *Proceedings of the National Academy of Sciences* **91**, 12258-12262 (1994).

91 Pan, J. *et al.* RNF17, a component of the mammalian germ cell nuage, is essential for spermiogenesis. *Development* **132**, 4029-4039 (2005).

92 Kuramochi-Miyagawa, S. *et al.* Mili, a mammalian member of piwi family gene, is essential for spermatogenesis. *Development* **131**, 839-849 (2004).

93 Frost, R. J. *et al.* MOV10L1 is necessary for protection of spermatocytes against retrotransposons by Piwi-interacting RNAs. *Proceedings of the National Academy of Sciences* **107**, 11847-11852 (2010).

94 Chuma, S. *et al.* Mouse Tudor Repeat-1 (MTR-1) is a novel component of chromatoid bodies/nuages in male germ cells and forms a complex with snRNPs. *Mechanisms of development* **120**, 979-990 (2003).

95 Sweeney, C. *et al.* A distinct cyclin A is expressed in germ cells in the mouse. *Development* **122**, 53-64 (1996).

96 Hintz, M. & Goldberg, E. Immunohistochemical localization of LDH-X during spermatogenesis in mouse testes. *Developmental biology* **57**, 375-384 (1977).

97 Watanabe, D., Sawada, K., Koshimizu, U., Kagawa, T. & Nishimune, Y. Characterization of male meiotic germ cell-specific antigen (Meg 1) by monoclonal antibody TRA 369 in mice. *Molecular reproduction and development* **33**, 307-312 (1992).

98 Drabent, B., Bode, C., Bramlage, B. & Doenecke, D. Expression of the mouse testicular histone gene H1t during spermatogenesis. *Histochemistry and cell biology* **106**, 247-251 (1996).

99 Andersen, B. *et al.* Sperm 1: a POU-domain gene transiently expressed immediately before meiosis I in the male germ cell. *Proceedings of the National Academy of Sciences* **90**, 11084-11088 (1993).

100 Li, B. *et al.* Ovol1 regulates meiotic pachytene progression during spermatogenesis by repressing Id2 expression. *Development* **132**, 1463-1473 (2005).

101 Shang, E. *et al.* Identification of unique, differentiation stage-specific patterns of expression of the bromodomain-containing genes Brd2, Brd3, Brd4, and Brdt in the mouse testis. *Gene expression patterns* **4**, 513-519 (2004).

102 Gaucher, J. *et al.* Bromodomain-dependent stage-specific male genome programming by Brdt. *The EMBO journal* **31**, 3809-3820 (2012).

103 Sheng, Y., Tsai-Morris, C.-H. & Dufau, M. L. Cell-specific and hormone-regulated expression of gonadotropin-regulated testicular RNA helicase gene (GRTH/Ddx25) resulting from alternative utilization of translation initiation codons in the rat testis. *Journal of Biological Chemistry* **278**, 27796-27803 (2003).

104 Gu, W. *et al.* Mammalian male and female germ cells express a germ cell-specific Y-Box protein, MSY2. *Biology of reproduction* **59**, 1266-1274 (1998).

105 Paronetto, M. P. *et al.* The nuclear RNA-binding protein Sam68 translocates to the cytoplasm and associates with the polysomes in mouse spermatocytes. *Molecular biology of the cell* **17**, 14-24 (2006).

106 Vera, Y. *et al.* Deleted in azoospermia associated protein 1 shuttles between nucleus and cytoplasm during normal germ cell maturation. *Journal of andrology* **23**, 622-628 (2002).

107 Pelletier, J. *et al.* Expression of the Wilms' tumor gene WT1 in the murine urogenital system. *Genes & development* **5**, 1345-1356 (1991).

108 Lindsey, J. S. & Wilkinson, M. F. Pem: a testosterone-and LH-regulated homeobox gene expressed in mouse Sertoli cells and epididymis. *Developmental biology* **179**, 471-484 (1996).

109 Roumaud, P., Hache, J. & Martin, L. J. Expression profiles of Sox transcription factors within the postnatal rodent testes. *Molecular and cellular biochemistry*, doi:10.1007/s11010-018-3302-3 (2018).

110 Rogers, M. B., Hosler, B. A. & Gudas, L. J. Specific expression of a retinoic acid-regulated, zinc-finger gene, Rex-1, in preimplantation embryos, trophoblast and spermatocytes. *Development* **113**, 815-824 (1991).

111 Kristensen, D. M. *et al.* Presumed pluripotency markers UTF-1 and REX-1 are expressed in human adult testes and germ cell neoplasms. *Human reproduction* **23**, 775-782 (2008).

112 Arama, E., Yanai, A., Kilfin, G., Bernstein, A. & Motro, B. Murine NIMA-related kinases are expressed in patterns suggesting distinct functions in gametogenesis and a role in the nervous system. *Oncogene* **16**, 1813 (1998).

113 Rhee, K. & Wolgemuth, D. J. The NIMA-related kinase 2, Nek2, is expressed in specific stages of the meiotic cell cycle and associates with meiotic chromosomes. *Development* **124**, 2167-2177 (1997).

114 Soh, Y. S. *et al.* Meioc maintains an extended meiotic prophase I in mice. *PLoS genetics* **13**, e1006704 (2017).

115 Mu, W., Starmer, J., Shibata, Y., Yee, D. & Magnuson, T. EZH1 in germ cells safeguards the function of PRC2 during spermatogenesis. *Developmental biology* **424**, 198-207 (2017).

116 Ito, C. *et al.* Tetraspanin family protein CD9 in the mouse sperm: unique localization, appearance, behavior and fate during fertilization. *Cell and tissue research* **340**, 583-594 (2010).

117 Kanatsu-Shinohara, M., Toyokuni, S. & Shinohara, T. CD9 is a surface marker on mouse and rat male germline stem cells. *Biology of reproduction* **70**, 70-75 (2004).

118 Mäkelä, J.-A. *et al.* Hedgehog signalling promotes germ cell survival in the rat testis. *Reproduction* **142**, 711-721 (2011).

119 Ravnik, S. E. & Wolgemuth, D. J. The Developmentally Restricted Pattern of Expression in the Male Germ Line of a MurineCyclin A, Cyclin A2, Suggests Roles in Both Mitotic and Meiotic Cell Cycles. *Developmental biology* **173**, 69-78 (1996).

120 Martinerie, L. *et al.* Mammalian E-type cyclins control chromosome pairing, telomere stability and CDK2 localization in male meiosis. *PLoS genetics* **10**, e1004165 (2014).

121 Chapman, D. L. & Wolgemuth, D. J. Identification of a mouse B-type cyclin which exhibits developmentally regulated expression in the germ line. *Molecular reproduction and development* **33**, 259-269 (1992).

122 Chapman, D. L. & Wolgemuth, D. J. Isolation of the murine cyclin B2 cDNA and characterization of the lineage and temporal specificity of expression of the B1 and B2 cyclins during oogenesis, spermatogenesis and early embryogenesis. *Development* **118**, 229-240 (1993).

123 Beumer, T. L., Roepers-Gajadien, H. L., Gademan, I. S., Kal, H. B. & de Rooij, D. G. Involvement of the D-type cyclins in germ cell proliferation and differentiation in the mouse. *Biology of reproduction* **63**, 1893-1898 (2000).

124 Yoshida, S. *et al.* Neurogenin3 delineates the earliest stages of spermatogenesis in the mouse testis. *Developmental biology* **269**, 447-458 (2004).

125 Itman, C. & Loveland, K. L. SMAD expression in the testis: an insight into BMP regulation of spermatogenesis. *Developmental dynamics* **237**, 97-111 (2008).

126 Zheng, W., Bucco, R. A., Schmitt, M. C., Wardlaw, S. A. & Ong, D. E. Localization of cellular retinoic acid-binding protein (CRABP) II and CRABP in developing rat testis. *Endocrinology* **137**, 5028-5035 (1996).

127 Saeidi, S. *et al.* Esrp1 is a marker of mouse fetal germ cells and differentially expressed during spermatogenesis. *PloS one* **13**, e0190925 (2018).

128 Shapouri, F. *et al.* Tob1 is expressed in developing and adult gonads and is associated with the P-body marker, Dcp2. *Cell and tissue research* **364**, 443-451 (2016).

129 Manova, K. *et al.* The expression pattern of the c-kit ligand in gonads of mice supports a role for the c-kit receptor in oocyte growth and in proliferation of spermatogonia. *Developmental biology* **157**, 85-99 (1993).

130 Shinohara, T., Avarbock, M. R. & Brinster, R. L. β1-and α6-integrin are surface markers on mouse spermatogonial stem cells. *Proceedings of the National Academy of Sciences* **96**, 5504-5509 (1999).

131 Sá, R., Miranda, C., Carvalho, F., Barros, A. & Sousa, M. Expression of stem cell markers: OCT4, KIT, ITGA6, and ITGB1 in the male germinal epithelium. *Systems biology in reproductive medicine* **59**, 233-243 (2013).

132 Seandel, M. *et al.* Generation of functional multipotent adult stem cells from GPR125+ germline progenitors. *Nature* **449**, 346 (2007).

133 Blackshear, P. E. *et al.* Brca1 and Brca2 expression patterns in mitotic and meiotic cells of mice. *Oncogene* **16**, 61 (1998).

134 Atchison, F. W. & Means, A. R. Spermatogonial depletion in adult Pin1-deficient mice. *Biology of reproduction* **69**, 1989-1997 (2003).

135 Luo, J., Megee, S. & Dobrinski, I. Asymmetric Distribution of UCH-L1 in spermatogonia is associated with maintenance and differentiation of spermatogonial stem cells. *Journal of cellular physiology* **220**, 460-468 (2009).

136 Kubota, H., Avarbock, M. R. & Brinster, R. L. Culture conditions and single growth factors affect fate determination of mouse spermatogonial stem cells. *Biology of reproduction* **71**, 722-731 (2004).

137 Zhang, S. *et al.* Expression localization of Bmi1 in mice testis. *Molecular and cellular endocrinology* **287**, 47-56 (2008).

138 Schramm, S. *et al.* A novel mouse synaptonemal complex protein is essential for loading of central element proteins, recombination, and fertility. *PLoS genetics* **7**, e1002088 (2011).

139 Miao, H., Miao, C. X., Li, N. & Han, J. FOXJ2 controls meiosis during spermatogenesis in male mice. *Molecular reproduction and development* **83**, 684-691 (2016).

140 Shi, Y.-Q. *et al.* SYCP3-like X-linked 2 is expressed in meiotic germ cells and interacts with synaptonemal complex central element protein 2 and histone acetyltransferase TIP60. *Gene* **527**, 352-359 (2013).

141 Zhuang, X.-J. *et al.* Trim27 interacts with Slx2, is associated with meiotic processes during spermatogenesis. *Cell Cycle* **15**, 2576-2584 (2016).

142 Li, W. *et al.* Chd5 orchestrates chromatin remodelling during sperm development. *Nature communications* **5**, 3812 (2014).

143 Yan, W. *et al.* Zmynd15 encodes a histone deacetylase-dependent transcriptional repressor essential for spermiogenesis and male fertility. *Journal of Biological Chemistry* **285**, 31418-31426 (2010).

144 Yoneda, R. *et al.* Three testis-specific paralogous serine proteases play different roles in murine spermatogenesis and are involved in germ cell survival during meiosis. *Biology of reproduction* **88** (2013).

145 Costa, Y. *et al.* Two novel proteins recruited by synaptonemal complex protein 1 (SYCP1) are at the centre of meiosis. *Journal of cell science* **118**, 2755-2762 (2005).

146 Alvarez, J., Chen, D., Storer, E. & Sehgal, A. Non-cyclic and developmental stage-specific expression of circadian clock proteins during murine spermatogenesis. *Biology of reproduction* **69**, 81-91 (2003).

147 Mohapatra, B., Verma, S., Shankar, S. & Suri, A. Molecular cloning of human testis mRNA specifically expressed in haploid germ cells, having structural homology with the A-kinase anchoring proteins. *Biochemical and biophysical research communications* **244**, 540-545 (1998).

148 Fulcher, K. D. *et al.* Characterization of Fscl cDNA for a mouse sperm fibrous sheath component. *Biology of reproduction* **52**, 41-49 (1995).

149 Yomogida, K. *et al.* Developmental stage-and spermatogenic cycle-specific expression of transcription factor GATA-1 in mouse Sertoli cells. *Development* **120**, 1759-1766 (1994).
150 Revenkova, E., Eijpe, M., Heyting, C., Gross, B. & Jessberger, R. Novel meiosis-specific isoform of mammalian SMC1. *Molecular and cellular biology* **21**, 6984-6998 (2001).
151 Kogo, H. *et al.* Screening of genes involved in chromosome segregation during meiosis I: toward the identification of genes responsible for infertility in humans. *Journal of human genetics* **55**, 293 (2010).
152 Tanaka, H. *et al.* HANP1/H1T2, a novel histone H1-like protein involved in nuclear formation and sperm fertility. *Molecular and cellular biology* **25**, 7107-7119 (2005).
153 Govin, J. *et al.* Pericentric heterochromatin reprogramming by new histone variants during mouse spermiogenesis. *J Cell Biol* **176**, 283-294 (2007).
154 Leduc, F., Maquennehan, V., Nkoma, G. B. & Boissonneault, G. DNA damage response during chromatin remodeling in elongating spermatids of mice. *Biology of reproduction* **78**, 324-332 (2008).
155 Meehan, T., Loveland, K., de Kretser, D. & Cory, S. Developmental regulation of the bcl-2 family during spermatogenesis: Insights into the sterility of bcl-w−/− male mice. *Cell death and differentiation* **8**, 225 (2001).
156 Rzymski, T., Grzmil, P., Meinhardt, A., Wolf, S. & Burfeind, P. PHF5A represents a bridge protein between splicing proteins and ATP-dependent helicases and is differentially expressed during mouse spermatogenesis. *Cytogenetic and genome research* **121**, 232-244 (2008).
157 Baker, P. J. *et al.* Expression of 3β-hydroxysteroid dehydrogenase type I and type VI isoforms in the mouse testis during development. *The FEBS Journal* **260**, 911-917 (1999).
158 O'Shaughnessy, P., Baker, P., Heikkila, M., Vainio, S. & McMahon, A. Localization of 17β-hydroxysteroid dehydrogenase/17-ketosteroid reductase isoform expression in the developing mouse testis—androstenedione is the major androgen secreted by fetal/neonatal Leydig cells. *Endocrinology* **141**, 2631-2637 (2000).
159 Le Goascogne, C. *et al.* Immunoreactive cytochrome P-45017α in rat and guineapig gonads, adrenal glands and brain. *Journal of reproduction and fertility* **93**, 609-622 (1991).
160 Sainio-Pöllänen, S. *et al.* CD106 (VCAM-1) in testicular immunoregulation. *Journal of reproductive immunology* **33**, 221-238 (1997).
161 Souquet, B. *et al.* MEIOB targets single-strand DNA and is necessary for meiotic recombination. *PLoS genetics* **9**, e1003784 (2013).
162 Reynard, L. N. *et al.* Expression analysis of the mouse multi-copy X-linked gene Xlr-related, meiosis-regulated (Xmr), reveals that Xmr encodes a spermatid-expressed cytoplasmic protein, SLX/XMR. *Biology of reproduction* **77**, 329-335 (2007).
163 Meng, X. *et al.* Regulation of cell fate decision of undifferentiated spermatogonia by GDNF. *Science* **287**, 1489-1493 (2000).
164 Wang, F., Hu, J., Song, P. & Gong, W. Two novel transcripts encoding two Ankyrin repeat containing proteins have preponderant expression during the mouse spermatogenesis. *Molecular biology reports* **34**, 249-260 (2007).
165 Yang, Q.-S. *et al.* Cloning and expression pattern of a spermatogenesis-related gene, BEX1, mapped to chromosome Xq22. *Biochemical genetics* **40**, 1-12 (2002).
166 Maguire, S., Millar, M., Sharpe, R. & Saunders, P. Stage-dependent expression of mRNA for cyclic protein 2 during spermatogenesis is modulated by elongate spermatids. *Molecular and cellular endocrinology* **94**, 79-88 (1993).

167 Zheng, K., Wu, X., Kaestner, K. H. & Wang, P. J. The pluripotency factor LIN28 marks undifferentiated spermatogonia in mouse. *BMC developmental biology* **9**, 38 (2009).

168 Mistry, B. V. *et al.* Differential expression of PRAMEL1, a cancer/testis antigen, during spermatogenesis in the mouse. *PloS one* **8**, e60611 (2013).

169 Nguyen, T. B. *et al.* Characterization and expression of mammalian cyclin b3, a prepachytene meiotic cyclin. *Journal of Biological Chemistry* **277**, 41960-41969 (2002).

170 Naughton, C. K., Jain, S., Strickland, A. M., Gupta, A. & Milbrandt, J. Glial cell-line derived neurotrophic factor-mediated RET signaling regulates spermatogonial stem cell fate. *Biology of reproduction* **74**, 314-321 (2006).

171 Ryan, G. R. *et al.* Rescue of the colony-stimulating factor 1 (CSF-1)–nullizygous mouse (Csf1 op/Csf1 op) phenotype with a CSF-1 transgene and identification of sites of local CSF-1 synthesis. *Blood* **98**, 74-84 (2001).

172 Passananti, C. *et al.* The product of Zfp59 (Mfg2), a mouse gene expressed at the spermatid stage of spermatogenesis, accumulates in spermatozoa nuclei. *Cell Growth and Differentiation-Publication American Association for Cancer Research* **6**, 1037 (1995).

173 Ishishita, S., Matsuda, Y. & Kitada, K. Genetic evidence suggests that Spata22 is required for the maintenance of Rad51 foci in mammalian meiosis. *Scientific reports* **4**, 6148 (2014).

174 Tokuda, M., Kadokawa, Y., Kurahashi, H. & Marunouchi, T. CDH1 is a specific marker for undifferentiated spermatogonia in mouse testes. *Biology of reproduction* **76**, 130-141 (2007).

175 Ishiguro, K. i., Kim, J., Fujiyama-Nakamura, S., Kato, S. & Watanabe, Y. A new meiosis-specific cohesin complex implicated in the cohesin code for homologous pairing. *EMBO reports* **12**, 267-275 (2011).
