# Supplementary material for: Characterization of germ cell differentiation in the male mouse through single-cell RNA sequencing
Source: Sci Rep. 2018 Apr 25;8:6521. doi: 10.1038/s41598-018-24725-0 (PMC5916943; doi:10.1038/s41598-018-24725-0)
Supplement: Supplementary file 4 — Supplementary data table 3 [file 41598_2018_24725_MOESM4_ESM.pdf]

Gene name	Early Sgonia	Late Sgonia	Early Scytes	Late Scytes	Round Stids	Later Stids	Sertoli	Leydig	Method	Source
Hsd3b6	0	0	0	0	0	0	0	1	In situ	[1]
Hsd17b11	0	0	0	0	0	0	0	1	In situ	[2]
Cyp17a1	0	0	0	0	0	0	0	1	IHC	[3]
Vcam1	0	0	0	0	0	0	0	1	IHC	[4]
Wt1	0	0	0	0	0	0	1	0	In situ	[5]
Rhox5	0	0	0	0	0	0	1	0	In situ	[6]
Kitl	0	0	0	0	0	0	1	0	In situ	[7]
Kitl	0	0	0	0	0	0	1	0	IHC	[7]
Sox8	0	0	0	0	0	0	1	0	RNASeq	[8]
Stra8	1	1	0	0	0	0	0	0	In situ	[9]
Stra8	1	1	0	0	0	0	0	0	IHC	[9]
Crabp1	1	1	0	0	0	0	0	0	IHC	[10]
Crabp1	1	1	0	0	0	0	0	0	Northern	[10]
Hist1h1a	0	0	1	1	0	0	0	0	In situ	[11]
Hist1h1a	0	0	1	1	0	0	0	0	IHC	[11]
Hist1h1a	1	1	0	0	0	0	0	0	In situ	[12]
Dazl	1	1	0	0	0	0	0	0	In situ	[13]
Dazl	1	1	1	1	0	0	0	0	IHC	[14]
Gpat2	0	0	1	0	0	0	0	0	In situ	[15]
Meiob	0	0	1	0	0	0	0	0	In situ	[16, 17]
Hist1h1t	0	0	1	1	1	1	NA	NA	qPCR	[18]
Hist1h1t	0	0	1	1	0	0	0	0	In situ	[19]
Hist1h1t	0	0	1	1	1	1	0	0	IHC	[19]
Hormad1	0	0	1	1	0	0	0	0	IHC	[20]
Hormad2	0	0	1	1	0	0	0	0	IHC	[20]
Meioc	0	0	1	1	0	0	0	0	IHC	[21]
Piwil2	1	1	1	1	0	0	NA	NA	qPCR	[18]
Piwil2	0	0	1	1	0	0	0	0	IHC	[22]
Pou5f2	0	0	0	1	0	0	0	0	In situ	[23]
Prss42	0	0	1	1	0	0	0	0	In situ	[24]
Prss42	0	0	0	0	1	1	0	0	IHC	[24]
Prss43	0	0	1	1	0	0	0	0	In situ	[24]
Prss43	0	0	1	1	1	1	0	0	IHC	[24]
Prss44	0	0	1	1	0	0	0	0	In situ	[24]
Tdrd1	1	1	1	1	1	1	NA	NA	qPCR	[18]
Tdrd1	0	0	1	1	0	0	0	0	In situ	[25]
Tdrd1	0	0	1	1	1	1	0	0	IHC	[25]
Zfp35	0	0	1	1	0	0	0	0	Northern	[26]
Klf17	0	0	0	0	1	0	0	0	In situ	[27]
Rad21	1	1	0	0	1	1	1	1	In situ	[28]
Esx1	1	1	0	0	1	0	0	0	In situ	[29]
Dyrk4	0	0	0	0	0	1	0	0	In situ	[30]
Prm1	0	0	0	0	1	1	NA	NA	qPCR	[18]
Prm1	0	0	0	0	0	1	0	0	In situ	[31]
Hspa1l	0	0	0	0	0	1	0	0	In situ	[32]
Hspa1l	0	0	0	0	1	1	0	0	IHC	[32]
Tnp1	0	0	0	0	1	1	0	0	In situ	[33]
Tnp1	0	0	0	0	0	1	0	0	IHC	[33]
Tnp2	0	0	0	0	1	1	0	0	In situ	[34]

1 Baker, P. J. *et al.* Expression of 3β-hydroxysteroid dehydrogenase type I and type VI isoforms in the mouse testis during development. *The FEBS Journal* **260**, 911-917 (1999).

2 O'Shaughnessy, P., Baker, P., Heikkila, M., Vainio, S. & McMahon, A. Localization of 17β-hydroxysteroid dehydrogenase/17-ketosteroid reductase isoform expression in the developing mouse testis—androstenedione is the major androgen secreted by fetal/neonatal Leydig cells. *Endocrinology* **141**, 2631-2637 (2000).

3 Le Goascogne, C. *et al.* Immunoreactive cytochrome P-45017α in rat and guineapig gonads, adrenal glands and brain. *Journal of reproduction and fertility* **93**, 609-622 (1991).

4 Sainio-Pöllänen, S. *et al.* CD106 (VCAM-1) in testicular immunoregulation. *Journal of reproductive immunology* **33**, 221-238 (1997).

5 Pelletier, J. *et al.* Expression of the Wilms' tumor gene WT1 in the murine urogenital system. *Genes & development* **5**, 1345-1356 (1991).

6 Lindsey, J. S. & Wilkinson, M. F. Pem: a testosterone-and LH-regulated homeobox gene expressed in mouse Sertoli cells and epididymis. *Developmental biology* **179**, 471-484 (1996).

7 Manova, K. *et al.* The expression pattern of the c-kit ligand in gonads of mice supports a role for the c-kit receptor in oocyte growth and in proliferation of spermatogonia. *Developmental biology* **157**, 85-99 (1993).

8 Roumaud, P., Hache, J. & Martin, L. J. Expression profiles of Sox transcription factors within the postnatal rodent testes. *Molecular and cellular biochemistry*, doi:10.1007/s11010-018-3302-3 (2018).

9 Oulad-Abdelghani, M. *et al.* Characterization of a premeiotic germ cell-specific cytoplasmic protein encoded by Stra8, a novel retinoic acid-responsive gene. *The Journal of cell biology* **135**, 469-477 (1996).

10 Zheng, W., Bucco, R. A., Schmitt, M. C., Wardlaw, S. A. & Ong, D. E. Localization of cellular retinoic acid-binding protein (CRABP) II and CRABP in developing rat testis. *Endocrinology* **137**, 5028-5035 (1996).

11 Rabini, S. *et al.* Spermatogenesis in mice is not affected by histone H1. 1 deficiency. *Experimental cell research* **255**, 114-124 (2000).

12 Sun, R. & Qi, H. Dynamic expression of combinatorial replication-dependent histone variant genes during mouse spermatogenesis. *Gene Expression Patterns* **14**, 30-41 (2014).

13 Niederberger, C., Agulnik, A. I., Cho, Y., Lamb, D. & Bishop, C. E. In situ hybridization shows that Dazla expression in mouse testis is restricted to premeiotic stages IV-VI of spermatogenesis. *Mammalian genome* **8**, 277-278 (1997).

14 Ruggiu, M. *et al.* The mouse Dazla gene encodes a cytoplasmic protein essential for gametogenesis. *Nature* **389**, 73 (1997).

15 Garcia-Fabiani, M. B. *et al.* Methylation of the Gpat2 promoter regulates transient expression during mouse spermatogenesis. *Biochemical Journal* **471**, 211-220 (2015).

16 Souquet, B. *et al.* MEIOB targets single-strand DNA and is necessary for meiotic recombination. *PLoS genetics* **9**, e1003784 (2013).

17 Kogo, H. *et al.* Screening of genes involved in chromosome segregation during meiosis I: toward the identification of genes responsible for infertility in humans. *Journal of human genetics* **55**, 293 (2010).

18 Wang, P. J., Page, D. C. & McCarrey, J. R. Differential expression of sex-linked and autosomal germ-cell-specific genes during spermatogenesis in the mouse. *Human molecular genetics* **14**, 2911-2918 (2005).

19 Drabent, B., Bode, C., Bramlage, B. & Doenecke, D. Expression of the mouse testicular histone gene H1t during spermatogenesis. *Histochemistry and cell biology* **106**, 247-251 (1996).

20 Wojtasz, L. *et al.* Mouse HORMAD1 and HORMAD2, two conserved meiotic chromosomal proteins, are depleted from synapsed chromosome axes with the help of TRIP13 AAA-ATPase. *PLoS genetics* **5**, e1000702 (2009).

21 Soh, Y. S. *et al.* Meioc maintains an extended meiotic prophase I in mice. *PLoS genetics* **13**, e1006704 (2017).

22 Kuramochi-Miyagawa, S. *et al.* Mili, a mammalian member of piwi family gene, is essential for spermatogenesis. *Development* **131**, 839-849 (2004).

23 Andersen, B. *et al.* Sperm 1: a POU-domain gene transiently expressed immediately before meiosis I in the male germ cell. *Proceedings of the National Academy of Sciences* **90**, 11084-11088 (1993).

24 Yoneda, R. *et al.* Three testis-specific paralogous serine proteases play different roles in murine spermatogenesis and are involved in germ cell survival during meiosis. *Biology of reproduction* **88** (2013).

25 Chuma, S. *et al.* Mouse Tudor Repeat-1 (MTR-1) is a novel component of chromatoid bodies/nuages in male germ cells and forms a complex with snRNPs. *Mechanisms of development* **120**, 979-990 (2003).

26 Cunliffe, V., Koopman, P., McLaren, A. & Trowsdale, J. A mouse zinc finger gene which is transiently expressed during spermatogenesis. *The EMBO journal* **9**, 197-205 (1990).

27 Yan, W., Burns, K. H., Ma, L. & Matzuk, M. M. Identification of Zfp393, a germ cell-specific gene encoding a novel zinc finger protein. *Mechanisms of development* **118**, 233-239 (2002).

28 Lee, J., Yokota, T. & Yamashita, M. Analyses of mRNA expression patterns of cohesin subunits Rad21 and Rec8 in mice: Germ cell-specific expression of rec8 mRNA in both male and female mice. *Zoological science* **19**, 539-544 (2002).

29 Branford, W. W. *et al.* Spx1, a novel X-linked homeobox gene expressed during spermatogenesis. *Mechanisms of development* **65**, 87-98 (1997).

30 Sacher, F., Möller, C., Bone, W., Gottwald, U. & Fritsch, M. The expression of the testis-specific Dyrk4 kinase is highly restricted to step 8 spermatids but is not required for male fertility in mice. *Molecular and cellular endocrinology* **267**, 80-88 (2007).

31 Caldwell, K. A. & Handel, M. A. Protamine transcript sharing among postmeiotic spermatids. *Proceedings of the National Academy of Sciences* **88**, 2407-2411 (1991).

32 Tsunekawa, N., Matsumoto, M., Tone, S., Nishida, T. & Fujimoto, H. The Hsp70 homolog gene, Hsc70t, is expressed under translational control during mouse spermiogenesis. *Molecular reproduction and development* **52**, 383-391 (1999).

33 Heidaran, M. A., Showman, R. M. & Kistler, W. S. A cytochemical study of the transcriptional and translational regulation of nuclear transition protein 1 (TP1), a major chromosomal protein of mammalian spermatids. *The Journal of cell biology* **106**, 1427-1433 (1988).

34 Shih, D. & Kleene, K. A study by in situ hybridization of the stage of appearance and disappearance of the transition protein 2 and the mitochondrial capsule seleno-protein mrnas during spermatogenesis in the mouse. *Molecular reproduction and development* **33**, 222-227 (1992).
