# Supplementary material for: Characterization of germ cell differentiation in the male mouse through single-cell RNA sequencing
Source: Sci Rep. 2018 Apr 25;8:6521. doi: 10.1038/s41598-018-24725-0 (PMC5916943; doi:10.1038/s41598-018-24725-0)
Supplement: Supplementary file 5 — Supplementary data table 4 [file 41598_2018_24725_MOESM5_ESM.pdf]

EnsemblID	GeneName	RS1 Average	RS1 Log2 Fold Change	RS1 P-Value	SC2 Average	SC2 Log2 Fold Change	SC2 P-Value	CS Average	CS Log2 Fold Change	CS P-Value	RS2 Average	RS2 Log2 Fold Change	RS2 P-Value	ES Average	ES Log2 Fold Change	ES P-Value	SC1 Average	SC1 Log2 Fold Change	SC1 P-Value	Sertoli Average	Sertoli Log2 Fold Change	Sertoli P-Value	Spg Average	Spg Log2 Fold Change	Spg P-Value	Leydig Average	Leydig Log2 Fold Change	Leydig P-Value
ENSMUSG00000079705	Ssxb1	1.97107793	4.16067064	4.19E-45	0.10154872	-2.95599507	4.57E-16	0.02912556	-4.34292847	2.71E-09	0.24193617	-1.42161408	0.0031104	0.02226847	-4.61164399	1.28E-07	0.0370227	-3.86918449	0.00020351	0.438319	-0.10526067	1	0	-1.39117294	1	0	-1.11404111	1
ENSMUSG00000023165	Ssxb2	1.83092372	4.04310159	6.20E-43	0.09533145	-2.95719557	4.90E-16	0.03495067	-4.01058006	1.49E-08	0.26942891	-1.15713689	0.01837556	0.00371141	-6.33384237	5.90E-10	0.02644479	-4.19645788	8.70E-05	0	-1.60271026	1	0	-1.30205882	1	0	-1.02492699	1
ENSMUSG00000078639	Gm12695	1.19640735	3.863569	6.90E-38	0.06631753	-2.88541958	9.28E-15	0.01456278	-4.5449471	4.67E-09	0.19794777	-0.99939566	0.05211836	0.03711412	-3.27747561	5.52E-05	0.01586687	-4.19688465	0.00015136	0	-1.01774776	1	0	-0.71709632	1	0	-0.43996449	1
ENSMUSG00000072722	Gm6588	3.06428082	3.36019027	1.80E-31	0.0455933	-4.93442692	3.20E-33	0.06990134	-3.93624558	6.31E-09	1.10337555	-0.18021147	0.78774143	0.04453695	-4.49870684	5.65E-08	0.04760061	-4.32960891	2.98E-05	0.2191595	-1.47229637	1	0	-2.17211069	1	0	-1.89497886	1
ENSMUSG00000090643	Gm3453	2.37242864	3.27307473	9.04E-30	0.20516986	-2.33858452	1.06E-11	0.0728139	-3.52678103	8.80E-08	0.57368197	-0.52494762	0.40090768	0.10763096	-2.93135455	9.88E-05	0.01586687	-5.30475963	2.47E-06	0	-2.12356822	1	0.28425658	-0.82232344	1	0	-1.54578495	1
ENSMUSG00000055102	Zfp819	1.13397502	3.34139469	4.97E-29	0.0455933	-3.46907824	5.36E-19	0.01456278	-4.5703697	4.19E-09	0.3574057	-0.05504758	1	0.02597989	-3.7662339	8.49E-06	0.04231166	-3.04603478	0.00281437	0	-1.04305885	1	0	-0.74240741	1	0	-0.46527558	1
ENSMUSG00000079389	Gm3149	1.3989939	3.2401918	4.82E-28	0.11501947	-2.4207356	8.72E-12	0.03786323	-3.6676356	1.12E-07	0.36290425	-0.41454665	0.57849248	0.06680542	-2.83543013	0.00024679	0.01586687	-4.54946373	4.50E-05	0	-1.36949616	1	0	-1.06884471	1	0	-0.79171288	1
ENSMUSG00000072726	Gm5797	2.40937839	3.11926038	2.29E-27	0.17201109	-2.67615386	1.60E-14	0.06116367	-3.83053763	1.28E-08	0.7551341	-0.13229118	1	0.11876519	-2.85267267	0.00012747	0.05817853	-3.77514655	0.0001639	0.2191595	-1.18284152	1	0	-1.8827593	1	0	-1.60562746	1
ENSMUSG00000090404	Gm8362	2.51258286	3.03492269	4.25E-26	0.20827849	-2.46945273	6.40E-13	0.0728139	-3.67042866	2.84E-08	0.80462104	-0.12154052	1	0.14103367	-2.69181934	0.00025999	0.04231166	-4.27455025	3.47E-05	0	-2.26580966	1	0	-1.96515822	1	0	-1.68802639	1
ENSMUSG00000079371	Gm3476	1.98127097	2.84617913	1.13E-22	0.17408352	-2.43404661	1.88E-12	0.05533856	-3.75973506	2.46E-08	0.744137	0.09354681	0.95070528	0.1521679	-2.28810933	0.00176769	0.02644479	-4.5703655	1.98E-05	0	-1.97564593	1	0	-1.67499449	1	0	-1.39786266	1
ENSMUSG00000098176	Ccdc166	1.36968893	2.87439201	1.31E-22	0.13367127	-2.25788041	8.74E-11	0.05242601	-3.28833735	6.45E-07	0.47104238	-0.04919209	1	0.05567118	-3.15258865	5.40E-05	0.0740454	-2.69765202	0.00581852	0	-1.4350178	1	0	-1.13436635	1	0	-0.85723452	1
ENSMUSG00000110310	4930518J21Rik	1.05752727	2.89686333	9.20E-22	0.01450696	-5.1052964	4.87E-29	0.02038789	-4.16537712	2.56E-08	0.54252352	0.68903112	0.09338312	0.03340271	-3.45436824	3.20E-05	0.01586687	-4.234786	0.00014973	0	-1.0555498	1	0	-0.75489835	1	0	-0.47776652	1
ENSMUSG00000108227	4933412L11Rik	0.0216602	-5.26770589	1.80E-21	1.18542585	1.45958717	6.58E-06	0.01747534	-5.19667401	2.08E-09	0.0146628	-5.60890412	6.37E-16	0.01113424	-5.61955028	9.22E-08	4.66486013	3.51576848	1.12E-14	1.53411651	1.1152305	0.73653419	0	-1.58900973	1	0	-1.3118779	1
ENSMUSG00000091692	4930433I11Rik	2.18130926	2.71433328	7.36E-21	0.09844008	-3.48702431	2.01E-21	0.05242601	-4.01422989	4.35E-09	0.98973886	0.38427178	0.40092398	0.3934097	-1.08169023	0.18283556	0.05288957	-3.87181102	0.00011699	0.2191595	-1.15350925	1	0.28425658	-0.85285781	1	0	-1.57630689	1
ENSMUSG00000043542	Zc2hc1a	1.45505559	2.71746602	1.76E-20	0.14299717	-2.29881067	3.09E-11	0.06116367	-3.20984362	7.87E-07	0.49486943	-0.124963	1	0.11134237	-2.32053945	0.00171628	0.26973681	-1.00653071	0.43225055	0.2191595	-0.56825621	1	0.56851316	0.31822972	1	0	-0.99134439	1
ENSMUSG00000091792	Gm3573	1.52258444	2.64382431	2.14E-19	0.09015039	-3.09944786	1.39E-17	0.06407623	-3.23468058	6.20E-07	0.7184771	0.42907827	0.3520629	0.23381897	-1.33724496	0.08268389	0.00528896	-5.83997256	1.23E-06	0	-1.6578199	1	0	-1.35716846	1	0	-1.08003663	1
ENSMUSG00000034689	4921530L21Rik	0.16054028	-3.91443998	2.62E-19	0.08911418	-4.88461044	1.72E-33	5.81346162	2.07991434	3.16E-08	2.16826125	0.26944098	0.6752309	8.7960471	2.86387512	6.83E-14	0.13751288	-3.79657997	0.00015683	0	-3.37498834	1	0	-3.0743369	1	0.73279041	-1.21174414	1
ENSMUSG00000093668	Pou5f2	0.08791492	-4.17467286	6.54E-19	3.58529148	4.46423406	3.46E-47	0.09320179	-3.83244925	6.50E-08	0.07148114	-4.32843848	4.19E-14	0.07051683	-4.17329754	1.28E-06	0.98374601	-0.33428922	1	0.438319	-1.18473805	1	0	-2.46980703	1	0	-2.1926752	1
ENSMUSG00000061525	4921509C19Rik	0.01783781	-4.69855353	2.05E-18	0.00828969	-5.85729021	9.52E-30	0.10193946	-1.96765853	0.00358214	0.93292052	1.86420338	1.75E-07	2.20829031	3.45419737	1.00E-18	0.02115583	-3.91907911	0.0008432	0	-1.06299278	1	0	-0.76234134	1	0	-0.48520951	1
ENSMUSG00000028332	Hemgn	0.19494177	-3.96131955	2.27E-18	0.11087462	-4.89681857	9.05E-32	4.11835407	0.96211635	0.0356871	1.26833202	-1.0122891	0.05506913	17.9001414	4.38462095	2.40E-14	0.30675951	-2.98774586	0.00351587	0	-3.69900795	1	0	-3.3983565	1	0	-3.12122467	1
ENSMUSG00000026429	Ube2t	0.15926615	-3.74768177	5.88E-18	3.65886249	2.29261777	2.94E-15	0.20970403	-3.10901228	1.08E-06	0.13196518	-3.88521246	1.56E-13	0.16701355	-3.39637801	1.18E-05	6.71168651	2.33686748	1.12E-06	0.87663801	-0.87761179	1	3.1268224	0.68804514	0.85665616	0.3663952	-1.62260054	1
ENSMUSG00000085940	4930405D11Rik	0.06370646	-4.08783689	5.88E-18	0.03937603	-4.89587027	2.30E-29	0.39610761	-1.17171079	0.08528871	1.55242373	1.1696174	0.00166018	5.67474937	3.88688334	2.40E-14	0.06346748	-3.70413273	0.00060695	0	-2.22823594	1	0.28425658	-0.92703269	1	0.3663952	-0.64990086	1

ENSMUSG00000111712	RP23-100M12.7	0.0522393	-3.99957785	7.68E-18	0.04455709	-4.34528734	5.93E-26	1.22618604	1.07674466	0.01022418	0.64149741	-0.02760028	1	4.30894965	3.81488196	4.62E-26	0.05817853	-3.45164572	0.0009345	0	-1.86148264	1	0	-1.5608312	1	0	-1.28369937	1
ENSMUSG00000022394	L3mbtl2	0.03822388	-4.02435463	7.68E-18	1.26521413	3.17161322	1.75E-28	0.05242601	-3.30086166	1.01E-06	0.03115845	-4.15602327	3.13E-13	0.03711412	-3.71043502	7.91E-06	1.13183681	1.33643317	0.01300038	1.31495701	1.36566018	0.71513975	1.42128291	1.44296787	0.77071655	0	-0.86961008	1
ENSMUSG00000059288	Cdyl	0.46760543	-3.58901608	9.56E-18	0.21864061	-4.81601542	1.47E-34	11.8278896	1.79471538	2.25E-06	6.831031	0.84437586	0.02947466	18.6053098	2.63904501	1.10E-11	0.41782761	-3.44512205	0.00035802	0.2191595	-3.59246017	1	1.13702633	-1.96955914	1	0.3663952	-3.01467689	1
ENSMUSG00000038025	Phf2	0.04586865	-4.00167603	9.60E-18	1.57607755	3.65586778	1.71E-35	0.02038789	-4.79348194	7.29E-10	0.03115845	-4.39163839	4.02E-14	0.0296913	-4.23617101	1.04E-06	1.0472135	0.94229365	0.11002921	0.2191595	-0.67977173	1	0	-1.37992694	1	0	-1.1027951	1
ENSMUSG00000003190	Bcl2l12	0.07007711	-3.79055553	1.70E-17	1.82269587	2.7318917	1.80E-22	0.04660089	-4.09347077	6.67E-09	0.04948694	-4.14295467	5.53E-14	0.04824836	-3.98885814	1.27E-06	2.49109878	1.95058609	6.86E-05	0.438319	-0.48605041	1	0.56851316	-0.18539897	1	0.3663952	-0.49384473	1
ENSMUSG00000075524	4930407I10Rik	0.05351343	-3.87189172	2.02E-17	0.0455933	-4.21902948	1.84E-25	1.86694834	2.02959025	6.52E-08	0.69831575	0.2343281	0.69248629	2.87634452	2.85145886	1.11E-13	0.09520123	-2.6917993	0.00693581	0.2191595	-0.76970055	1	0.28425658	-0.46904911	1	0	-1.1926752	1
ENSMUSG00000100916	Lhb	1.23208297	2.51467732	2.04E-17	0.29324783	-0.91071989	0.00810502	0.03203812	-3.91802298	2.35E-08	0.34091005	-0.54768659	0.37590941	0.04824836	-3.30707273	2.75E-05	0.03173374	-3.76498552	0.00027536	0	-1.39527696	1	0.28425658	-0.09364242	1	0	-0.81749369	1
ENSMUSG00000070332	Trim80	0.23443978	-3.59588118	3.10E-17	0.13988854	-4.46472199	2.76E-30	5.55133158	1.64609042	2.34E-05	2.92522819	0.52534285	0.25633482	10.8818608	2.99316506	2.40E-14	0.22742515	-3.32134837	0.00068896	0.2191595	-2.60674756	1	0.28425658	-2.30609612	1	0.3663952	-2.02896429	1
ENSMUSG00000084875	4930570D08Rik	0.55806861	-3.49132035	4.49E-17	0.20309744	-5.08533613	4.44E-37	26.7576512	3.65637426	2.17E-14	3.6217111	-0.49461242	0.38249417	11.6872373	1.47592947	0.00108088	0.48658405	-3.38711889	0.0004289	0	-4.75215765	1	0.28425658	-3.45141028	1	0	-4.17437438	1
ENSMUSG00000079269	Gm3676	1.98381922	2.44715287	5.10E-17	0.1450696	-2.85362753	4.48E-16	0.11650224	-2.84244156	4.33E-06	1.04289151	0.54442654	0.19225423	0.33773852	-1.2583638	0.10272419	0.02115583	-4.9643007	4.92E-06	0	-2.10566114	1	0	-1.8050097	1	0	-1.52787787	1
ENSMUSG00000031609	Sap30	0.04969104	-3.8859056	5.38E-17	1.37505254	2.67662082	1.04E-20	0.03203812	-4.20446578	1.19E-08	0.03115845	-4.39082399	5.10E-14	0.0593826	-3.31132426	4.57E-05	1.97278098	2.02169527	4.62E-05	0.6574785	0.32265093	0.95719131	0	-1.37912028	1	0.3663952	-0.10118135	1
ENSMUSG00000043460	Elfn2	0.1439766	-4.00030374	6.32E-17	2.56358703	0.85593634	0.01075143	0.38445738	-2.33150364	0.00053981	0.10447244	-4.32498935	2.09E-13	0.44908089	-2.07242101	0.01257893	13.7565773	3.78098084	4.38E-14	2.19159501	0.15883734	0.89333822	0	-3.00256401	1	0	-2.72543218	1
ENSMUSG00000031105	Slc25a14	0.17328158	3.30496096	7.77E-17	0.01243454	-2.51567816	5.57E-07	0.01456278	-1.83550257	0.03445211	0.0256599	-1.21223806	0.08356827	0.00371141	-3.06705583	0.00769067	0	-3.5262184	0.02225	1.31495701	4.5138617	0.06528944	0.28425658	2.96557912	0.77684587	0.3663952	3.24271095	0.89107103
ENSMUSG00000026743	Mllt10	0.49436214	-3.51499705	9.13E-17	9.48444303	2.2110431	2.94E-15	0.35824438	-3.75188604	1.41E-08	0.31341731	-4.05084759	1.20E-14	0.24866462	-4.23805196	1.23E-07	19.0349565	2.47584795	1.18E-07	3.94487102	-0.35084081	0.97633973	4.8323619	-0.12829856	0.96642635	1.46558082	-1.70054977	1
ENSMUSG00000048994	H2al3	0.25609998	-3.49691036	1.06E-16	0.15957656	-4.30329201	4.20E-29	9.7658	2.90649344	2.17E-14	1.69721886	-0.46952274	0.42582336	8.30614068	2.32011599	1.18E-08	0.36493804	-2.67689346	0.00521745	0.438319	-2.05222944	1	0.56851316	-1.751578	1	0	-3.05982413	1
ENSMUSG00000029909	Prss37	0.24081043	-3.59124279	1.06E-16	0.16268519	-4.27839437	7.59E-28	6.85033152	2.03553425	9.99E-08	1.03556011	-1.26892202	0.00959653	13.4798494	3.51801497	2.40E-14	0.32791534	-2.83371357	0.00414588	0.438319	-2.0555498	1	0.56851316	-1.75489835	1	0	-3.06314353	1
ENSMUSG00000109750	Hmgb1-rs17	0.16054028	-3.54091212	1.75E-16	0.09222282	-4.46417833	2.07E-29	8.67067897	3.97512328	2.17E-14	0.18695067	-3.18587564	2.72E-10	4.31266107	1.87210759	1.58E-05	0.19569141	-2.93383677	0.0027381	0.438319	-1.42510453	1	0.28425658	-1.70973643	1	0.3663952	-1.4326046	1
ENSMUSG00000033174	Mgll	0.12996118	-3.60971762	2.73E-16	2.65891848	2.18260026	3.23E-14	0.07863901	-4.07634936	7.48E-09	0.10813813	-3.74079404	2.63E-12	0.25237603	-2.37292833	0.00186453	5.25193436	2.43292279	4.99E-07	0.6574785	-0.77424209	1	0	-2.47472381	1	0.73279041	-0.61187414	1
ENSMUSG00000032238	Rora	0.03822388	-3.85309945	2.75E-16	0.03005013	-4.31577013	1.76E-24	0.90871745	1.26929509	0.00191378	0.43071969	-0.01704047	1	2.64252554	3.54145912	1.02E-21	0.1110681	-1.98072418	0.0587958	2.41075451	2.31700437	0.43015551	0.56851316	0.60802711	0.9819497	0.73279041	0.88515894	1
ENSMUSG00000090206	Tepp	0.16691093	-3.39464291	3.14E-16	0.09429524	-4.34373811	1.30E-29	3.37565231	1.59793126	3.16E-05	2.77126882	1.38075526	5.61E-05	4.88421856	2.24316877	3.00E-08	0.14280184	-3.29086245	0.00060191	0	-2.92446812	1	0	-2.62381668	1	0	-2.34668485	1
ENSMUSG00000028492	Saxo1	0.11084924	-3.57414	3.24E-16	0.05181057	-4.79254014	6.73E-31	0.39902016	-1.46026939	0.01903459	2.59531524	1.90787505	1.52E-08	5.45577605	3.16928299	2.40E-14	0.13751288	-2.92989067	0.00318546	0	-2.51362401	1	0	-2.21297257	1	0	-1.93584074	1
ENSMUSG00000051695	Pcbp1	0.05606169	-3.68765888	3.70E-16	1.28593836	2.4118455	2.06E-17	0.06116367	-3.29798107	9.43E-07	0.04765409	-3.77456894	5.79E-12	0.04824836	-3.57010714	1.32E-05	2.11029387	2.1758524	6.49E-06	1.75327601	1.52032093	0.6588593	0.28425658	-0.35470862	1	1.83197602	1.51067204	0.9269738
ENSMUSG00000070315	4930581F22Rik	0.04969104	-4.11218145	3.86E-16	1.81129754	3.52591412	2.74E-29	0.01165022	-5.69533347	2.93E-10	0.0219942	-5.08746518	1.46E-14	0.01113424	-5.63137857	7.82E-08	1.46504111	1.24014926	0.03702691	0.2191595	-0.90078027	1	0	-1.60082093	1	0	-1.3236891	1

ENSMUSG00000029828	4921507P07Rik	0.22934326	-3.40047421	4.12E-16	0.1543955	-4.09366782	2.87E-27	9.27649061	3.27264283	2.17E-14	0.92925482	-1.16055253	0.01594162	6.51723996	2.17798202	1.24E-07	0.26973681	-2.85552603	0.00281324	0.87663801	-1.0627453	1	0	-3.08501162	1	0	-2.80787979	1
ENSMUSG00000029752	Asns	0.1656368	-3.47924563	5.49E-16	0.1088022	-4.20734767	4.16E-27	2.30965684	0.80195552	0.06406241	1.69721886	0.30261749	0.61666147	9.0669802	3.6758608	2.40E-14	0.21684724	-2.77285837	0.00469976	2.19159501	0.46712206	0.86307533	0.56851316	-1.10929215	1	0	-2.41777122	1
ENSMUSG00000030137	Tuba8	0.23316565	-3.49056572	5.49E-16	0.14196096	-4.33068829	3.79E-28	5.75812305	1.87780927	1.24E-06	1.21701223	-0.85364587	0.1103035	12.2587948	3.53307039	2.40E-14	0.17982454	-3.54265889	0.00046184	0.2191595	-2.4965478	1	0	-3.19612538	1	0	-2.91899355	1
ENSMUSG00000070388	Fbxo39	0.09555969	-3.55609807	5.49E-16	0.07046238	-4.11359217	2.18E-25	2.44363442	1.84352825	1.17E-06	0.65066166	-0.50168139	0.45501357	5.08463482	3.41571401	2.40E-14	0.05288957	-4.00272591	0.00013873	0	-2.28450114	1	0.28425658	-0.983319	1	0	-1.70671787	1
ENSMUSG00000020974	Pole2	1.36968893	2.39515191	5.81E-16	0.06838995	-3.44013134	3.89E-20	0.03786323	-3.88988378	2.13E-08	0.92925482	1.02229689	0.00459083	0.0296913	-4.14505835	6.86E-07	0.0370227	-3.76683458	0.00024263	0.2191595	-0.58902722	1	0.56851316	0.29744624	1	0	-1.01210295	1
ENSMUSG00000024532	1700034E13Rik	0.70077108	-3.3110887	6.17E-16	0.48391073	-3.96829312	5.27E-27	28.2459673	3.47072679	2.17E-14	3.39993628	-0.77253653	0.11358814	14.9235888	1.73307795	6.37E-05	0.64525276	-3.13525467	0.00096576	1.31495701	-2.09748672	1	0.85276975	-2.60444905	1	0.73279041	-2.74244099	1
ENSMUSG00000034227	Foxj1	1.66146453	2.35972612	7.30E-16	0.15543171	-2.50772333	3.88E-13	0.10193946	-2.80512892	6.02E-06	0.95491472	0.67348256	0.08572729	0.11876519	-2.54622367	0.00051998	0.05817853	-3.47153548	0.00041626	0.2191595	-0.88056338	1	0.28425658	-0.57991194	1	0	-1.30348198	1
ENSMUSG00000085785	Sox5os3	0.11849402	-3.44888634	8.35E-16	0.0549192	-4.68174795	1.08E-30	1.53491697	0.70478855	0.11108875	1.72654446	1.01597596	0.00688698	5.52258147	3.2521443	2.40E-14	0.12164601	-3.07449246	0.00173468	0	-2.48712665	1	0.56851316	-0.60059027	1	0	-1.90934338	1
ENSMUSG00000071015	Gm136	0.17200745	-3.43916104	9.03E-16	0.09429524	-4.43181489	4.82E-29	7.22022613	3.30249889	2.17E-14	0.3244144	-2.37181658	9.26E-07	5.90856835	2.52508735	3.07E-10	0.11635706	-3.66304522	0.00028622	0.2191595	-2.01038787	1	0	-2.71005721	1	0	-2.43292538	1
ENSMUSG00000097287	D130017N08Rik	0.19366764	-3.61874094	9.44E-16	3.7324335	1.92284389	9.40E-11	0.09611435	-4.37790461	2.87E-09	0.12280093	-4.14543993	1.53E-13	0.07793966	-4.62240553	1.48E-07	9.66292456	2.85411002	1.10E-09	1.75327601	-0.18322102	0.95613306	0	-3.05451693	1	0	-2.7773851	1
ENSMUSG00000015962	1700016C15Rik	0.27648604	-3.37684946	1.08E-15	0.16993867	-4.20435896	7.45E-28	8.74640543	2.62477812	3.37E-13	0.54069067	-2.25356264	2.31E-06	11.8245595	3.16578601	2.40E-14	0.23271411	-3.31306412	0.00074429	0.6574785	-1.63052821	1	0.56851316	-1.74512297	1	0	-3.05337097	1
ENSMUSG00000102096	1700101O22Rik	0.34783728	-3.4022298	1.14E-15	0.19169911	-4.39012164	3.32E-29	2.05917704	-0.51634404	0.63383597	4.7049253	0.94085917	0.01511335	18.7686119	3.81103073	2.40E-14	0.24858098	-3.57595417	0.00036531	0.6574785	-1.98514492	1	0	-3.68498311	1	0.3663952	-2.40768809	1
ENSMUSG00000017720	Trp53tg5	0.35675619	-3.40191937	1.30E-15	0.20724228	-4.31264889	1.97E-28	8.87455789	2.02437848	1.15E-07	1.17852239	-1.47943078	0.00214381	17.8593159	3.57083135	2.40E-14	0.41253865	-2.88845599	0.00338382	0.438319	-2.43645117	1	0	-3.72108057	1	0.73279041	-1.8586679	1
ENSMUSG00000027674	Pex5l	0.24208456	-3.44023227	1.30E-15	0.13677991	-4.39023693	1.52E-28	1.2290986	-0.7961857	0.28984269	3.37427638	0.94705184	0.01517313	13.728514	3.88868325	2.40E-14	0.19040245	-3.46708604	0.00060659	0	-3.50180603	1	0.28425658	-2.20092636	1	0	-2.92402275	1
ENSMUSG00000028603	Scp2	0.77849296	2.42526394	1.37E-15	0.06838995	-2.56834132	2.51E-12	0.02330045	-3.70079226	2.59E-07	0.34457575	0.22198632	0.72171783	0.06680542	-2.22078361	0.00398731	0.19040245	0.6820495	0.76696744	1.53411651	2.24630838	0.48159326	1.13702633	1.86429578	0.71905949	3.29755684	3.14908931	0.38204546
ENSMUSG00000011350	Gm5893	0.09301143	-3.50973292	1.38E-15	0.06217268	-4.21022903	7.19E-26	4.96008273	3.98729255	2.03E-32	0.14296228	-2.74739052	4.51E-08	2.27880714	1.72608105	7.34E-05	0.17453558	-2.27778137	0.02300243	0.438319	-0.61485875	1	0	-1.90029472	1	0.73279041	-0.03707548	1
ENSMUSG00000037307	Banf2	0.32108057	-3.31748546	1.55E-15	0.17719215	-4.30407105	3.78E-29	2.96789448	0.29171582	0.71207069	4.24304716	1.00907707	0.00691835	14.4782193	3.42641996	2.40E-14	0.26444785	-3.28969282	0.00071984	0.2191595	-2.78834296	1	0.28425658	-2.48769152	1	0.3663952	-2.21055969	1
ENSMUSG00000047518	Slfnl1	0.68165914	-3.21413569	1.56E-15	0.38443443	-4.17133589	2.17E-29	21.9373712	2.97135805	2.17E-14	4.91387017	0.00145516	1	14.9013203	1.90859212	5.40E-06	0.57649632	-3.16314013	0.00075509	0.2191595	-3.77178387	1	0.28425658	-3.47113242	1	0.3663952	-3.19400059	1
ENSMUSG00000100872	1700065J18Rik	0.05096517	-3.63255597	1.68E-15	0.03108634	-4.45974885	4.96E-26	1.40385195	1.86911101	1.07E-06	0.43438539	-0.22762616	0.92181016	2.75386791	3.28213576	1.93E-18	0.06346748	-2.93937371	0.00407693	0	-1.46809774	1	0	-1.1674463	1	0	-0.89031447	1
ENSMUSG00000018322	Tomm34	0.04459452	-3.61432227	2.02E-15	0.98336463	2.43465772	1.37E-17	0.04077578	-3.45908637	5.54E-07	0.05681834	-3.12354733	3.01E-09	0.14845649	-1.59325793	0.04085846	1.30637239	1.80282632	0.00035995	3.28739252	2.75367189	0.29424288	1.70553949	1.85190459	0.68549806	0.3663952	0.31628017	1
ENSMUSG00000038997	Asb17	0.53131189	-3.21512067	2.03E-15	0.2631977	-4.36274136	6.33E-31	17.1491293	2.97645736	2.17E-14	4.10741628	0.1228107	0.95183163	11.1861966	1.83332439	1.59E-05	0.40196074	-3.32101282	0.00046448	0.6574785	-2.41370836	1	0.56851316	-2.52821569	1	0.73279041	-2.25108386	1
ENSMUSG00000031971	Ccsap	0.39752832	-3.24139174	2.04E-15	0.21553197	-4.25536468	1.78E-29	14.0414321	3.18932696	2.17E-14	1.805357	-0.8054533	0.09496448	9.90204796	2.13082862	2.22E-07	0.33849325	-3.17243514	0.00085188	0.87663801	-1.69948558	1	0.56851316	-2.13611807	1	0.73279041	-1.85898624	1
ENSMUSG00000078935	1700025C18Rik	0.01783781	-4.03642316	2.21E-15	0.02279665	-3.81808681	5.01E-19	0.77765243	2.18010517	1.85E-08	0.26209751	0.17311231	0.83288006	1.06517532	2.73257805	1.50E-11	0.04231166	-2.4083241	0.02452037	0	-0.41091608	1	0	-0.11026464	1	0	0.16686719	1

ENSMUSG00000027956	Tmem144	0.11467163	-3.52531152	2.30E-15	0.05699163	-4.65623021	8.34E-29	0.34950671	-1.65889001	0.00884949	2.1334371	1.43200755	7.79E-05	6.47270301	3.66453333	2.40E-14	0.06875644	-3.88425313	0.00026915	0.438319	-0.92866151	1	0	-2.21387765	1	0	-1.93674581	1
ENSMUSG00000027955	Fam198b	0.02930497	-3.74991354	2.66E-15	0.02383286	-4.16419009	1.03E-22	1.02813224	2.18569285	7.56E-09	0.3720685	0.30397234	0.57776899	1.36951113	2.67345883	1.73E-11	0.02644479	-3.39994634	0.00147231	0	-0.80933136	1	0	-0.50867992	1	0.3663952	0.76992782	1
ENSMUSG00000052099	Prss51	0.09938208	-3.52405579	2.67E-15	0.06528132	-4.25014147	1.40E-25	4.16495497	3.10490335	2.17E-14	0.17961928	-2.52258683	5.64E-07	3.97492255	2.73139542	1.10E-11	0.12693497	-2.83576952	0.00556963	0.2191595	-1.30870447	1	0	-2.0085747	1	0	-1.73144287	1
ENSMUSG00000061474	Mrps36	0.25609998	-3.26590097	2.74E-15	0.2994651	-3.136218	1.13E-18	3.82127337	1.18578603	0.0040538	2.09861296	0.16227074	0.89015818	10.3808201	3.22096199	2.40E-14	0.98903497	-0.99612754	0.4494665	1.09579751	-0.82765387	1	2.84256582	0.34868098	0.91913165	0.3663952	-1.83580379	1
ENSMUSG00000105023	1700012D16Rik	0.03949801	-3.69290696	2.87E-15	0.04144846	-3.73898426	2.81E-20	2.23101783	3.65519179	6.75E-26	0.12096808	-1.91984431	0.00012958	1.26188017	1.96837578	5.50E-06	0.04760061	-3.01977311	0.00392488	0.2191595	-0.16796234	1	0	-0.86846091	1	0.3663952	0.40982093	1
ENSMUSG00000055882	Abhd16b	0.07644775	-3.43773467	2.92E-15	0.04248467	-4.40438452	2.64E-27	2.89216803	2.96572224	2.17E-14	0.3427429	-1.051862	0.03655246	2.67963966	2.55372106	8.77E-11	0.04760061	-3.70679065	0.00033387	0.438319	-0.26580966	1	0	-1.55155339	1	0	-1.27442156	1
ENSMUSG00000007907	Cabs1	1.27030685	-3.13940189	2.92E-15	0.72223935	-4.08603607	2.80E-28	48.4416301	3.68673675	2.17E-14	4.93953006	-0.94317668	0.0439117	21.4371173	1.51459586	0.00071853	1.00490184	-3.19204426	0.00078096	2.41075451	-2.01113624	1	0.28425658	-4.29598161	1	2.56476643	-2.01852922	1
ENSMUSG00000109657	1700008N11Rik	0.10702686	-3.40116761	3.24E-15	0.05181057	-4.57076078	4.57E-29	1.59608064	1.00842994	0.01534196	1.30865472	0.72698047	0.06716987	4.90648703	3.29233131	2.40E-14	0.1110681	-3.00804167	0.00245392	0	-2.29560088	1	0	-1.99494944	1	0	-1.71781761	1
ENSMUSG00000048077	H1fnt	1.6385302	-3.06776021	3.85E-15	0.69633407	-4.44486053	1.22E-32	45.1970428	2.81730906	2.17E-14	12.3479087	0.25585167	0.6851889	32.4117634	1.90607612	4.96E-06	1.27992761	-3.14227274	0.00071984	1.31495701	-3.08705104	1	1.13702633	-3.27191333	1	1.46558082	-2.9947815	1
ENSMUSG00000066383	Iqcf1	1.28687053	-3.07571756	4.08E-15	0.69633407	-4.09631218	5.64E-29	34.3186464	2.71275811	2.17E-14	9.26138976	0.1632121	0.88043994	27.798478	2.07017809	4.61E-07	1.10010307	-3.01819889	0.00117424	0.6574785	-3.55391072	1	0.28425658	-4.25336933	1	0	-4.97629252	1
ENSMUSG00000041255	Tmco5b	0.05733582	-3.68509637	4.10E-15	0.04144846	-4.26949898	9.13E-24	0.40193272	-0.56961713	0.59816675	0.62133606	0.1311958	0.89376005	4.53163439	4.48249743	4.94E-35	0.05288957	-3.40088959	0.00179763	0	-1.68540883	1	0.28425658	-0.38395344	1	0	-1.10762556	1
ENSMUSG00000103328	Gm37406	0.02548258	-3.94557681	4.15E-15	0.02797771	-3.93422445	1.09E-19	0.56503585	1.0002615	0.02550178	0.17961928	-0.92076323	0.10755872	2.34561256	4.26798492	1.19E-30	0.02115583	-3.66298075	0.00144666	0	-0.80785546	1	0.28425658	0.4942734	1	0	-0.23007218	1
ENSMUSG00000032679	Cd59a	0.04459452	-3.65576307	4.15E-15	0.03937603	-3.9513337	9.44E-22	0.43979594	-0.00601607	1	0.63966456	0.67518606	0.10765137	2.95057276	3.84412506	8.90E-26	0.04231166	-3.30759458	0.00193614	0	-1.30295355	1	0	-1.00230211	1	0	-0.72517028	1
ENSMUSG00000001366	Fbxo9	0.04332039	-3.67146495	4.15E-15	0.76783265	1.42809187	1.92E-06	0.06698879	-2.78644835	3.25E-05	0.04948694	-3.33591064	1.31E-09	0.20041626	-1.16945022	0.18482162	2.40118651	2.95893258	4.73E-11	0.6574785	0.7256156	0.89735598	3.69533557	2.84434837	0.40908415	1.09918561	1.30339888	1
ENSMUSG00000108489	Gm45025	0.01528955	-4.05249202	4.35E-15	0.01554317	-4.16040845	2.60E-20	0.38445738	1.04427383	0.01830423	0.35374	0.99346199	0.011185	1.08373239	3.10331686	2.83E-15	0.02644479	-2.8068057	0.00993904	0	-0.21993661	1	0	0.08071483	1	0	0.35784666	1
ENSMUSG00000061762	Tac1	0.04969104	-3.52192077	4.52E-15	0.02797771	-4.4634536	3.22E-26	1.3019125	1.91985307	4.05E-07	0.7478027	0.95427182	0.01113738	1.75549801	2.42780948	1.74E-09	0.01586687	-4.50466226	6.61E-05	0	-1.32478935	1	0	-1.02413791	1	0	-0.74700608	1
ENSMUSG00000060161	Klk1b7-ps	0.08918905	-3.33562055	4.52E-15	0.03523119	-4.79465411	7.23E-31	1.46792818	1.26655044	0.00132204	1.59091358	1.62430541	1.07E-06	2.57943153	2.28938951	1.15E-08	0.08462331	-3.05728623	0.00176666	0.2191595	-0.97169695	1	0.28425658	-0.6710455	1	0	-1.39457259	1
ENSMUSG00000049506	Sppl2c	0.28795321	-3.19627936	4.75E-15	0.18444563	-3.9644977	1.56E-26	8.79009377	2.85055071	2.17E-14	1.55608943	-0.4724061	0.47941145	7.5564354	2.29979766	1.15E-08	0.36493804	-2.55298274	0.00674865	0.2191595	-2.51475527	1	0.28425658	-2.21410383	1	0.3663952	-1.936972	1
ENSMUSG00000028333	Anp32b	0.05988407	-3.88947561	5.11E-15	0.90564877	0.60480073	0.09051709	0.04368834	-4.05489625	2.22E-07	0.03665699	-4.43539795	1.63E-12	0.04824836	-3.86231543	2.86E-05	5.7649632	3.98161359	4.38E-14	2.41075451	1.64592671	0.60666498	21.0349871	4.63354393	0.07009731	2.19837123	1.44273556	0.89896545
ENSMUSG00000064037	Gpn1	1.23080884	2.31443481	5.13E-15	0.25594422	-1.23322126	0.00026069	0.04368834	-3.56973956	1.28E-07	0.39956124	-0.36521079	0.65973246	0.05567118	-3.18316346	4.37E-05	0.39138282	-0.34844732	1	0.6574785	0.53751951	0.93881335	0.56851316	0.42219572	1	0	-0.88750858	1
ENSMUSG00000043429	Ccdc185	0.28285669	-3.26146898	5.33E-15	0.16890246	-4.1324886	3.45E-27	5.48143024	1.6969556	1.25E-05	2.31488923	0.1659655	0.8851521	10.9783575	3.11390356	2.40E-14	0.2961816	-2.88994788	0.0029229	0	-3.55225897	1	0.28425658	-2.25138715	1	0.3663952	-1.97425532	1
ENSMUSG00000008734	Gprc5b	0.10065621	-3.31693903	5.36E-15	0.06424511	-4.08622754	9.75E-26	3.29410075	2.79951874	2.17E-14	0.7294742	-0.12380162	1	2.75757932	2.20531644	5.50E-08	0.0740454	-3.39430094	0.0006444	1.53411651	0.8776187	0.80633589	0.56851316	-0.23973282	1	0	-1.54874797	1
ENSMUSG00000091017	Fam71a	0.16181441	-3.33525366	6.23E-15	0.07564343	-4.5603229	1.65E-29	2.81935413	1.38505592	0.00071665	0.64882881	-1.11009998	0.0267478	8.89996664	4.02254073	2.40E-14	0.13222393	-3.29591475	0.00102386	0	-2.82254698	1	0.28425658	-1.52153007	1	0.3663952	-1.24439824	1

ENSMUSG00000036924	Cst13	0.40262484	-3.32869143	6.71E-15	0.24350968	-4.18101627	9.55E-27	8.58621485	1.80303894	4.52E-06	1.7943599	-0.92998356	0.0595747	19.1211961	3.56118133	2.40E-14	0.52360675	-2.64912505	0.00810512	0.2191595	-3.12460597	1	1.42128291	-1.23839912	1	0	-3.54697089	1
ENSMUSG00000029423	Piwil1	0.23316565	-3.44747556	6.71E-15	3.04128049	1.03276293	0.00101781	0.14271524	-3.91806259	3.07E-08	0.18511782	-3.65328326	1.47E-11	0.13732225	-3.93026399	2.91E-06	14.8408135	3.70184261	4.38E-14	1.75327601	-0.28314453	0.97206678	0	-3.1543052	1	0	-2.87717337	1
ENSMUSG00000052025	Ppp1r2-ps7	0.32490296	-3.20204803	7.04E-15	0.19895259	-4.03685281	7.66E-27	11.8220645	3.36552981	2.17E-14	1.26649917	-1.00908135	0.04176406	7.27065665	1.96706995	3.46E-06	0.32791534	-2.88727828	0.00257622	0.438319	-2.10866113	1	0.28425658	-2.39317197	1	0.3663952	-2.11604014	1
ENSMUSG00000079579	Gm6760	0.44084872	-3.15707144	8.40E-15	0.22900272	-4.23528889	3.94E-29	16.3860396	3.54396934	2.17E-14	2.06745451	-0.66109964	0.19890134	7.79396578	1.57573173	0.00038524	0.39138282	-3.03237717	0.00144704	0.87663801	-1.76696875	1	0.28425658	-2.78870106	1	0	-3.51172109	1
ENSMUSG00000085286	Ube4bos3	0.05478756	-3.42935813	8.40E-15	0.03005013	-4.41051062	3.33E-26	1.5989932	2.30173257	4.74E-10	0.66715731	0.66320135	0.10295658	1.68126976	2.26472796	2.90E-08	0.03173374	-3.74210881	0.00037949	0	-1.37249448	1	0	-1.07184304	1	0	-0.79471121	1
ENSMUSG00000058717	Gm4763	0.08536666	-3.52485276	8.40E-15	1.40199404	1.54618909	3.44E-07	0.06116367	-3.73995103	1.60E-07	0.08797679	-3.3442795	6.74E-10	0.05195977	-3.91018698	5.41E-06	4.63841535	3.16391651	9.43E-13	0.2191595	-1.09299261	1	0	-1.79294696	1	0	-1.51581513	1
ENSMUSG00000027442	Cst8	0.86640788	-3.07445201	1.09E-14	0.46525892	-4.10596766	5.72E-29	23.1810326	2.72236925	2.17E-14	6.52677794	0.24609693	0.70710535	17.9149871	1.9834771	1.74E-06	0.69814233	-3.09963378	0.0008979	1.09579751	-2.39740471	1	1.13702633	-2.35986944	1	10.6254609	0.50427063	1
ENSMUSG00000031197	Vbp1	0.27138953	2.60550815	1.10E-14	0.06217268	-0.92468288	0.02030586	0.01456278	-2.69464004	0.00045337	0.04032269	-1.45140969	0.01081705	0.01484565	-2.57571497	0.00486429	0.01057791	-2.77285837	0.01995085	0.438319	2.41011378	0.64983273	1.13702633	3.45691998	0.37238172	1.46558082	3.73405182	0.33340925
ENSMUSG00000072919	Noxred1	0.0828184	-3.39394002	1.17E-14	0.04766572	-4.31131866	5.93E-26	3.48341688	3.38394977	2.17E-14	0.11363668	-2.79804325	3.98E-08	2.66479402	2.4320435	2.14E-09	0.14280184	-2.28054516	0.02400184	0.438319	-0.33712015	1	0	-1.62279482	1	0	-1.34566299	1
