# Supplementary material for: Characterization of germ cell differentiation in the male mouse through single-cell RNA sequencing
Source: Sci Rep. 2018 Apr 25;8:6521. doi: 10.1038/s41598-018-24725-0 (PMC5916943; doi:10.1038/s41598-018-24725-0)
Supplement: Supplementary file 6 — Supplementary data table 5 [file 41598_2018_24725_MOESM6_ESM.pdf]

EnsemblID	GeneName	Sertoli Average	Sertoli Log2 Fold Change	Sertoli P-Value	Spermatogonia Average	Spermatogonia Log2 Fold Change	Spermatogonia P-Value	Leydig Average	Leydig Log2 Fold Change	Leydig P-Value
ENSMUSG00000025789	St8sia2	1.99	12.43	1.62E-03	0	6.43	1.00E+00	0	6.7	1.00E+00
ENSMUSG00000108077	6330415B21Rik	1.71	12.24	6.95E-03	0	6.62	1.00E+00	0	6.89	1.00E+00
ENSMUSG00000042429	Adora1	7.12	11.54	1.31E-04	0	4.47	1.00E+00	0	4.75	1.00E+00
ENSMUSG00000031853	Map3k21	1.42	11.01	8.47E-04	0	6.62	1.00E+00	0	6.89	1.00E+00
ENSMUSG00000031342	Gpm6b	11.11	10.84	1.30E-04	0	3.67	1.00E+00	0.37	4.97	3.03E-01
ENSMUSG00000037035	Inhbb	3.42	10.81	2.83E-04	0	5.34	1.00E+00	0	5.61	1.00E+00
ENSMUSG00000040102	Klhl42	1.14	10.75	5.62E-03	0	6.84	1.00E+00	0	7.12	1.00E+00
ENSMUSG00000044400	Sowahd	1.71	10.65	1.91E-03	0	6.26	1.00E+00	0	6.53	1.00E+00
ENSMUSG00000044667	Plppr4	1.71	10.65	2.80E-03	0	6.26	1.00E+00	0	6.53	1.00E+00
ENSMUSG00000043753	Dmrta1	2.28	10.6	6.21E-04	0	5.84	1.00E+00	0	6.12	1.00E+00
ENSMUSG00000024867	Pip5k1b	4.56	10.52	1.31E-04	0	4.84	1.00E+00	0	5.12	1.00E+00
ENSMUSG00000072966	Gprasp2	3.13	10.43	3.07E-04	0	5.34	1.00E+00	0	5.61	1.00E+00
ENSMUSG00000112287	RP24-528H20.6	1.99	10.43	1.13E-03	0	5.97	1.00E+00	0	6.24	1.00E+00
ENSMUSG00000032796	Lama1	1.42	10.43	1.27E-03	0	6.43	1.00E+00	0	6.7	1.00E+00
ENSMUSG00000024176	Sox8	1.42	10.43	2.16E-03	0	6.43	1.00E+00	0	6.7	1.00E+00
ENSMUSG00000026872	Zeb2	2.56	10.43	4.42E-04	0	5.62	1.00E+00	0.73	7.7	7.84E-02
ENSMUSG00000039910	Cited2	4.56	10.35	1.52E-04	0	4.78	1.00E+00	1.47	7.63	4.66E-02
ENSMUSG00000022425	Enpp2	3.42	10.32	7.71E-04	0	5.18	1.00E+00	0	5.45	1.00E+00
ENSMUSG00000045763	Basp1	4.56	10.19	1.31E-04	0	4.72	1.00E+00	0	5	1.00E+00
ENSMUSG00000002602	Axl	5.41	10.16	3.94E-04	0	4.47	1.00E+00	0	4.75	1.00E+00
ENSMUSG00000016239	Lonrf3	1.71	7.53	1.48E-02	3.7	8.98	2.31E-03	0	4.7	1.00E+00
ENSMUSG00000042105	Inpp5f	0.57	7.43	5.83E-02	1.42	8.84	1.57E-02	0	5.89	1.00E+00
ENSMUSG00000040147	Maob	0	7.43	1.00E+00	0	7.43	1.00E+00	1.1	11.7	2.64E-03
ENSMUSG00000110982	RP24-374N16.7	0	7.43	1.00E+00	0	7.43	1.00E+00	1.1	11.7	6.02E-03
ENSMUSG00000039114	Nrn1	0	7.43	1.00E+00	0	7.43	1.00E+00	1.1	11.7	7.71E-03
ENSMUSG00000032717	Mdfi	0	7.43	1.00E+00	0	7.43	1.00E+00	1.1	11.7	6.02E-03
ENSMUSG00000049307	Fut4	0	7.43	1.00E+00	0	7.43	1.00E+00	1.1	11.7	2.41E-03
ENSMUSG00000060882	Kcnd2	0	7.43	1.00E+00	0	7.43	1.00E+00	1.1	11.7	2.64E-03
ENSMUSG00000035226	Rims4	0	7.11	1.00E+00	0	7.1	1.00E+00	1.47	12.02	2.17E-03
ENSMUSG00000041936	Agrn	0.28	6.97	1.44E-01	1.71	9.65	2.06E-02	0	6.12	1.00E+00
ENSMUSG00000049047	Armcx3	0	6.84	1.00E+00	0	6.84	1.00E+00	1.83	12.29	1.28E-03
ENSMUSG00000025666	Tmem47	0	6.84	1.00E+00	0	6.84	1.00E+00	1.47	11.02	3.15E-03
ENSMUSG00000097124	A530020G20Rik	0	6.84	1.00E+00	0	6.84	1.00E+00	1.83	12.29	1.55E-03
ENSMUSG00000038679	Trps1	0	6.84	1.00E+00	0	6.84	1.00E+00	1.47	11.02	3.47E-03
ENSMUSG00000045659	Plekha7	0.28	6.73	1.30E-01	1.99	9.62	3.58E-02	0	5.89	1.00E+00
ENSMUSG00000035258	Abi3bp	0	6.62	1.00E+00	0	6.62	1.00E+00	1.83	11.29	3.78E-03
ENSMUSG00000035948	Acss3	0	6.62	1.00E+00	0	6.62	1.00E+00	1.83	11.29	2.29E-03
ENSMUSG00000009248	Ascl2	0	6.62	1.00E+00	1.14	10.16	3.25E-02	0.37	8.12	1.08E-01
ENSMUSG00000000058	Cav2	0	6.62	1.00E+00	0	6.62	1.00E+00	1.83	11.29	8.11E-03
ENSMUSG00000036098	Myrf	0	6.43	1.00E+00	1.14	9.75	1.11E-02	0	6.7	1.00E+00
ENSMUSG00000074207	Adh1	0	6.26	1.00E+00	0	6.26	1.00E+00	2.56	11.7	7.36E-04
ENSMUSG00000039601	Rcan2	0	6.26	1.00E+00	0	6.26	1.00E+00	2.2	10.92	5.95E-03

ENSMUSG00000002870	Mcm2	0.28	6.18	2.01E-01	3.7	10.43	1.70E-03	0	5.38	1.00E+00
ENSMUSG00000018604	Tbx3	0	6.11	1.00E+00	0	6.1	1.00E+00	2.56	11.12	4.03E-03
ENSMUSG00000028040	Efna4	0	5.97	1.00E+00	1.14	8.94	4.54E-02	0	6.24	1.00E+00
ENSMUSG00000034832	Tet3	0	5.84	1.00E+00	1.42	9.2	2.67E-02	0.73	7.97	6.77E-02
ENSMUSG00000002028	Kmt2a	0.57	5.69	1.63E-01	4.55	8.81	1.73E-03	0.37	5.34	3.63E-01
ENSMUSG00000032291	Crabp1	0.28	5.62	2.56E-01	4.26	9.62	1.73E-03	0	4.84	1.00E+00
ENSMUSG00000036109	Mbnl3	0	5.62	1.00E+00	1.42	8.84	2.41E-02	0	5.89	1.00E+00
ENSMUSG00000019979	Apaf1	0	5.62	1.00E+00	1.42	8.84	2.06E-02	0	5.89	1.00E+00
ENSMUSG00000053559	Smagp	0	5.52	1.00E+00	2.27	9.79	9.91E-03	0.37	6.89	1.78E-01
ENSMUSG00000060177	Klk1b22	0	5.43	1.00E+00	0	5.43	1.00E+00	4.76	11.92	1.62E-03
ENSMUSG00000108981	Gm15262	0	5.18	1.00E+00	2.27	9.14	6.96E-03	0	5.45	1.00E+00
ENSMUSG00000038521	C1s1	0	4.97	1.00E+00	0	4.97	1.00E+00	5.5	10.89	7.35E-04
ENSMUSG00000055612	Cdca7	0	4.9	1.00E+00	2.84	9.18	1.73E-03	0	5.18	1.00E+00
ENSMUSG00000044150	A830080D01Rik	0	4.62	1.00E+00	2.84	8.71	8.93E-03	0	4.89	1.00E+00
ENSMUSG00000041020	Map7d2	0	4.47	1.00E+00	3.13	8.69	6.98E-03	0	4.75	1.00E+00
ENSMUSG00000029177	Cenpa	0	3.38	1.00E+00	12.79	10.56	9.03E-04	0	3.66	1.00E+00
ENSMUSG00000029848	Stra8	0	3.3	1.00E+00	8.81	9.14	3.18E-02	0	3.57	1.00E+00
ENSMUSG00000005268	Prlr	0	2.55	1.00E+00	0	2.54	1.00E+00	31.88	11.21	2.59E-04
