# Supplementary material for: Characterization of germ cell differentiation in the male mouse through single-cell RNA sequencing
Source: Sci Rep. 2018 Apr 25;8:6521. doi: 10.1038/s41598-018-24725-0 (PMC5916943; doi:10.1038/s41598-018-24725-0)
Supplement: Supplementary file 7 — Supplementary data table 6 [file 41598_2018_24725_MOESM7_ESM.pdf]

QC data

	mouse 1	mouse 2
Estimated cell count	1,237	1,315
Number of reads produced	199,284,287	198,383,896
Mean reads per cell*	53,003	55,970
% reads mapped to transcriptome	62.1	64.6
% mapped to exons	66.7	68.7
% mapped intronic	4.0	3.9
% mapped antisense	5.0	4.8
% mapped intergenic	4.7	4.4
% Sequencing saturation**	31.2	30.4
Median UMIs per cell	17,871	20,456
Median genes per cell	4,405	4,711
Total genes detected	25,540	26,070
% Q30 bases in RNA read	82.5	83.8

* only counting reads in cells
** percent
